# Copper-induced stress mechanisms in *Erwinia amylovora*: a comparative phenotypic and transcriptomic study using copper-sensitive and -tolerant strains

**DOI:** 10.1128/aem.00334-25

**Published:** 2025-09-18

**Authors:** Ricardo Delgado Santander, Srđan G. Aćimović

**Affiliations:** 1 Plant Pathology and Plant-Microbe Biology Section, Hudson Valley Research Laboratory - Cornell University 5922, Highland, New York, USA; 2 School of Plant and Environmental Sciences, Alson H. Smith Jr. Agricultural Research and Extension Center - Virginia Polytechnic Institute and State University 1757, Winchester, Virginia, USA; The University of Tennessee Knoxville, Knoxville, Tennessee, USA

**Keywords:** fire blight, copper sensitivity, RNA-seq, copper homeostasis, copper-shock, copper adaptation, oxidative stress

## Abstract

*Erwinia amylovora* causes fire blight of apple and pear. Among the management strategies, copper-based treatments are widely used to control the disease. However, the impact of copper on the pathogen is not uniform, and different strains show different tolerance levels to the heavy metal. Upon identifying *E. amylovora* strains with unusually high (EaR2 and Ea17) and intermediate (Ea19) copper sensitivity, we conducted phenotypic and transcriptomic analyses to understand the basis of these differences. The highly copper-sensitive strains EaR2 and Ea17 grew slower, showed increased sensitivity to paraquat and cadmium, and developed a characteristic copper-dependent overproduction of amylovoran and levan, with patterns not observed in strain Ea273, with regular copper tolerance. Copper sensitivity was also associated with higher copper-shock death rates after copper pre-exposure during growth. Transcriptomic analysis via RNA-Seq revealed similar responses to copper shock in EaR2 and Ea273 but very different transcriptomic responses during copper adaptation (prolonged growth with copper). EaR2 responded to copper adaptation with earlier activation of stress responses, exopolysaccharide biosynthesis pathways, and protein quality control systems, while reducing the expression of genes linked to iron uptake. Ea273 mostly displayed an activation of copper homeostasis-related genes, with a characteristic downregulation of histidine catabolism.

**IMPORTANCE:**

This study identified and characterized, for the first time, copper hypersensitive *E. amylovora* strains, providing critical insights into the mechanisms of copper homeostasis and detoxification in the fire blight pathogen. Our study contributes to a broader understanding of bacterial adaptation to copper as well as the connection between phenotypic traits and transcriptomic responses to copper. Furthermore, our findings set the basis for future optimization of copper-based treatments and future development of more effective disease control methods.

## INTRODUCTION

*Erwinia amylovora* causes fire blight, a systemic bacterial disease with a disastrous economic impact in regions where apple and pear crops are cultivated (1–3). The pathogen infects plants by entering through natural openings or wounds. Following entry, the bacteria multiply and spread through the vascular system and apoplast. This invasion leads to characteristic fire blight symptoms, including necrosis, ooze secretion, and cankers. Severe infections can kill entire trees, particularly in highly susceptible hosts, in juvenile trees, and under favorable environmental conditions for the pathogen (4). The aggressive nature of *E. amylovora* and its ability to adapt to diverse environmental conditions makes fire blight particularly challenging to manage (5).

Copper-based materials have long been a cornerstone of fire blight management (6, 7). These compounds have a broad-spectrum antimicrobial activity, affordability, and effectiveness in controlling a variety of bacterial and fungal pathogens (8, 9). The toxicity of copper-based compounds is linked to their capacity to disrupt bacterial cell integrity by displacing iron from metalloproteins, generating reactive oxygen species (ROS), damaging membranes, as well as interfering with essential enzymatic functions and biomolecules (10). In *E. amylovora*, copper-based compounds have been reported to cause damage to the membrane structure, membrane potential, cytoplasmic pH, and respiration (11, 12). Exposure to copper can also lead to a loss of culturability in this pathogen, but cells can recover from this state within host tissues (12, 13).

Despite its toxicity, copper is an essential micronutrient required in trace concentrations for maintaining key cellular functions. Therefore, bacteria naturally possess copper homeostasis systems to regulate intracellular copper concentrations (14) (15). The extensive use and grower dependence on copper-based pesticides has raised concerns about environmental contamination and the development of resistance in pathogens (9, 16, 17). In bacteria, copper resistance genes improve copper export, intracellular copper ion sequestration, or enzymatic detoxification and can be acquired via plasmids, phages, and other mobile genetic elements (18–22). Occasionally, increased copper tolerance can also be achieved by overexpression of copper homeostasis genes, without need for horizontal acquisition of resistance genes (23). Although some studies have described certain copper-tolerance variability in *E. amylovora (*1, 24, 25), there is no direct evidence or reports of copper-resistant strains with new or additional copper resistance gene copies in the chromosome or in plasmids enhancing the pathogen resistance to copper.

In this work, we identified for the first time naturally occurring *E. amylovora* strains showing characteristic copper hypersensitivity, unable to grow on *E. amylovora* selective/differential media containing 1.5–2 mM CuSO_4_. While *E. amylovora* possesses chromosomally encoded copper homeostasis systems typical of gram-negative bacteria (14, 15, 26), the mechanisms underlying differential copper sensitivity in this pathogen are unknown. Additionally, previous work identified 44 regulated genes (mostly involved in membrane transport and stress response) during short-term copper exposure in a strain with regular copper tolerance (15). However, there is no information on the evolution of copper adaptation responses occurring during prolonged copper exposure, required for sustained survival and growth in the presence of copper excess. This knowledge is especially relevant in agricultural settings, where the pathogen may face fluctuating sublethal copper concentrations over extended periods.

This work represents the first identification and characterization of copper-hypersensitive isolates of *E. amylovora*. Through integrated phenotypic and transcriptomic analyses comparing copper-hypersensitive strains and strains with typical copper tolerance, we identified distinct copper response strategies in the fire blight pathogen. The copper-hypersensitive phenotype was associated with slower growth, increased susceptibility to paraquat and cadmium, and dysregulated copper-induced exopolysaccharide production. Transcriptomic profiling demonstrated that while both strain types mounted similar immediate responses to copper shock, hypersensitive isolates exhibited inefficient, resource-intensive responses to prolonged copper exposure. These results contrasted sharply with the efficient, targeted homeostasis mechanisms of a copper-tolerant strain. Results in this study advance our understanding of bacterial copper detoxification mechanisms and open new avenues for investigating metal homeostasis networks and their role in bacterial survival under environmental stress.

## RESULTS AND DISCUSSION

### Identification and characterization of copper sensitivity in *E. amylovora* strains

Growth on media containing sublethal copper concentrations (1.5–2 mM CuSO_4_, depending on the medium) gives *E. amylovora* colonies a characteristic color and morphology, which has been used for selective/differential media development (27–29).

**Fig 1:**
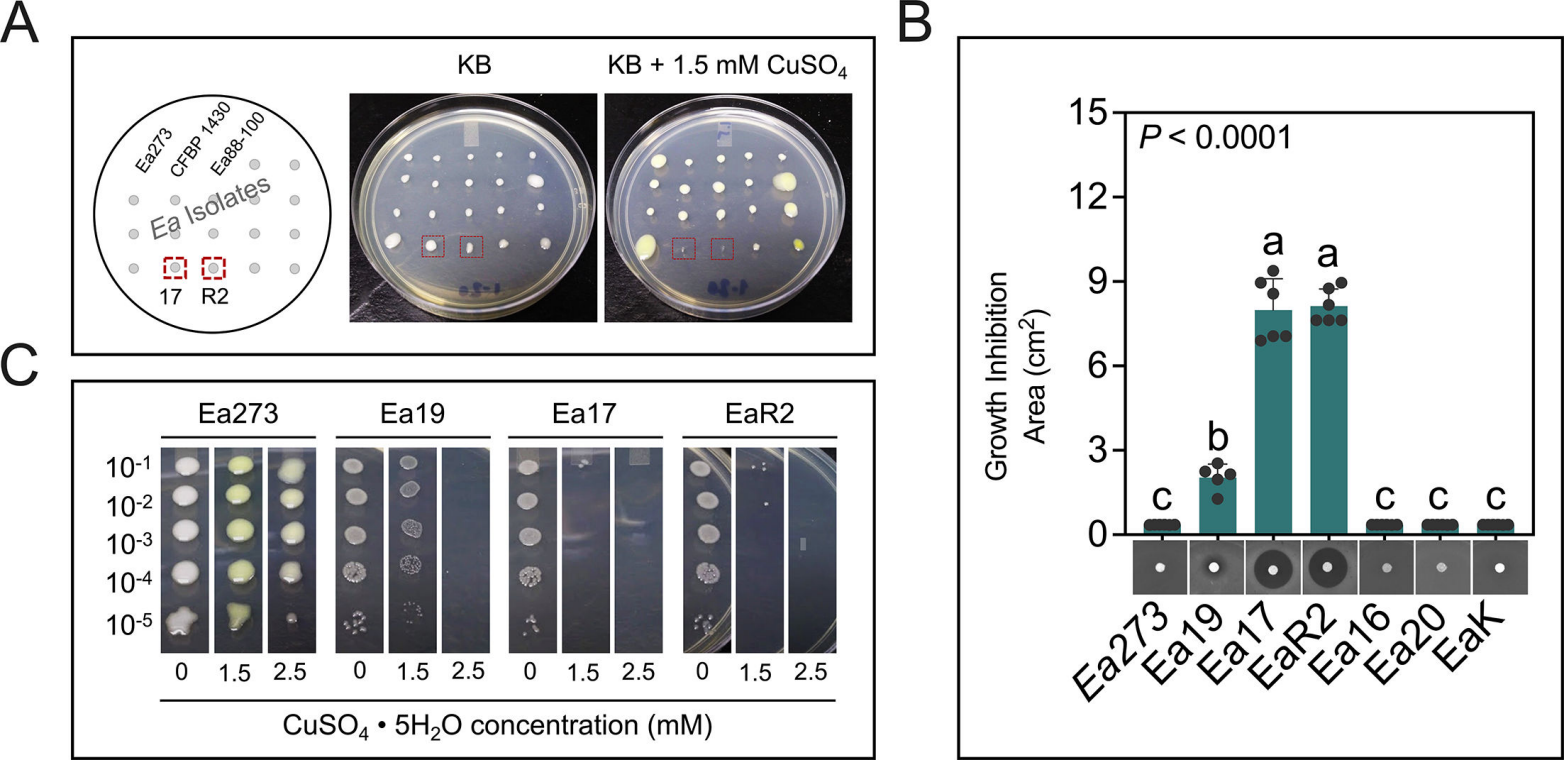
Detection and quantification of copper sensitivity in *E. amylovora* isolates. (**A**) Phenotypes of the copper-sensitive strains EaR2 and Ea17 in a stab agar assay on King’s B (KB) and KB plus 1.5 mM CuSO_4_. The scheme on the left indicates the position of two reference *E. amylovora* strains (Ea273 and CFBP 1430) and a strain used in other studies (Ea88-100), with typical copper tolerance, as well as different American *E. amylovora* isolates including EaR2 and Ea17. (**B**) Disk assay on minimal basal medium A amended with 0.05% nicotinic acid and 0.2% glucose (30, 31), showing no growth inhibition by 15 mM CuSO_4_ in the reference strain Ea273 and most isolates (Ea16, Ea20, and EaK), strong copper sensitivity of strains Ea17 and EaR2, and an intermediate response in Ea19. The *P* value on the top indicates statistical differences between the assayed groups by a Brown-Forsythe ANOVA test (F, 209.6; DFn, 3.0; DFd, 9.822). Different letters indicate significant differences between strains (α = 0.05) by Dunnett’s T3 multiple comparisons tests. (**C**) Characterization of copper susceptibility of Ea17, EaR2, and Ea19 by a spot dilution assay on KB amended with 0 to 2.5 mM CuSO_4_.

The copper-sensitive strain EaR2 was detected during isolation. While CCT agar plates inoculated with plant macerates yielded hundreds of typical dome-shaped *E. amylovora* colonies, RESC agar (with 1.5 mM CuSO_4_) produced disproportionately fewer pale-yellow, highly mucoid *E. amylovora* colonies. Copper sensitivity in EaR2 and other isolates was tested by stabbing overnight cultures on KB and RESC agar media. Most strains grew normally, but EaR2 and another isolate, Ea17, failed to grow on the copper-containing medium (Fig. 1A).

In disk assays, Ea273 and other isolates with standard copper tolerance (Ea16, Ea20, and EaK) showed no inhibition zones around disks loaded with 15 mM CuSO_4_. In contrast, Ea19 produced intermediate-sized inhibition halos, while the copper-hypersensitive strains EaR2 and Ea17 produced the largest halos (Fig. 1B). Similarly, in drop-dilution assays, Ea273 grew on KB with up to 2.5 mM CuSO_4_, Ea19 grew at 1.5 mM but failed at 2.5 mM, and the growth of Ea17 and EaR2 was inhibited at 1.5 mM CuSO_4_ (Fig. 1C). These findings were further validated in liquid medium assays, with copper MIC values in MGY broth: Ea273, 0.64 mM; Ea19, 0.51 mM; Ea17, 0.21 mM; and EaR2, 0.20 mM.

### Differential growth responses to copper exposure in *E. amylovora* strains

**Fig 2:**
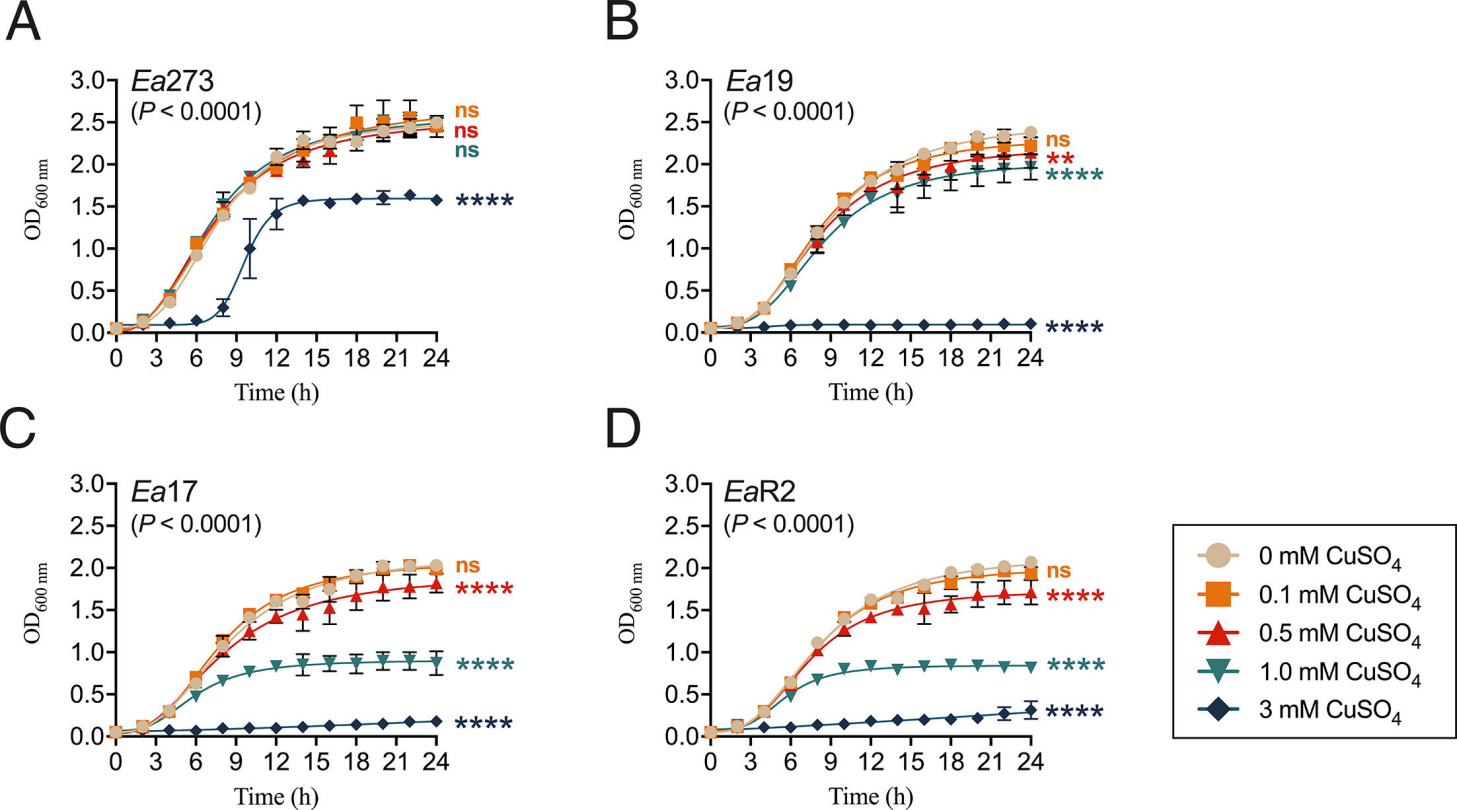
Effect of increasing copper concentrations in *E. amylovora*’s growth in LB. (**A–D**) Growth curves were carried out at 28°C in LB amended with increasing CuSO_4_ concentrations ranging from 0 to 3 mM, with shaking at 180 rpm and A_600_ nm measurements performed every 2 h for 24 h. Points show average values of two experiments performed in triplicate. *P* values in each chart show the statistical significance of differences between the AUCs at each copper concentration, assessed by one-way ANOVA (α = 0.05). Orange, red, turquoise, and blue symbols at the end of the curves denote nonsignificant (ns) or significant differences (**, *P* < 0.01; ****, *P* < 0.0001) between the AUCs in LB without copper and LB with 0.1, 0.5, 1.0, and 3.0 mM CuSO_4_, assessed by Dunnett’s multiple comparison tests.

Growth responses to copper were further characterized in LB with 0–3 mM CuSO_4_ (Fig. 2, Table 1). In the copper-tolerant strain Ea273, growth curves revealed similar areas under the curve (AUCs) at 0–1.0 mM CuSO_4_ (*P* ≥ 0.6603), with maximum A_600_ nm around 2.5 (Table 1). At 3 mM CuSO_4_, despite reduced maximum A_600_ nm (1.635) and extended lag phase, exponential growth showed the shortest doubling time (1.15 h) (Fig. 2A, Table 1).

**TABLE 1:** Parameters associated with *E. amylovora* growth in LB amended with copper

Strain	Copper conc. (mM)	Time to mid-log	Doubling time (h)	Max. absorbance (A_600_ nm)
Ea273	0	7 h 06 min	1.42	2.4950
	0.1	6 h 29 min	1.35	2.5550
	0.5	6 h 1 min	1.34	2.4650
	1	6 h 16 min	1.35	2.5200
	3	9 h 38 min	1.15	1.6350
Ea19	0	7 h 44 min	1.57	2.3800
	0.1	7 h 20 min	1.53	2.2350
	0.5	7 h 19 min	1.55	2.1400
	1	7 h 47 min	1.71	1.9650
	3	NA^***a***^	NA	0.1040
Ea17	0	7 h 15 min	1.62	2.0300
	0.1	7 h 03 min	1.57	2.0300
	0.5	6 h 33 min	1.60	1.8200
	1	5 h 34 min	1.62	0.8950
	3	NA	12.48	0.1790
EaR2	0	7 h 17 min	1.62	2.0700
	0.1	7 h 26 min	1.61	1.9650
	0.5	6 h 53 min	1.65	1.7150
	1	5 h 27 min	1.59	0.8500
	3	NA	14.05	0.3150

^*a*^ NA, not applicable; indicates cases where data did not conform to the regression model, preventing reliable calculation of parameters.

The intermediate-sensitive strain Ea19 grew similarly with and without 0.1 mM CuSO_4_ (*P* = 0.7955), with significant growth differences compared to LB only at 0.5–3 mM CuSO_4_ (*P* ≤ 0.0068). Cultures reached the mid-log phase around 25 min earlier with 0.1–0.5 mM CuSO_4_, with minimal effects on doubling times (Fig. 2B; Table 1). At 3 mM CuSO_4_, growth was severely impaired (doubling time 4.76 h; maximum A_600_ nm 0.104), barely doubling the initial A_600_ nm in 24 h (Fig. 2B; Table 1).

Copper hypersensitive strains Ea17 and EaR2 showed significant growth reduction at 0.5–3 mM CuSO_4_, most pronounced at 3 mM (*P* < 0.0001) (Fig. 2C and D). At 1 mM CuSO_4_, these strains behaved markedly differently from intermediate-sensitivity strain Ea19. Maximum A_600_ nm was reduced to 0.985 (Ea17) and 0.850 (EaR2), compared to 1.965 in Ea19 (Fig. 2B through D, Table 1). Both copper hypersensitive strains reached the mid-log phase up to 2 h earlier with increasing copper concentrations from 0.1 to 1 mM. At 3 mM, CuSO_4_ growth was minimal (final A_600_ nm 0.179–0.315), without a recognizable exponential phase. A rough estimation of the doubling time using growth values for 24 h revealed values over 12 h (Fig. 2C and D; Table 1).

Overall, these results indicate that, while Ea273 was able to cope with a wide range of copper concentrations and use this essential nutrient for growth, the other strains showed narrower ranges of copper tolerance, with earlier development of growth patterns indicative of copper reaching toxic levels within the cells.

### Copper-sensitive *E. amylovora* strains exhibit altered responses to cadmium and/or paraquat, and their virulence correlates with amylovoran secretion

**Fig 3:**
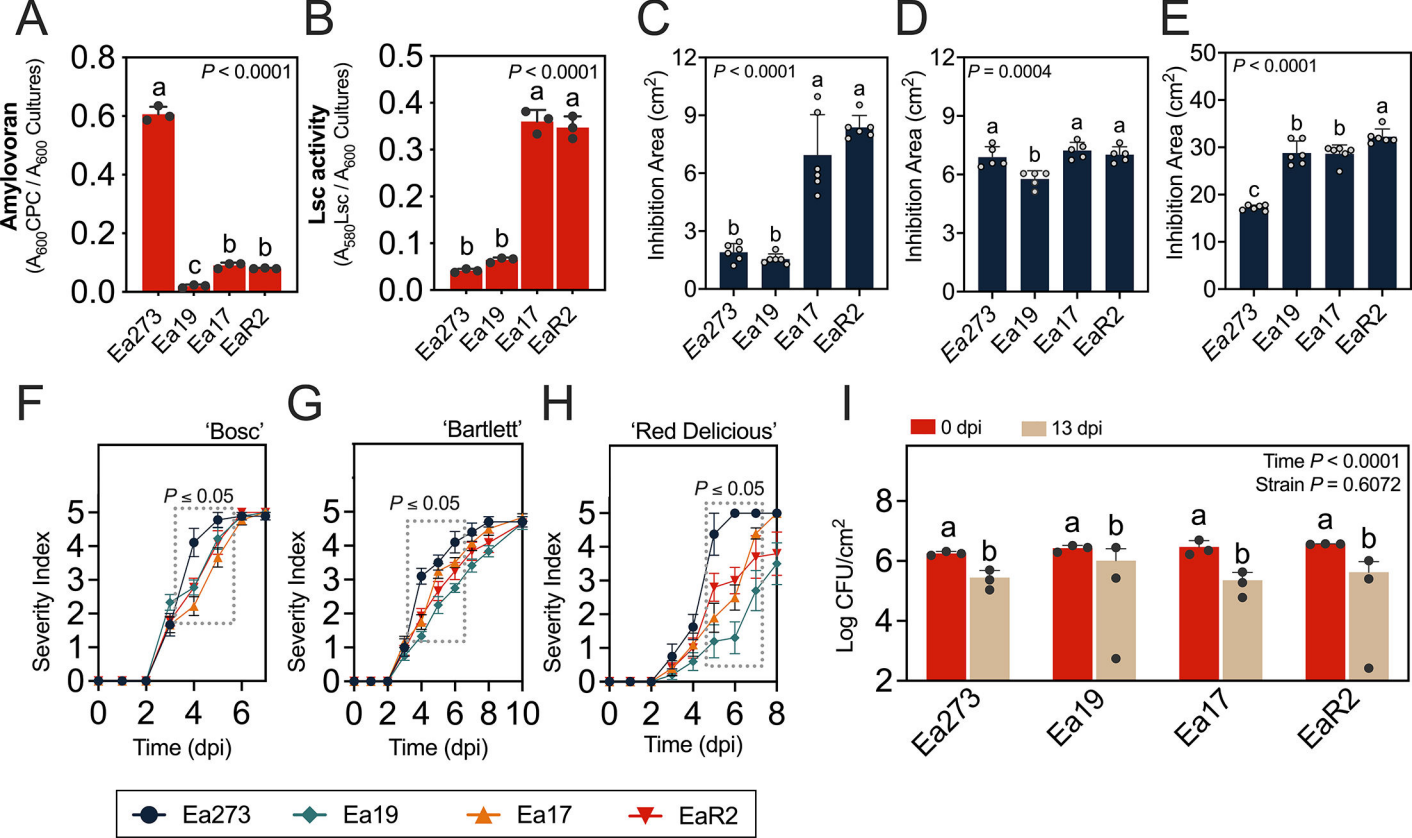
Phenotypical strain characterization. (**A**) Relative amylovoran and (**B**) levan production quantified by the CPC and levansucrase methods using cultures in MBMANic with 1% sorbitol and LB, respectively. (**C**) Susceptibility to 10 mM cadmium; (**D**) 1M hydrogen peroxide; (**E**) 50 mM paraquat assessed by a disk inhibition assay on MBMANic agar with 0.2% glucose. Virulence in detached, immature pears of cultivars (**F**) Bosc and (**G**) Bartlett and (**H**) apples of cv. Red Delicious. (**I**) Survival in the tobacco leaf apoplast at time points 0 and 13 days post-infiltration. Inocula for virulence assays in detached fruit and survival in tobacco leaves were prepared in LB. The *P* values in each chart indicate the significance of differences assessed by one-way or two-way ANOVA, depending on the assay. In charts F–H, the dotted rectangles indicate the time points with statistically significant differences between Ea273 and any of the copper-sensitive strains. In the remaining charts, different letters indicate significant differences assessed by one- or two-way ANOVA and multiple comparisons tests. The significance level in all cases was 5%.

Different phenotypic traits directly or indirectly linked to copper resistance (32–34) were compared between copper-tolerant Ea273 and the strains with intermediate (Ea19) or high (Ea17 and EaR2) copper sensitivity (Fig. 3). Strains Ea19, Ea17, and EaR2 produced less amylovoran than Ea273 (*P ≤* 0.0001), with Ea19 showing the lowest levels (Fig. 3A). Conversely, Ea17 and EaR2 produced 6.7-fold more levan than Ea273 and Ea19 (*P* < 0.0001) (Fig. 3B).

Although copper and cadmium export systems typically differ (35), they may also contribute to ROS detoxification (36). In stress response assays, Ea273 and Ea19 showed higher cadmium resistance than Ea17 and EaR2 (*P* < 0.0001) (Fig. 3C). While H_2_O_2_ responses showed no correlation with copper susceptibility, except for slightly higher resistance in Ea19 (*P* ≤ 0.0059) (Fig. 3D), all copper-sensitive strains displayed greater paraquat sensitivity than Ea273 (*P* < 0.0001), with EaR2 showing the highest inhibition (*P* ≤ 0.0162) (Fig. 3E). These results suggest a correlation between copper sensitivity and altered resistance to cadmium and intracellular superoxide caused by paraquat.

While functional copper homeostasis systems contribute to virulence in some plant pathogens (37, 38), our virulence assays revealed a different relationship between copper resistance and virulence in *E. amylovora*. The statistical analysis indicated significant effects of time (*P* < 0.0001) and strain (*P ≤* 0.05). However, the strain with intermediate copper tolerance Ea19 exhibited the lowest virulence overall (*P* ≤ 0.0411), followed by the copper-hypersensitive strains Ea17 and EaR2, and the copper-tolerant strain Ea273, which showed the highest virulence. These differences were most pronounced in “Red Delicious” apple fruitlets versus “Bosc” and “Bartlett” pear fruitlets (Fig. 3F through H). The virulence patterns of Ea273, Ea19, Ea17, and EaR2 strongly correlated with amylovoran production levels (Fig. 3A), consistent with the well-established association between virulence and amylovoran secretion in *E. amylovora* (39).

Survival in tobacco leaves showed time effects (F (1, 16) = 42.20, *P* < 0.0001) but not strain effects (F (3, 16) = 0.6283, *P* = 0.6072), indicating no links between copper sensitivity and survival in non-compatible hosts (Fig. 3I).

### Copper-driven EPS production patterns in *E. amylovora* vary in copper-hypersensitive strains

**Fig 4:**
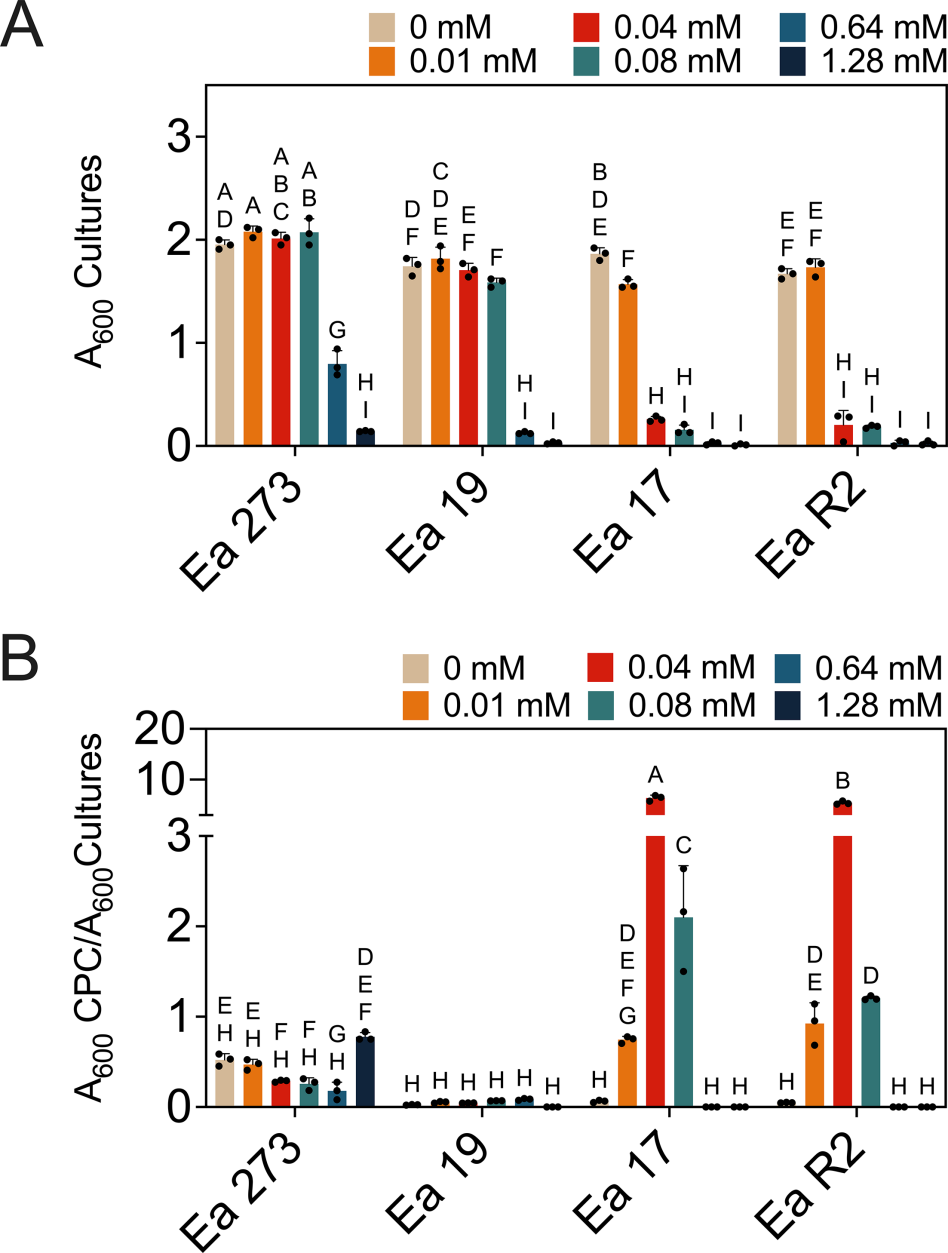
Effect of increasing copper concentrations in amylovoran production in MBMA with 1% sorbitol. (**A**) The effect of copper on *E. amylovora* growth. The copper concentrations were chosen based on results from preliminary assays. (**B**) The supernatants of these overnight cultures were used for amylovoran quantification by the CPC method. Amylovoran production is expressed relative to bacterial growth. Results show average values of three technical replicates. A two-way ANOVA with mean comparisons using Tukey’s tests at a significance level of 5% was used to test for statistically significant differences, which are indicated with different letters.

**Fig 5:**
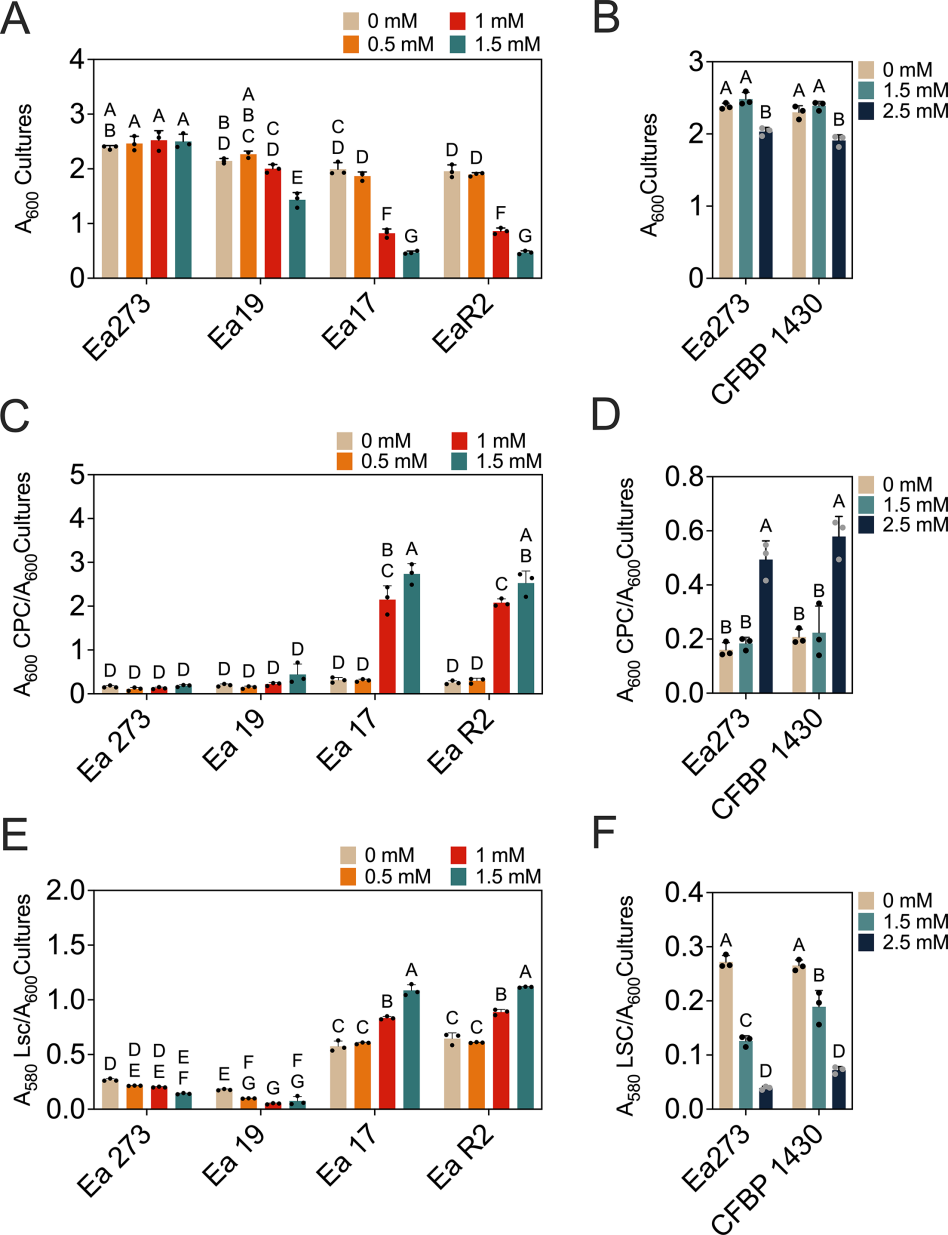
Effect of copper in *E. amylovora* growth and exopolysaccharide production in LB. Cells were grown overnight in LB amended with different copper concentrations. In a first assay, strains Ea273, Ea19, Ea17, and EaR2 were grown with (**A**) 0–1.5 mM CuSO_4_ and supernatants were used for relative quantification of both (**C**) amylovoran and (**E**) levan. In a second assay to confirm the trends observed in the first assay with Ea273, the effects of higher copper concentrations on (**B**) growth; (**D**) amylovoran; and (**F**) levan synthesis were assessed again in Ea273 and another reference strain with typical copper tolerance, CFBP 1430. Results show average values of three technical replicates. Different letters indicate statistically significant differences according to two-way ANOVA with Tukey’s multiple comparisons tests (α = 0.05).

*E. amylovora* develops mucoid colonies on copper-containing media, which is probably related to induction of EPS secretion by copper (27, 28). Our results confirmed amylovoran induction by copper in a concentration-dependent and strain-dependent manner (Fig. 4 and 5). Copper-sensitive strains Ea17 and EaR2 showed extreme amylovoran overproduction (110-fold increase) at 0.01–0.08 mM CuSO_4_ (*P* < 0.0001), peaking at 0.04 mM, while Ea19 did not modify amylovoran levels in response to copper (*P* > 0.9999). In Ea273, amylovoran levels decreased when grown with 0.01–0.64 mM CuSO_4._ but exposure to 1.28 mM caused a significant induction in comparison to 0.64 mM CuSO4 (*P* = 0.0393) (Fig. 4B). Peak amylovoran secretion was associated with high growth repression by copper (Fig. 4A).

In LB medium, the effects of copper on amylovoran production were similar as in MBMANic (Fig. 5A through D). In Ea17 and EaR2, increasing copper concentrations led to amylovoran overproduction up to 8.7–9.9-fold at 1.5 mM CuSO_4_ (*P* < 0.0001). Ea19 did not show changes in amylovoran production regardless of the assayed copper concentration (*P* ≥ 0.4180) (Fig. 5C). In Ea273, increasing copper concentrations had an effect only at 2.5 mM CuSO_4_, which caused a 3.1-fold increase in amylovoran levels (*P* = 0.0002) (Fig. 5C and D). This response was reproduced in the European *E. amylovora* strain CFBP 1430 with similar copper tolerance levels as Ea273 (*P* < 0.0001) (Fig. 5D).

Regarding levan production in LB, increasing copper concentrations to 1.5–2.5 mM repressed levan synthesis 7- and 3.7-fold in Ea273 and CFBP 1430, respectively (*P* < 0.0001) (Fig. 5D and F). Ea19 behaved similarly, with significant reduction in levan secretion at copper concentrations from 0.5 mM on and peaking at 1.5 mM CuSO_4_ (2.3-fold reduction, *P* = 0.0023) (Fig. 5E). Conversely, copper-hypersensitive strains Ea17 and EaR2 increased levan production (1.4 to 1.8-fold) at 1–1.5 mM CuSO_4_ (*P* < 0.0001).

Enhanced EPS secretion induced by copper is a common response across different organisms (40–42). In a previous study, authors demonstrated measurable copper-binding capacity of *E. amylovora* EPSs in a mineral salt medium, yet this protective effect was incomplete, with EPSs delaying but not preventing copper toxicity (32). However, it is possible that in field conditions, the viscous matrix of bacterial ooze within and on plant tissues offers a more robust barrier against copper than dissolved EPSs in solution. Under our experimental conditions, the intense and earlier copper-induced EPS response in hypersensitive *E. amylovora* strains likely represents a compensatory mechanism rather than an effective protection strategy, though it could confer a survival advantage in agricultural environments. Moreover, the contrasting levan production patterns between copper-hypersensitive and copper-tolerant strains also suggest differences in copper sensing or stress response regulation. In hypersensitive strains, a lower threshold for copper toxicity might trigger levan overproduction as a maladaptive reaction, less effective than the more measured response seen in tolerant strains like Ea273 to fight copper toxicity.

### Copper preadaptation fails to protect *E. amylovora* against copper shock and has no effect on virulence

**Fig 6:**
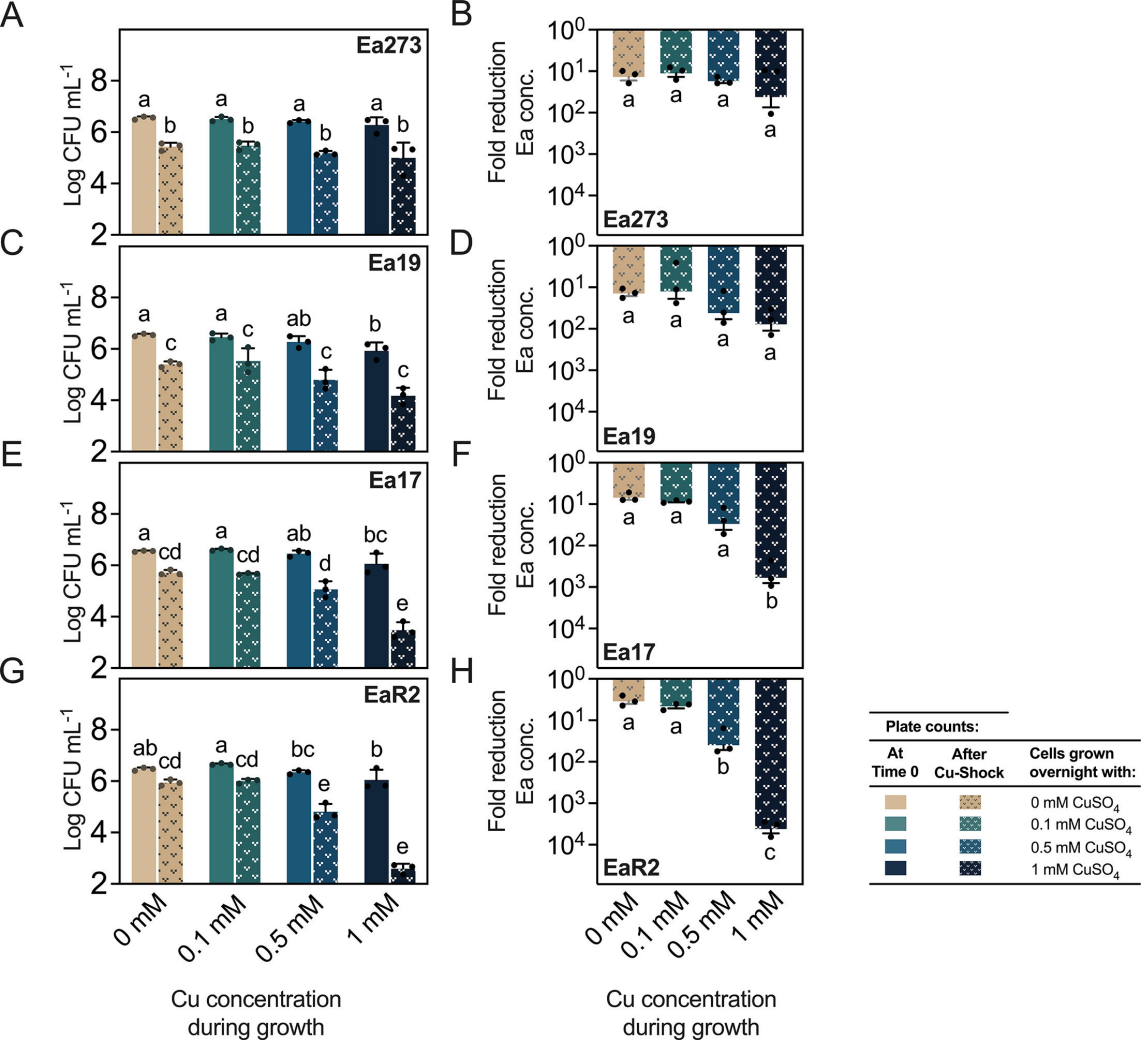
Effect of copper pre-exposure in copper-shock survival. *E. amylovora* cultures of strains Ea273 (**A and B**), Ea19 (**C and D**), Ea17 (**E and F**), and EaR2 (**G and H**) were grown overnight in LB and LB amended with sublethal concentrations of 0.1, 0.5, and 1 mM CuSO_4_. Cell suspensions adjusted to 10^6^ CFU mL^−1^ were copper-shocked with 4 mM CuSO_4_ for 5 min. (**A, C, E and G**) Raw cell concentrations before (solid colors) and after copper-shock treatment (pattern). (**B, D, F and H**) Survival fold-reduction of *E. amylovora* concentrations after copper shock with respect to time 0. Data are average values of three technical repeats. Error bars show the SD. Different letters indicate significant differences between the assayed data groups assessed by Tukey’s multiple comparisons tests (α = 0.05).

Pre-exposure to sublethal concentrations of toxic compounds in bacteria usually leads to protection against higher concentrations of the same compound or other stresses. To test if this was true for *E. amylovora* and copper, Ea273, Ea19, Ea17, and EaR2 overnight cultures in LB plus 0–1 mM CuSO_4_ were rinsed and exposed to copper shock with 4 mM CuSO_4_ in sterile saline. Both exposure to increasing copper concentrations during growth (ANOVA, *P ≤* 0.0447) and copper-shock treatment (ANOVA, *P* < 0.0001) negatively impacted bacterial survival after copper shock, with these effects being more pronounced in copper-hypersensitive strains EaR2 and Ea17, followed by Ea19 and Ea273 (Fig. 6A, C, E and G).

Charts on the right in Fig. 6 show the survival fold reduction after copper shock in copper-adapted cells and non-adapted cells. In agreement with the analysis of raw data, results revealed no significant differences between treatments in Ea273 (*P* ≥ 0.6605) (Fig. 6B) and Ea19 (*P* ≥ 0.1706) (Fig. 6D). However, copper pre-exposure during growth enhanced death rates in Ea17 up to 2.6 log units (*P* < 0.0001) and in EaR2 up to 3.6 log units (*P* < 0.0001) (Fig. 6E and G).

In other bacterial models, pre-exposure to sublethal copper concentrations such as the one used in this study, 0.1 mM, improved survival outcomes to copper stress (43, 44). Under our experimental conditions, even the lowest copper concentration tested (0.1 mM) did not enhance *E. amylovora* survival after copper shock compared to untreated cells. Growth with higher copper concentrations (≥ 0.5 mM) further reduced *E. amylovora* post-shock survival, particularly in copper-hypersensitive strains (Ea17 and EaR2). This contrasts with canonical adaptive responses in other bacteria (43, 44) and suggests species-specific differences in copper response mechanisms and/or experimental conditions in our study hindering the expected protective effect of copper pre-exposure. Our results might be associated with efflux pump saturation, glutathione depletion, and/or dysregulation of copper-binding proteins during overnight growth with copper excess, leaving cells vulnerable to higher copper doses.

While copper pre-exposure enhances virulence in some human pathogens (45, 46), pre-exposure of *E. amylovora* to 0.5 mM CuSO_4_ during growth had no significant effect on virulence in any of the tested strains (*P* > 0.9897) (Fig. S1). These results suggest that *E. amylovora* can quickly recover critical virulence functions after copper stress, deploying pathogenicity and virulence factors as effectively as non-stressed cells. The lack of virulence changes in copper-exposed cells also indicates that copper stress responses and virulence mechanisms are likely uncoupled in *E. amylovora*.

### Copper induces distinct transcriptional responses in copper*-*tolerant and copper-hypersensitive *E. amylovora* strains

**Fig 7:**
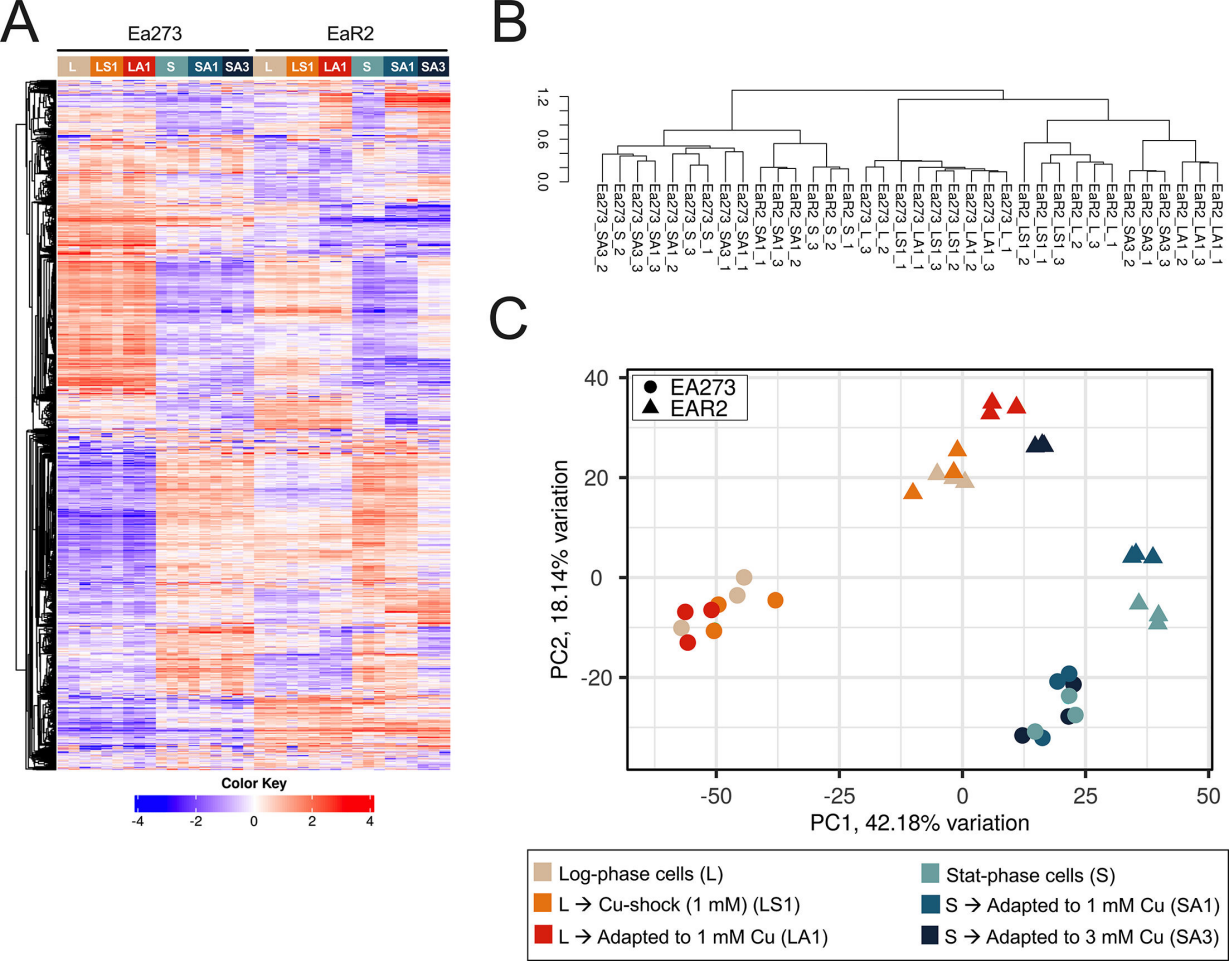
Transcriptomic analysis of Ea273 and EaR2 strains under different copper treatments. Heatmap showing the expression profiles of the top 2,000 differentially expressed genes across all samples, which are organized by experimental conditions: L, mid-log phase cells: beige; LS1, mid-log phase cells copper-shocked with 1 mM CuSO_4_: orange; LA1, cells grown with 1 mM CuSO_4_ until reaching the mid-log phase: red; S, stationary-phase cells: pale blue; SA1, cells grown for 20 h in LB amended with 1 mM CuSO_4_: teal; SA3, cells grown for 20 h in LB amended with 3 mM CuSO_4_: dark blue. The color scale indicates normalized expression values. (**A**) Hierarchical clustering dendrogram of samples, generated using Pearson correlation coefficient as the distance metric and (**B**) average linkage method. (**C**) Principal component analysis (PCA) plot showing the separation of samples based on the first two principal components. Samples are colored by treatment as in the heatmap and shaped by strain (Ea273: circles, EaR2: triangles).

RNA-Seq analysis revealed strain-specific transcriptional responses to copper shock and copper adaptation in *E. amylovora*. The heatmap (Fig. 7) indicated different gene expression patterns across treatments and strains. In treatments with log-phase cells, Ea273 showed less distinct gene expression changes between control log-phase cells (L), copper-shocked cells (LS1), and copper-adapted cells (LA1) compared to EaR2. The copper-hypersensitive strain EaR2 exhibited more pronounced expression shifts, especially between control (L) and copper-adapted cells (LA1). Both strains, particularly EaR2, showed differential expression patterns between control stationary-phase cells (S) and cells adapted to grow with 1 mM (SA1) and 3 mM (SA3) CuSO_4_ (Fig. 7A).

Hierarchical clustering (Fig. 7B) showed clear separation of EaR2 samples by treatment, with replicates mostly clustering together, indicating good experimental reproducibility. EaR2 displayed distinct clustering by growth phase and copper treatment, while Ea273 exhibited less defined transcriptional changes, particularly between copper-treated and control samples. Within treatments, samples clustered well by strain, highlighting strain-specific copper responses.

PCA (Fig. 7C) confirmed more subtle transcriptional responses to copper stress in Ea273 than in EaR2. PC1 (42.18% variation) correlated significantly with the effect of copper treatments (*P* = 7.82 × 10^−5^), while PC2 (18.4%) correlated with strain effects (*P* = 5.26 × 10^−8^). The different clustering of Ea273 and EaR2 within each treatment suggests strain-specific responses to copper stress, especially in stationary-phase cells grown with 3 mM CuSO_4_. PC3 and PC4 also correlated with treatment (*P* = 0.0106 and *P* = 0.0138), indicating copper responses extended beyond PC1.

### Basal transcriptomic profiles of non-stressed log-phase and stationary-phase *E. amylovora* cultures

**Fig 8:**
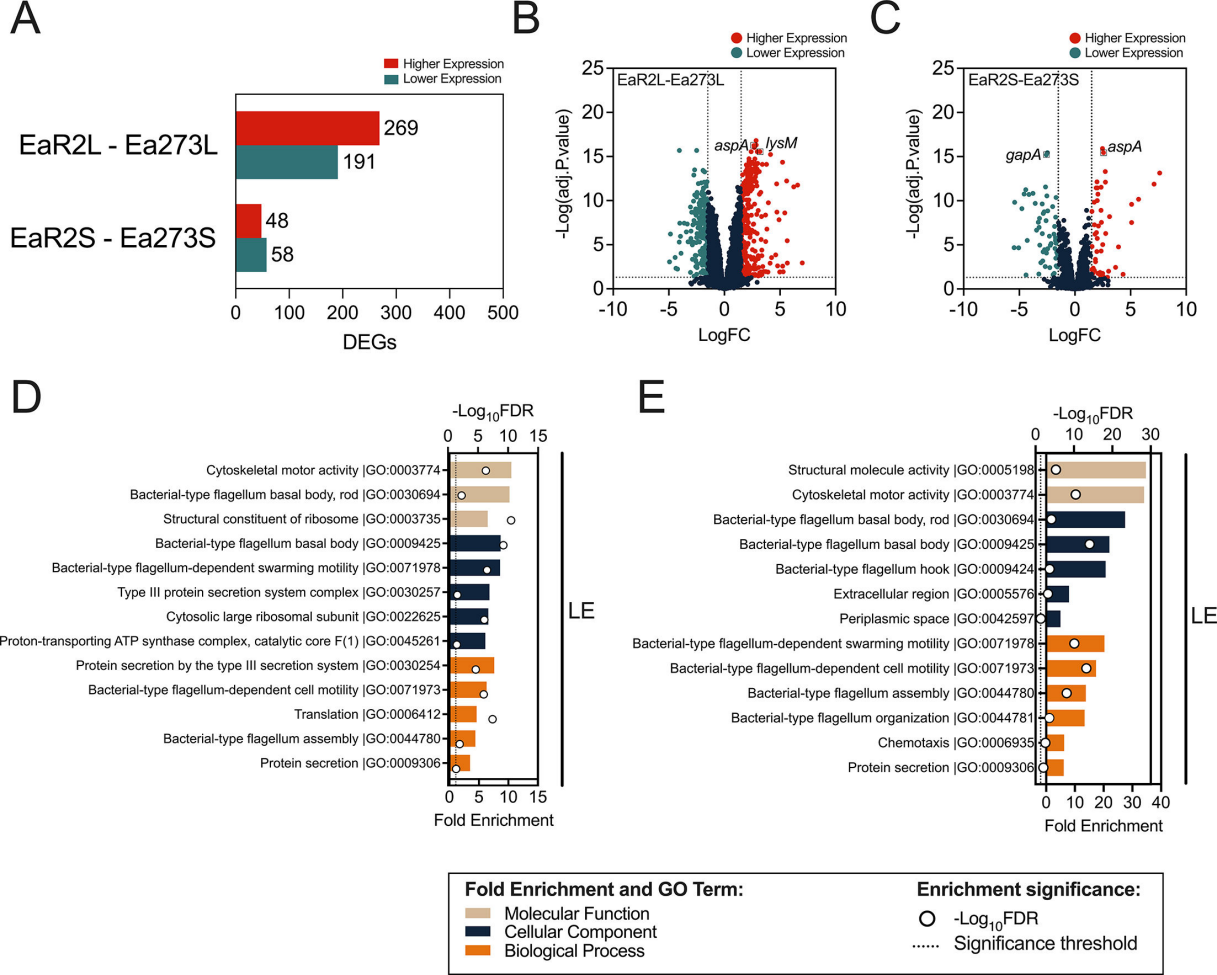
Comprehensive analysis of differentially expressed genes (DEGs) in EaR2 compared to Ea273. (**A**) Bar plot showing the number of DEGs in mid-log (EaR2L-Ea273L) and stationary-phase cells (EaR2S-Ea273S), with higher and lower expressed genes in EaR2 compared to Ea273 indicated. (**B and C**) Volcano plots for EaR2L vs. Ea273L (**B**) and EaR2S vs. Ea273S (**C**), highlighting DEGs with functional gene names in NCBI, with the highest and lowest expression levels in EaR2 relative to Ea273. Only genes with a functional gene symbol within the top 10 most expressed DEGs are shown in the plots. (**D and E**) GO term enrichment analysis for DEGs in log-phase (**D**) and stationary-phase cells (**E**). Bars represent fold enrichment (bottom x-axis) for GO terms categorized into molecular function (beige), cellular component (blue), and biological process (orange). White dots indicate statistical significance of the enrichment, expressed as -log_10_FDR (top x-axis), with a dashed line marking the significance threshold (FDR ≤ 0.05, i.e., -log_10_FDR ≥ 1.30). GO enrichment analysis revealed terms enriched only in genes with lower expression (LE) in EaR2 compared to Ea273, in both log-phase and stationary-phase cells.

Transcriptome analysis revealed distinct expression patterns between EaR2 and Ea273, with more differentially expressed genes (DEGs) in the log phase than stationary phase (Fig. 8A through C).

GO term enrichment analysis showed lower expression in log-phase EaR2 cells of genes linked to protein secretion (particularly, associated to the T3SS), motility, translation, and energy production (Fig. 8D). The T3SS is a pathogenicity factor in *E. amylovora* (47)*,* so a significantly lower expression of these genes correlated well with EaR2’s lower virulence. Similarly, lower expression of translation and energy production pathways aligned with EaR2’s slower growth in LB.

In stationary-phase cells, EaR2 showed lower expression than Ea273 of genes involved in environmental sensing and adaptation (chemotaxis and motility) and genes linked to periplasmic and extracellular regions (Fig. 8E). Although cells were just grown to the stationary phase, the lowered expression of structural molecular activity genes in EaR2 compared to Ea273 suggests that EaR2 prioritized survival over cellular maintenance during the stationary phase.

Two lists with the DEGs in log-phase and stationary-phase of EaR2 cells in comparison to Ea273 are provided as supplemental material (Tables S1 and S2).

### Core *E. amylovora* transcriptomic responses to copper stress

**Fig 9:**
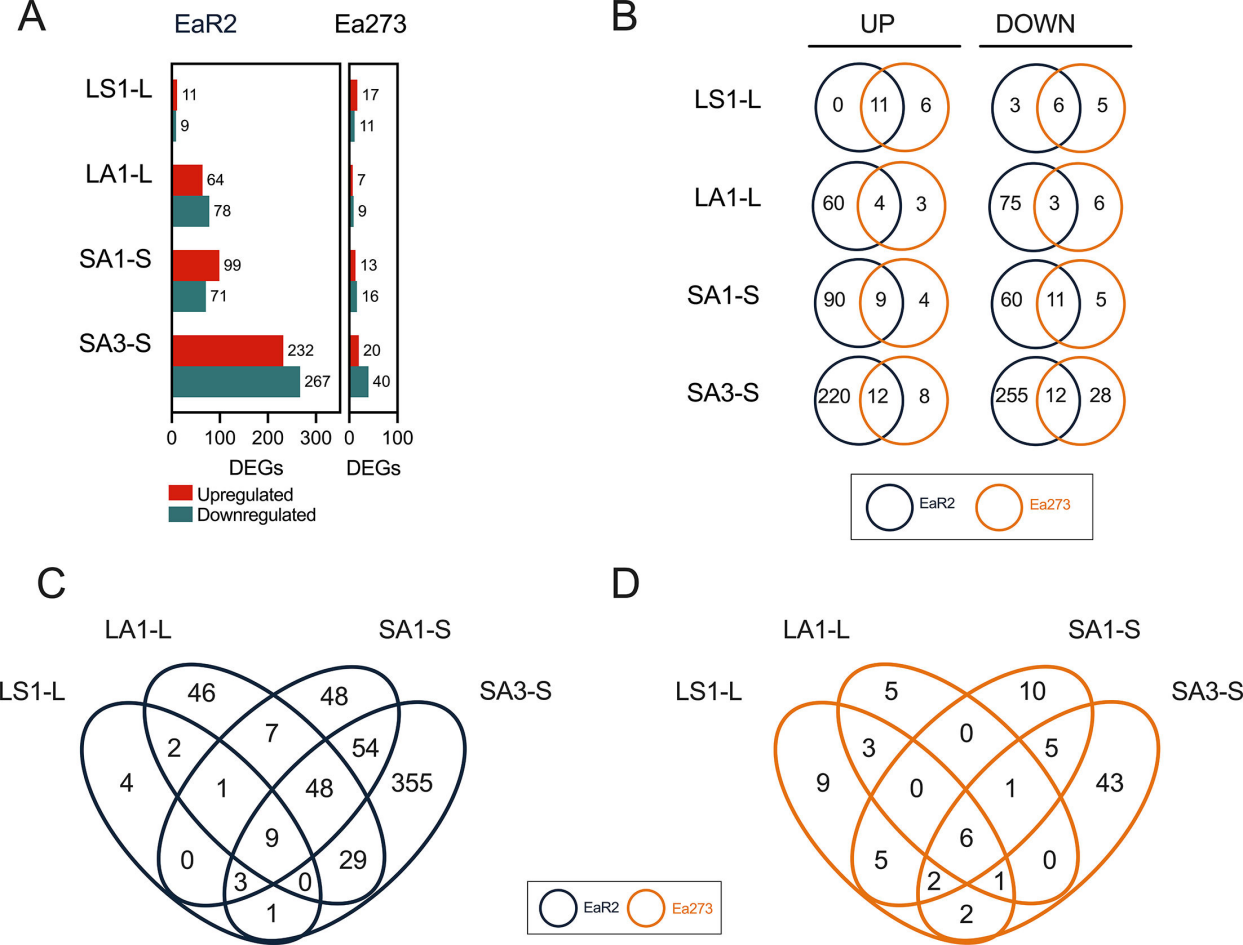
Comparative analysis of differentially expressed genes (DEGs) in EaR2 and Ea273 strains under different types of copper stress. (**A**) Number of upregulated and downregulated genes in Ea273 and EaR2 in response to four different treatments with copper: LS1, log-phase cells copper-shocked with 1 mM CuSO_4_; LA1, log-phase cells adapted to 1 mM CuSO_4_; SA1 and SA3, stationary-phase cells adapted to 1 mM (SA1) and 3 mM CuSO_4_ (SA3), respectively. In each case, the DEG number was calculated with respect to the control log-phase (L) and stationary-phase (S) cells grown in LB without copper. (**B**) Venn diagrams showing differences and similarities in the number of DEGs in the two *E. amylovora* strains in response to the same treatments. (**C and D**) Venn diagrams illustrating common and specific DEGs in EaR2 (**C**) and Ea273 (**D**) in response to different copper shock and copper adaptation treatments.

There were DEGs regulated similarly regardless of the copper treatment assayed (core copper responses) (Fig. 9). A list of the DEGs regulated regardless of the copper treatment in Ea273 and EaR2 is detailed in Table 2.

**TABLE 2:** *E. amylovora* Ea273 and EaR2 core responses to copper

Strain	Gene ID^*b*,*c*^	Symbol^*c*^	Gene function^*c*^	Average expression (Log_2_FC)^*a*^			
				(LS1-L)	(LA1-L)	(SA1-S)	(SA3-S)
Ea273	**8911929**	*cueO*	Multicopper oxidase CueO	5.54	5.25	4.90	5.02
	**8914287**	*copA*	Copper-exporting P-type ATPase CopA	5.46	4.74	5.07	4.72
	8914722	*EAMY_RS33475*	Zinc/cadmium/mercury/lead-transporting ATPase	3.29	1.55	1.98	2.15
	**8914283**	*EAMY_RS22290*	TraB/GumN family protein	3.24	2.51	3.57	2.50
	**8914242**	*hpt*	Hypoxanthine phosphoribosyl transferase	−1.90	−1.57	−2.53	−2.31
	8912016	*EAMY_RS31370*	PepSY-associated TM helix domain-containing protein	−2.57	−4.11	1.55	2.18
EaR2	**8911929**	*cueO*	Multicopper oxidase CueO	5.55	5.17	5.29	5.39
	**8914287**	*copA*	Copper-exporting P-type ATPase CopA	5.01	4.04	5.08	4.68
	23673387	*EAMY_RS28935*	DUF1471 domain-containing protein	4.02	5.54	2.36	1.81
	**8914283**	*EAMY_RS22290*	TraB/GumN family protein	3.32	2.51	3.38	2.78
	8913976	*soxS*	Superoxide response transcriptional regulator SoxS	2.23	2.37	1.76	2.02
	8912479	*spy*	ATP-independent periplasmic protein-refolding chaperone Spy	2.02	6.23	6.36	6.97
	8914442	*EAMY_RS19660*	YlaC family protein	−1.60	−1.62	−2.00	−1.75
	8913092	*EAMY_RS30310*	Cystathionine gamma-synthase family protein	−1.97	−1.74	−2.16	−3.05
	**8914242**	*hpt*	Hypoxanthine phosphoribosyl transferase	−2.84	−2.71	−3.08	−4.71

^*a*^ (LS1-L) 5-min copper-shock treatment with 1 mM CuSO4 on mid-log phase cells; (LA1-L) cells grown with 1 mM CuSO4 up to the mid-log phase; (SA1-S) cells grown with 1 mM CuSO4 up to the stationary phase; (SA3-S) cells grown with 3 mM CuSO4 up to the stationary phase.

^*b*^ Gene IDs in bold letters indicate DEGs commonly regulated in Ea273 and EaR2.

^*c*^ Gene IDs, locus tags, symbols, and functions obtained from the DAVID database for *E. amylovora* CFBP 1430.

In Ea273, copper exposure triggered the differential expression of six genes (Fig. 9D): *copA, cueO, EAMY_RS22290, EAMY_RS33475, EAMY_31370,* and *hpt*. Most of these genes were upregulated. Genes *copA* and *cueO* are essential components of the copper homeostasis system, encoding a P_1B_-type ATPase that extrudes copper to the periplasm, and a multi-copper oxidase that oxidizes Cu(I) to less toxic ion Cu(II) (14), respectively. *EAMY_RS22290,* located downstream of *copA,* is likely part of the same operon or regulatory network, potentially involved in copper detoxification or stress response. *EAMY_RS33475* encodes another P_1B_-type ATPase (sometimes annotated as ZntA) contributing to zinc, cadmium, lead, and mercury homeostasis. Although it is not a copper transporter, ZntA is induced by copper, and its deletion leads to increased ROS and copper sensitivity in other bacteria (36, 48). *EAMY_RS31370* encodes a PepSY-associated protein indirectly related to iron transmembrane transport (49, 50). This gene was downregulated in log-phase cells but upregulated in stationary-phase cells grown with copper (Table 2). Excess copper can displace iron from iron-sulfur clusters in metalloproteins, leading to ROS generation (10, 14), so *EAMY_RS31370* may help maintain proper metal homeostasis during copper exposure. The differential regulation in log- and stationary-phase cells may be indicative of different iron imbalances and/or different iron needs in each physiological state. Finally, the consistent downregulation of *hpt,* encoding hypoxanthine phosphoribosyl transferase (HPT), may reflect a shift in metabolic priorities toward survival in the presence of copper. Hypoxanthine phosphoribosyltransferase (HTP) is a phosphoribosyl diphosphate (PRPP)-dependent enzyme, critical for purine salvage (51). Our observation that *hpt* is downregulated under copper stress aligns with findings in *Staphylococcus aureus,* where copper causes a decrease in PRPP levels by inhibiting phosphoribosylpyrophosphate synthetase (Prs) (52). Mutants accumulating intracellular PRPP develop increased copper tolerance, while *hpt* overexpression causes impaired growth in the presence of copper (52). In *E. amylovora,* however, copper-induced *hpt* repression occurred without *prs* repression (adjusted *P* values > 0.05 regardless of the tested stress). This indicates potential PRPP flux redirection toward NAD+/NADP +biosynthesis for DNA repair and sustain redox balances (51, 53).

Repression of *hpt* may be linked to a (p)ppGpp-mediated stringent response. Although we detected no transcriptional changes in *relA* and *spoT*–in charge of (p)ppGpp biosynthesis and hydrolysis—RelA activation can occur post-translationally via ribosomal stalling (54). Future studies quantifying intracellular PRPP, purine pools, and (p)ppGpp levels could clarify whether copper stress in *E. amylovora* prioritizes NAD+/NADP+ production, stringent signaling, or both.

EaR2 core copper responses involved regulation of nine DEGs in response to all the copper treatments (Fig. 9C), with four of them coinciding with Ea273: *copA, cueO, EAMY_RS22290,* and *hpt* (Table 2). Additional upregulated DEGs in *EaR2* included *EAMY_RS28935*, *soxS*, and *spy*, which are mostly associated with stress responses, indicating a higher effort by EaR2 in maintaining cellular homeostasis under copper stress (55–59).

The downregulation of *EAMY_RS19660* and *EAMY_RS30310* is probably linked to reducing side effects of copper exposure. *EAMY_RS19660* encodes a YlaC family protein associated with protein secretion and resistance to boric acid (60, 61). EAMY_RS30310 is a cystathionine gamma-synthase. These enzymes participate in the metabolism of sulfur-containing amino acids, generating H_2_S as a byproduct. H_2_S negatively impacts enzymatic functions and reacts with copper, generating toxic precipitates and/or disrupting copper homeostasis (62, 63).

### Differential gene expression during copper shock

**Fig 10:**
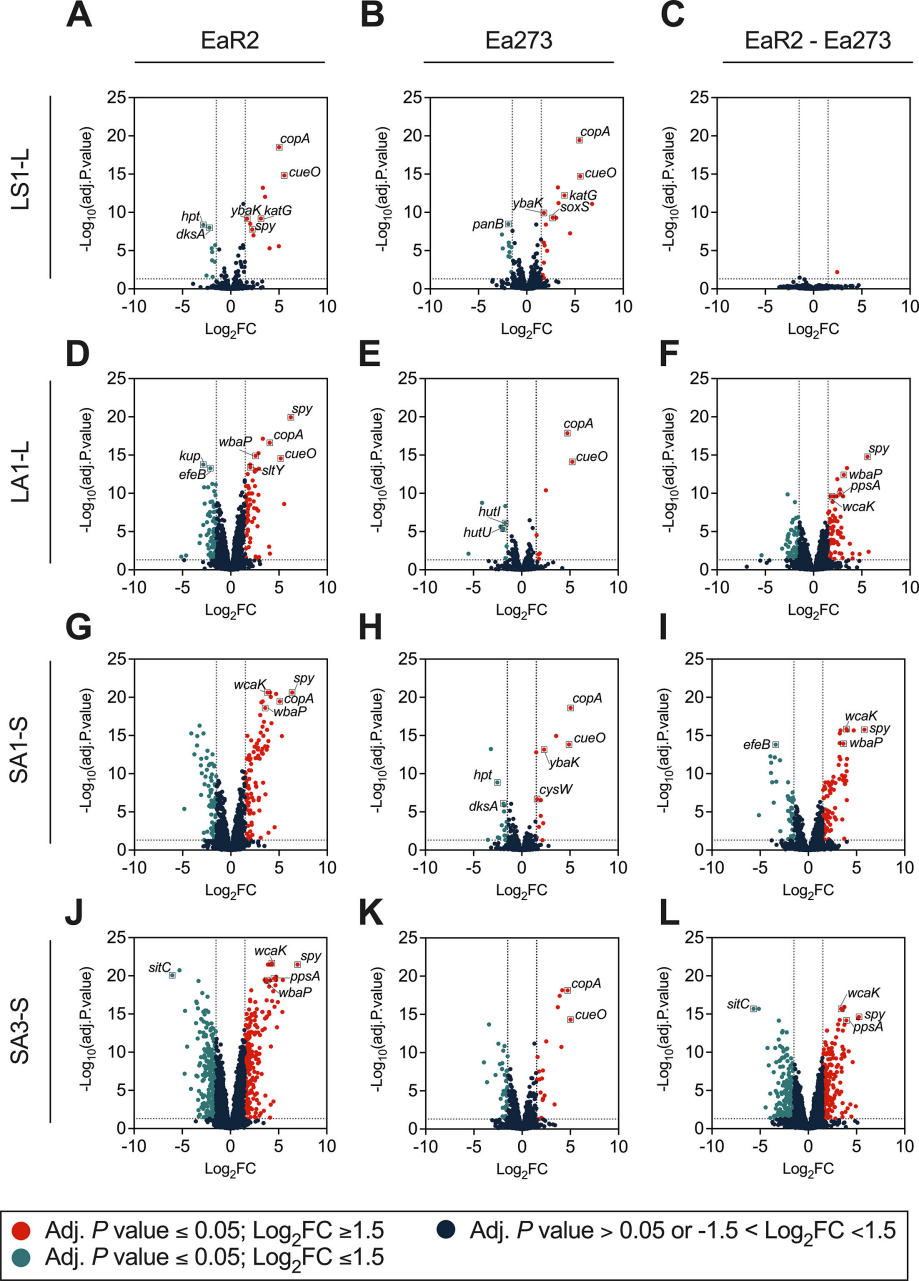
Volcano plots of differentially expressed genes (DEGs) in EaR2 and Ea273 strains under different copper stress conditions. (**A–C**) LS1-L contrast between the responses of mid-log phase cells exposed to 1 mM CuSO_4_ for 5 min, i.e., copper-shock conditions (LS1) and untreated mid-log phase cells (**L**); (**D–F**) LA1-L contrast between the mid-log phase, copper-adapted cells, i.e., grown to the mid-log phase in the presence of 1 mM CuSO_4_ (LA1), and cells grown similarly without copper (**L**); (**G–I**) SA1-S contrast between copper-adapted cells grown in the medium amended with 1 mM CuSO_4_ to the stationary phase (SA1) and cells grown similarly without copper (S); (**J–L**) SA3-S, same contrast as the latter, but cells were grown in LB amended with 3 mM CuSO_4_. Left- and mid-column charts illustrate the transcriptomic responses of EaR2 and Ea273, respectively, and the right column charts contrast the responses of EaR2 versus Ea273. Red and turquoise dots represent significant DEGs (adjusted *P* values ≤ 0.05) with fold-change expression values above 1.5 or below −1.5. Dark blue dots represent genes showing nonsignificant expression changes (adjusted *P* value > 0.05), or fold-change values between −1.5 and 1.5. For simplicity, only genes with a functional gene symbol within the top 10 most expressed DEGs are shown in the plots.

The differential gene expression analysis during copper shock (LS1-L) showed nearly identical responses between strains EaR2 and Ea273 (Fig. 9 and 10A through C). A complete list of DEGs in Ea273 and EaR2 in response to copper shock is provided as supplemental material (Tables S3 and S4). Differences in EaR2 and Ea273 copper-shock responses are summarized in (Table 3). The copper-shock response activated copper and oxidative stress-related genes while downregulating energy metabolism genes. This response was similar in both strains, although the enrichment analysis was only significant for Ea273 (Fig. 10A).

**TABLE 3:** Common DEGs in EaR2 and Ea273 in response to different copper treatments

Treatment	Gene ID^*a*^	Gene symbol	Gene function	Ea273		EaR2	
				Log2FC	FDR	Log2FC	FDR
Copper shock (LS1-L)	8912441	*EAMY_RS17985*	Glutathione peroxidase	6.77	7.73E-12	4.98	1.30E-08
	8911929	*cueO*	Multicopper oxidase CueO	5.54	1.85E-15	5.55	9.28E-19
	8914287	*copA*	Copper-exporting P-type ATPase CopA	5.46	3.62E-20	5.01	9.72E-23
	23673387	*EAMY_RS28935*	DUF1471 domain-containing protein	4.49	5.50E-08	4.02	3.09E-08
	8912322	*katG*	Catalase/peroxidase HPI	3.90	6.16E-13	3.13	1.23E-12
	8914722	*EAMY_RS33475*	Zinc/cadmium/mercury/lead-transporting ATPase	3.29	6.14E-12	3.56	1.20E-15
	8914283	*EAMY_RS22290*	TraB/GumN family protein	3.24	5.48E-14	3.32	5.90E-17
	8911634	*EAMY_RS25705*	Helix-turn-helix domain-containing protein	3.05	5.09E-10	2.35	3.93E-10
	8913976	*soxS*	Superoxide response transcriptional regulator SoxS	2.71	4.94E-10	2.23	6.23E-11
	8912479	*spy*	ATP-independent periplasmic protein-refolding chaperone Spy	1.98	3.94E-09	2.02	8.20E-12
	8911440	*ybaK*	Cys-tRNA(Pro)/Cys-tRNA(Cys) deacylase YbaK	1.77	1.18E-10	1.69	1.54E-12
	8913363	*hrpB*	ATP-dependent helicase HrpB	−1.59	3.64E-06	−1.92	1.03E-07
	8913536	*panD*	Aspartate 1-decarboxylase	−1.60	6.13E-03	−1.76	1.69E-02
	8914241	*pcnB*	Polynucleotide adenylyltransferase PcnB	−1.75	6.56E-05	−1.88	4.12E-04
	8911927	*mrcB*	Bifunctional glycosyl transferase/transpeptidase	−1.86	9.11E-07	−1.64	2.36E-06
	8914242	*hpt*	Hypoxanthine phosphoribosyltransferase	−1.90	5.50E-05	−2.84	1.33E-11
	8912892	*panB*	3-methyl-2-oxobutanoate hydroxymethyltransferase	−1.91	3.47E-09	−1.94	1.39E-06
	8912703	*gluQRS*	tRNA glutamyl-Q (35) synthetase GluQRS	−2.48	5.15E-06	−2.52	2.34E-04
Copper adaptation (LA1-L)	8911929	*cueO*	Multicopper oxidase CueO	5.25	7.47E-15	5.17	2.73E-15
	8914287	*copA*	Copper-exporting P-type ATPase CopA	4.74	1.41E-18	4.04	2.48E-17
	8914283	*EAMY_RS22290*	TraB/GumN family protein	2.51	4.11E-11	2.51	1.16E-11
	**23673387** ^***a***^	***EAMY_RS28935***	**DUF1471 domain-containing protein**	**1.79**	**3.65E-02**	**5.54**	**2.48E-09**
	8914242	*hpt*	Hypoxanthine phosphoribosyltransferase	−1.57	8.21E-04	−2.71	2.23E-09
	8914442	*EAMY_RS19660*	YlaC family protein	−1.69	4.71E-09	−1.62	2.80E-07
	**8912016**	***EAMY_RS31370***	**PepSY-associated TM helix domain-containing protein**	**−4.11**	**1.74E-09**	**−1.54**	**1.56E-03**
Copper adaptation (SA1-S)	8914287	*copA*	Copper-exporting P-type ATPase CopA	5.07	2.41E-19	5.08	3.52E-20
	8914283	*EAMY_RS22290*	Trab/Gumn family protein	3.57	1.13E-15	3.38	1.10E-15
	8911929	*cueO*	Multicopper oxidase CueO	4.90	1.52E-14	5.29	1.20E-15
	8914442	*EAMY_RS19660*	YlaC Family Protein	−3.21	5.83E-14	−2.00	1.34E-08
	8911440	*ybaK*	Cys-tRNA(Pro)/Cys-tRNA(Cys) deacylase YbaK	2.30	7.02E-14	2.03	1.07E-12
	8913538	*EAMY_RS30290*	ABC transporter permease	1.50	1.56E-13	1.72	1.10E-15
	8914242	*hpt*	Hypoxanthine phosphoribosyltransferase	−2.53	1.39E-09	−3.08	1.13E-12
	8912636	*cysW*	Sulfate/thiosulfate ABC transporter permease CysW	1.59	2.19E-07	2.10	4.22E-08
	8913940	*dksA*	RNA polymerase-binding protein DksA	−1.89	8.36E-07	−2.11	1.50E-08
	8913363	*hrpB*	ATP-dependent helicase HrpB	−1.84	1.45E-06	−2.00	3.17E-07
	8913092	*EAMY_RS30310*	Cystathionine gamma-synthase family protein	−1.84	8.68E-06	−2.16	1.25E-06
	8914070	*tauB*	Taurine ABC transporter ATP-binding subunit	2.22	3.02E-04	1.93	2.76E-02
	23673399	*can*	Carbonate dehydratase	−2.08	5.63E-04	−1.80	1.37E-03
	8912641	*cysD*	Sulfate adenylyltransferase subunit CysD	1.78	9.20E-04	2.97	6.58E-06
	8914861	*EAMY_RS25825*	Hypothetical protein	−1.58	1.03E-03	−1.88	4.69E-07
	**8913504**	***EAMY_RS19950***	**Hrp pili protein HrpA**	**−1.51**	**1.23E-02**	**−2.06**	**1.46E-03**
	8914241	*pcnB*	Polynucleotide adenylyltransferase PcnB	−1.79	1.23E-02	2.01	3.50E-03
	8913570	*EAMY_RS17820*	Sulfate ABC transporter substrate-binding protein	1.50	2.03E-02	2.15	2.49E-04
	8914246	*sfsA*	DNA/RNA nuclease SfsA	−2.48	2.20E-02	−2.80	5.45E-03
	8913104	*thpR*	RNA 2',3'-cyclic phosphodiesterase	−1.71	4.50E-02	−1.81	1.28E-02
	8913535	*panC*	Pantoate--beta-alanine ligase	−1.70	4.55E-02	−1.74	3.18E-02
Copper adaptation (SA3-S)	8912732	*EAMY_RS20195*	Peroxiredoxin	4.15	6.91E-19	4.74	1.31E-20
	8914287	*copA*	Copper-exporting P-type ATPase CopA	4.72	7.29E-19	4.68	1.75E-19
	8913942	*EAMY_RS20190*	Thioredoxin family protein	3.89	3.78E-18	4.75	3.64E-20
	8914651	*EAMY_RS20185*	Sigma-70 family RNA polymerase sigma factor	3.70	1.16E-16	3.76	1.62E-17
	8911929	*cueO*	Multicopper oxidase CueO	5.02	4.90E-15	5.39	5.45E-16
	**8914442**	***EAMY_RS19660***	**YlaC family protein**	**−3.43**	**2.09E-14**	**−1.75**	**4.35E-09**
	8914283	*EAMY_RS22290*	TraB/GumN family protein	2.50	3.37E-12	2.78	5.56E-14
	8914013	*EAMY_RS25670*	FAD-binding protein	−1.82	1.38E-11	−2.04	9.16E-14
	**8911634**	***EAMY_RS25705***	**Helix-turn-helix domain-containing protein**	**4.07**	**1.81E-11**	**2.48**	**3.66E-08**
	8911440	*ybaK*	Cys-tRNA(Pro)/Cys-tRNA(Cys) deacylase YbaK	1.60	3.84E-10	1.67	3.64E-11
	**8914242**	***hpt***	**Hypoxanthine phosphoribosyltransferase**	**−2.31**	**3.17E-09**	**−4.71**	**3.92E-16**
	**8912554**	***motB***	**Flagellar motor protein MotB**	**−2.08**	**1.36E-08**	**−2.23**	**2.91E-11**
	**8912479**	***spy***	**ATP-independent periplasmic protein-refolding chaperone Spy**	**1.74**	**3.01E-08**	**6.97**	**3.35E-22**
	8911612	*EAMY_RS25565*	Hypothetical protein	−1.81	8.11E-08	−2.54	8.75E-13
	**8911546**	***rcsA***	**Transcriptional regulator RcsA**	**2.06**	**2.43E-07**	**3.57**	**8.52E-14**
	8912847	*fliD*	Flagellar filament capping protein FliD	−1.66	4.73E-07	−2.40	8.83E-08
	8912151	*metA*	Homoserine O-succinyltransferase	−1.66	5.24E-07	−1.54	2.30E-08
	30317028	*EAMY_RS35565*	ogr/Delta-like zinc finger family protein	−3.64	7.48E-07	−2.20	2.56E-09
	8914507	*EAMY_RS24385*	M15 family metallopeptidase	−1.56	1.80E-05	−2.78	1.00E-14
	23673387	*EAMY_RS28935*	DUF1471 domain-containing protein	2.33	4.13E-05	1.81	1.62E-04
	8912735	*EAMY_RS20035*	LysR family transcriptional regulator	−1.60	4.45E-05	−2.17	1.22E-10
	8911644	*EAMY_RS25785*	Acyltransferase	−1.56	6.94E-04	−1.66	4.63E-06
	8912203	*EAMY_RS33785*	DUF1471 domain-containing protein	2.00	3.45E-02	1.69	4.76E-02

^*a*^ Bold Gene IDs indicate common DEGs in the two strains, differentially regulated in EaR2 with respect to Ea273 (FDR < 0.05; |Log2FC| ≥ 1.5).

One of copper’s side effects is oxidative damage via copper-catalyzed ROS through Fenton-like reactions (10, 14). Enhanced ROS neutralizing activity improves resistance to both copper and other compounds causing ROS-derived damage (64). Beyond core copper responses (Table 2), copper shock strongly upregulated genes linked with oxidative stress (Table 3) like *EAMY_RS17985* and *katG* encoding glutathione peroxidase and catalase/peroxidase HPI, respectively. Additional upregulated DEGs in both strains were *soxS* and *spy,* regulating superoxide radical responses (59) and involved in ATP-independent protein folding (58). The zinc transporter ZntA (*EAMY_RS33475*) contributes to zinc and oxidative stress protection in other bacteria (36, 65), while *ybaK* ensures proper protein synthesis under environmental stress conditions through aminoacyl-tRNA editing (66).

Common downregulated DEGs in copper-shocked EaR2 and Ea273 included *mrcB, panB, pcnB, gluQRS, hrpB,* and *hpt* (Table 3)*,* which encode proteins involved in cell wall formation, vitamin synthesis, RNA stability, protein synthesis, virulence, and nucleic acid metabolism (67–72). Downregulation of these genes aligns with sudden growth rate reduction and metabolic adjustments under copper shock conditions.

The comparison of transcriptomic responses between strains revealed only two significant DEGs between EaR2 and EA273 exposed to copper shock: *EAMY_RS31370* and *trxC* (Table 4). *EAMY_RS31370,* linked to siderophore-dependent iron assimilation (49, 50), showed 2.45 Log_2_FC units higher expression in EaR2, suggesting differing iron homeostasis responses with Ea273 after short copper exposure. Gene *trxC,* encoding a thioredoxin important for H_2_O_2_ neutralization and other varied cellular processes (73), showed 1.45 Log_2_FC unit lower expression in EaR2. This potentially indicates reduced ROS management capacity in EaR2, consistent with its paraquat susceptibility (Fig. 3E).

**TABLE 4:** Top 10 DEGs between EaR2 and Ea273 during different copper treatments

Comparison	Gene ID	Gene symbol/locus tag	Function	EaR2	Ea273	EaR2 -Ea273	FDR
LS1-L	8912016	*EAMY_RS31370*	PepSY-associated TM helix domain-containing protein	−0.13	−2.57	2.45	6.42E-03
	8914591	*trxC*	Thioredoxin TrxC	0.32	1.77	−1.45	3.34E-02
							
LA1-L	8914036	*sucD*	Succinate-CoA ligase subunit alpha	0.19	−5.51	5.71	4.33E-03
	8912479	*spy*	ATP-independent periplasmic protein-refolding chaperone Spy	6.23	0.64	5.59	1.70E-15
	8913001	*rpe*	Ribulose-phosphate 3-epimerase	−4.62	0.77	−5.39	1.23E-02
	30316913	*rprA*	ncRNA	1.36	−3.41	4.76	8.73E-03
	8914295	*ybbP*	Putative ABC transporter permease subunit YbbP	3.97	−0.07	4.04	3.22E-02
	55585552	*EAMY_RS36320*	Hypothetical protein	1.31	−2.72	4.03	7.47E-03
	23673387	*EAMY_RS28935*	DUF1471 domain-containing protein	5.54	1.79	3.76	3.92E-04
	8912357	*EAMY_RS31520*	SulP family inorganic anion transporter	3.33	−0.14	3.46	5.03E-14
	8913660	*proW*	Glycine betaine/L-proline ABC transporter permease ProW	−3.23	0.03	−3.25	1.70E-03
	8912412	*wbaP*	Undecaprenyl-phosphate galactose phosphotransferase WbaP	2.57	−0.57	3.14	3.93E-13
							
SA1-S	8912479	*spy*	ATP-independent periplasmic protein-refolding chaperone Spy	6.36	0.55	5.82	1.76E-16
	8913269	*EAMY_RS33720*	Siderophore-interacting protein	−4.83	0.30	−5.13	2.69E-05
	8913418	*EAMY_RS24365*	Glycine zipper 2TM domain-containing protein	4.70	0.00	4.70	2.12E-16
	8912357	*EAMY_RS31520*	SulP family inorganic anion transporter	4.14	0.10	4.04	2.12E-16
	8914055	*EAMY_RS34145*	OmpA family lipoprotein	4.22	0.21	4.01	1.16E-12
	69102764	*EAMY_RS36505*	DUF5993 family protein	3.35	−0.64	3.99	2.85E-07
	30316917	*EAMY_RS35010*	Glycine zipper 2TM domain-containing protein	3.90	−0.08	3.98	4.75E-11
	8912414	*EAMY_RS27680*	Phage tailspike protein	4.07	0.09	3.97	1.63E-16
	8912415	*wcaK*	Colanic acid biosynthesis pyruvyl transferase WcaK	3.80	−0.15	3.95	1.48E-16
	8912409	*EAMY_RS27690*	Glycosyltransferase family 4 protein	3.80	−0.15	3.94	8.51E-10
							
SA3-S	8913950	*sitC*	Iron/manganese ABC transporter permease subunit SitC	−6.04	−0.36	−5.68	2.09E-16
	8912479	*spy*	ATP-independent periplasmic protein-refolding chaperone Spy	6.97	1.74	5.23	2.50E-15
	8914055	*EAMY_RS34145*	OmpA family lipoprotein	5.44	0.26	5.18	3.74E-15
	8911759	*EAMY_RS27275*	Metal ABC transporter substrate-binding protein	−5.29	−0.16	−5.14	2.09E-16
	8911933	*cas2e*	Type I-E CRISPR-associated endoribonuclease Cas2e	2.74	−2.19	4.93	1.20E-03
	69102764	*EAMY_RS36505*	DUF5993 family protein	4.83	0.23	4.61	1.97E-09
	30316913	*rprA*	ncRNA	4.47	0.01	4.47	2.63E-02
	8913450	*nuoE*	NADH-quinone oxidoreductase subunit NuoE	−1.06	3.37	−4.43	1.63E-03
	8913734	*dtd*	D-aminoacyl-tRNA deacylase	2.96	−1.40	4.36	6.59E-04
	8913134	*EAMY_RS23320*	HutD family protein	−3.40	0.84	−4.24	2.72E-11

The overlap of regulated DEGs (e.g., *copA, spy,* or *soxS*) between our study and prior work with a different copper-tolerant *E. amylovora* strain (15) confirms a conserved core set of copper-responsive genes that are consistently regulated in *E. amylovora* across different strains with varying copper sensitivity, under copper-shock conditions. However, despite this shared regulatory program, differences in copper sensitivity between Ea273 and EaR2 indicate that activation of classic copper-homeostasis genes alone is insufficient to guarantee survival under copper stress.

### Differential gene expression during copper adaptation in log-phase cells

EaR2 and Ea273 log phase cells exhibited nearly identical transcriptomic responses to copper shock, but their adaptation strategies during prolonged copper exposure diverged dramatically (Fig. 9; Fig. 10D through F; 11B and E; Table 4; Tables S3, S4 and S6). While copper adaptation in Ea273 involved fewer DEGs (16) compared to copper shock (28), EaR2 showed a more intense response with 142 DEGs during copper adaptation versus 20 during copper shock (Fig. 9).

**Fig 11:**
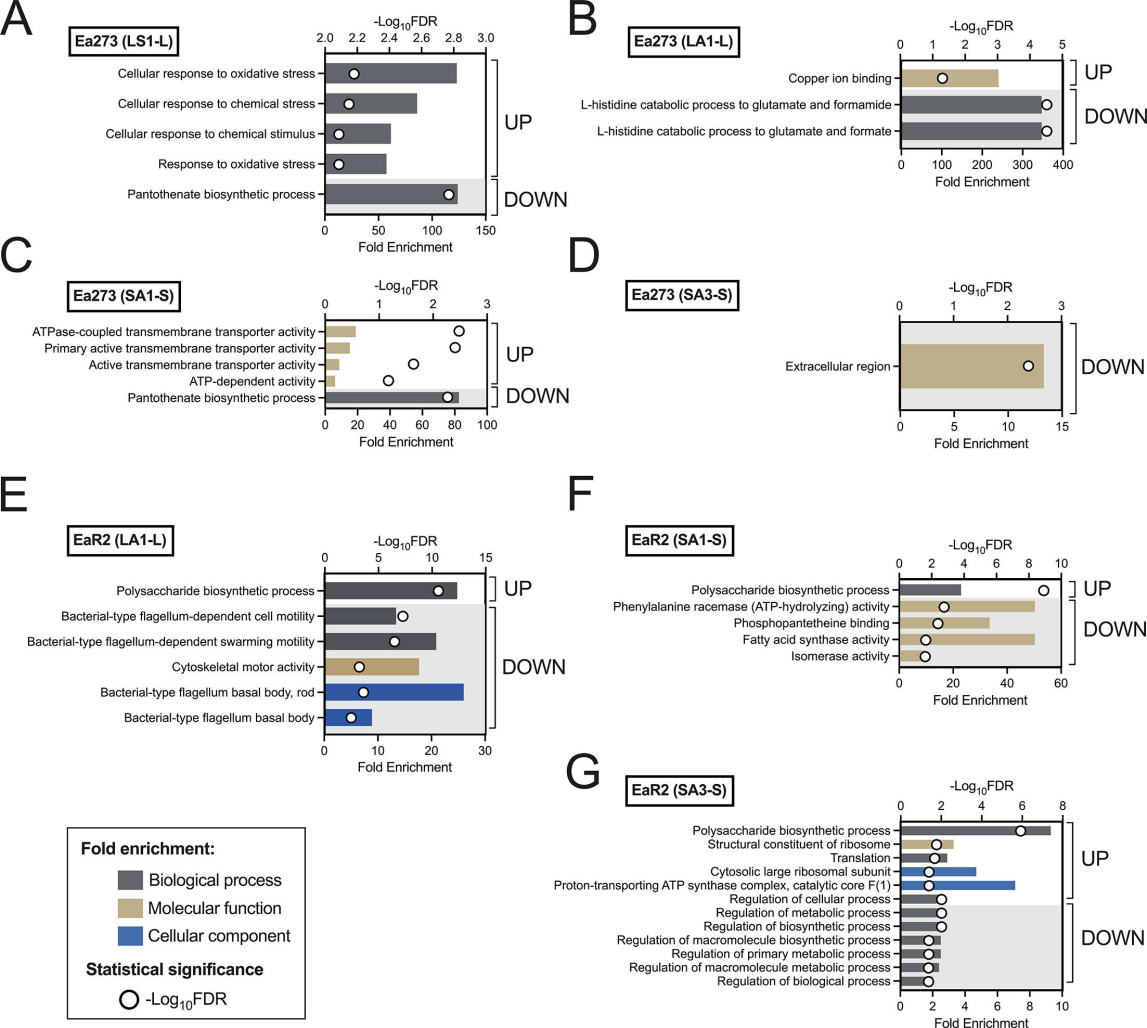
Differential Gene Ontology (GO) enrichment analysis of upregulated (UP) and downregulated (DOWN) genes in *E. amylovora* strains Ea273 (**A–D**) and EaR2 (**E–G**) under different copper treatments. The significance of enrichment is indicated by the -log10(FDR) values, represented by open circles. The fold enrichment is represented with bars. Only significant enriched GO terms are shown (FDR ≤ 0.05). The GO enrichment analysis was performed with DEGs resulting from comparisons between treatments and controls (e.g., LS1-L). Treatment abbreviations: LS1, copper-shock treatment with 1 mM CuSO_4_ for 5 min applied on log-phase cells; LA1, growth with 1 mM CuSO_4_ up to the mid-log phase; SA1, growth with 1 mM CuSO_4_ up to the stationary phase; SA3, growth with 3 mM CuSO_4_ up to the stationary phase. Control comparisons: -L, mid-log phase cells; -S, stationary-phase cells.

Common DEGs in copper-adapted EaR2 and Ea273 cells included core copper response genes (*copA, cueO, EAMY_RS22290,* and *hpt*) plus three additional DEGs: *EAMY_RS28935, EAMY_RS19660,* and *EAMY_RS31370* (Table 3). *EAMY_RS28935* and *EAMY_RS19660* were part of EaR2’s core copper responses but not Ea273’s (Table 2). This suggests different copper stress perception thresholds between these strains. *EAMY_RS31370,* potentially linked to iron transmembrane transport (49, 50) and part of Ea273’s core copper responses (Table 2), showed significantly lower expression in Ea273 than in EaR2 (Table 3), indicating the importance of iron homeostasis in Ea273 responses.

Despite these few common elements in EaR2 and Ea273 (Table 3), there were marked differences between strains. Ea273 showed a characteristic enrichment in downregulated genes linked to L-histidine breakdown (*hutI, hutH,* and *hutU)* (Fig. 11B; Fig. S2). L-histidine has metal chelating properties (74), and reduction of L-histidine catabolism probably leads to its accumulation, contributing to copper and oxidative stress protection (74–76).

In contrast, EaR2 exhibited significant enrichment in upregulated genes associated with polysaccharide biosynthesis and downregulated genes related to flagellar structure and motility (Fig. 11E; Fig. S3). This corroborated the copper-dependent EPS overproduction observed in this strain (Fig. 4 and 5).

Besides the different DEG profiles in copper-adapted Ea273 and EaR2 cells, there also were significant differences regarding the expression levels of multiple genes (Table 4; Table S6), including some of the commonly regulated genes in copper-adapted EaR2 and Ea273 cells (Table 3). Higher expression of the DEGs *EAMY_RS28935, spy, ybbP,* and *EAMY_RS31520* in EaR2 aligns with the idea of same copper treatments causing higher stress in EaR2 than in Ea273 (Tables 3 and 4). These genes encode membrane proteins and/or proteins responding to membrane damage (56, 77–79).

Other DEGs showed significantly lower expression in Ea273 than in EaR2, including *sucD, rprA,* and *EAMY_RS36320* (Table 4). Gene *sucD* encodes a succinyl-CoA synthetase. While the role of *sucD* in copper tolerance remains largely unexplored, it might be through indirect modulation of intracellular ROS levels. In *E. coli, sucD* mutants showed lower cytoplasmic H_2_O_2_ accumulation, correlating with enhanced resistance to toxic mammalian peptidoglycan recognition proteins (80 ). *rprA* is an Hfq-dependent small RNA regulating virulence and biofilm dispersal in *E. amylovora* (47), as well as general stress and envelope stress responses in other organisms (81). However, its exact contribution to copper responses has not been characterized. *EAMY_RS36320* encodes a hypothetical protein of unknown function.

Among the top 10 DEGs showing significantly lower expression in EaR2 versus Ea273, we identified *rpe* and *proW* (Table 4). *rpe* encodes a cytosolic metalloprotein using iron as cofactor (82). Its downregulation potentially limits copper-induced damage. Gene *proW,* encoding the inner membrane component of an ABC transport system, is typically activated during salt stress but downregulated during acid stress (83), suggesting its regulation might relate to pH imbalances during copper exposure.

### Differential gene expression during copper adaptation in stationary-phase cells

Transcriptomic analysis of stationary-phase cells grown with copper also revealed differential responses to copper adaptation between Ea273 and EaR2. Copper adaptation induced the regulation of more DEGs compared to the other treatments, with cells grown with 3 mM CuSO_4_ showing a larger number of DEGs than those in 1 mM CuSO_4_. Notably, EaR2 exhibited significantly more regulated DEGs than Ea273 (Fig. 9 and 10G through L; Tables S3, S4, S7 and S8).

In cells adapted to 1 mM CuSO_4_, apart from the core copper responses (Table 2), both strains upregulated common genes linked to sulfur metabolism and downregulated biosynthetic pathways and nucleic acid processing genes (Table 3, Fig. 11C, F; Fig. S2C and S3C). This pattern suggests a coordinated response to maintain copper homeostasis while creating an antioxidant environment via sulfur metabolism as well as reducing energy-intensive processes not essential for immediate survival under copper stress.

Specific Ea273 expression patterns grown with 1 mM CuSO_4_ involved more precise and robust responses, with significant upregulation of copper homeostasis genes and genes linked to sulfur metabolism (Fig. S2C and Table S3). Sulfur is essential for synthesizing antioxidant molecules like glutathione and other sulfur-rich copper chelators, contributing to protein repair, redox cycling, and support antioxidant mechanisms for copper detoxification (84, 85). Ea273 also downregulated DEGs related to pantothenate biosynthesis. Pantothenate is a precursor for coenzyme A, essential for energy metabolism and the tricarboxylic cycle (TCA). Limiting CoA synthesis in response to copper might help redirect energy and metabolites toward copper homeostasis and detoxification.

Specific EaR2 responses to growth with 1 mM CuSO_4_ shared similarities with copper adaptation in log-phase cells. There was an upregulation of genes linked to amylovoran biosynthesis and sulfur metabolism and a downregulation of genes associated with iron transport like *efeU* and *efeO,* among others (Fig. 11F; Fig. S3C and Table S4). The repression of iron uptake genes represents a conserved response to copper exposure, serving to counterbalance the increasing iron levels resulting from copper-mediated displacement (14).

Under higher copper stress (3 mM CuSO_4_), both strains upregulated genes linked to copper, envelope, oxidative, and other stress responses (e.g., *rcsA, spy, EAMY_RS20195,* and *EAMY_RS20190*) while downregulating motility genes (Fig. 11D and G; Fig. S2D and S3D; Table 3). This transcriptional pattern indicates that elevated copper concentrations enhance antioxidant defenses and other stress-related functions, while suppressing nonessential activities, particularly those associated with motility and specific metabolic pathways (Table 3).

Ea273 grown with 3 mM CuSO_4_ showed differential upregulation of genes involved in RNA gamma-capping, glutathione metabolism, and cytochrome complex assembly (Table S3 and Fig. S2D). This indicates a shift of metabolic resources toward copper detoxification, redox balance, and protection against oxidative damage, while reducing energy-demanding functions like swimming. EaR2 responses to the same treatment were characterized by a coordinated strategy to mitigate copper toxicity, including the upregulation of exopolysaccharide biosynthesis for extracellular protection, enhanced protein quality control mechanisms to manage misfolded proteins, and increased ribonucleoprotein and protein biosynthesis. This occurred alongside the repression of iron uptake genes to reduce oxidative stress caused by Fenton reactions (Fig. 11G; Fig. S3D).

The reasons behind EaR2’s higher copper sensitivity could involve differential regulation of key DEGs for copper homeostasis in comparison to Ea273. However, no differences between EaR2 and Ea273 were observed in essential homeostasis genes (e.g., *copA* and *cueO*). The differences observed in other genes such as *spy, hpt, rcsA, EAMY_RS19660, sitC,* and *rprA* (Tables 3 and 4, Table S8) suggest an overcompensation for higher copper sensitivity, leading to more elaborate responses to maintain copper levels below toxic values.

### Conclusions

This study characterized for the first time *E. amylovora* strains with marked sensitivity to copper. We integrated phenotypic and transcriptomic analyses to unravel short-term and long-term copper response mechanisms in *E. amylovora,* revealing important contrasts between copper-hypersensitive strains and strains with regular copper tolerance.

Phenotypic profiling revealed traits common to copper-hypersensitive strains, including slower growth, elevated sensitivity to paraquat and cadmium, and maladaptive copper-induced EPS overproduction. Additionally, under our experimental conditions, overnight copper pre-exposure either showed no protective effect or actually reduced survival of copper-sensitive strains when exposed to copper shock. Prolonged copper exposure did not reduce the capacity *of E. amylovora* to cause fire blight symptoms in detached fruit. These observations might have practical implications for refining copper-based disease management strategies.

RNA-Seq results, which revealed conserved regulation of copper homeostasis genes in both copper-hypersensitive and tolerant strains (e.g., upregulation of *copA/cueO* and *hpt* repression), suggesting that differential copper sensitivity stems from factors beyond core copper homeostasis mechanisms. Additionally, while both strains activated similar immediate responses to copper shock, their adaptation strategies to prolonged copper exposure diverged significantly. The copper-tolerant strain Ea273 maintained cellular homeostasis through targeted activation of copper resistance genes and downregulation of nonessential pathways and pathways potentially improving oxidative stress resistance, such as histidine catabolism genes. In contrast, EaR2 developed a broader, more resource-intensive response, characterized by upregulation of EPS biosynthesis genes, enhanced protein quality control systems, and extensive stress response activation. EaR2 also exhibited distinct metabolic reprogramming, with an important downregulation of iron uptake-related genes and shifting resources from growth and motility toward copper defense mechanisms.

The divergent transcriptomic profiles between strains suggest two non-exclusive possibilities: i) hypersensitive strains like EaR2 deploy energy-intensive but ineffective detoxification responses, leading to resource exhaustion and observed phenotypic vulnerabilities; or ii) inherent cellular defects such as altered membrane composition (86) lead to extended damage by copper and trigger exaggerated but insufficient stress responses, explaining enhanced copper, cadmium, and paraquat susceptibilities. Whether these extensive adaptations are the cause or consequence of copper hypersensitivity remains to be determined. Our comprehensive analysis of copper responses in *E. amylovora* provides a strong foundation for future mechanistic studies and may guide the development of more effective fire blight control strategies.

## MATERIALS AND METHODS

### Bacterial strains and growth conditions

The copper-hypersensitive strains EaR2 (Ea HCr1) and Ea17 (Ea CB15) were isolated from symptomatic apple rootstocks in orchards in New York State, U.S.A. (87). Ea19 (Ea RedCA13), with intermediate copper tolerance, was isolated from red crabapple in a nursery in Tennessee, U.S.A. (88). The American strain Ea273, with standard copper tolerance, was used as a control. For simplicity, throughout the manuscript, we will refer to Ea273 as a “copper-tolerant strain,” though this designation reflects standard copper tolerance in *E. amylovora,* rather than high/increased copper resistance.

LB medium (89) was used for inoculum preparation and plate counts. *E. amylovora* was grown at 28°C, and liquid cultures were incubated with shaking at 180 rpm.

### Identification of copper-sensitive strains

The unusually high copper susceptibility of EaR2 was detected during simultaneous *E. amylovora* isolation on King’s B agar (KB) (90), RESC agar (28), and CCT agar (91) from a symptomatic apple rootstock. Numerous *E. amylovora*-like colonies grew on KB and CCT media, but no characteristic pale-yellow colonies were isolated on RESC medium containing 1.5 mM copper (II) sulfate (CuSO_4_•5H_2_O).

Strains Ea17 and Ea19 were identified within a collection of *E. amylovora* strains. Ea17 failed to form colonies after stab-inoculation on RESC medium, while Ea19 showed intermediate phenotypes between EaR2 and Ea273 in disk assays on MBMANic with 15 mM CuSO_4_, and spot dilution assays on KB with 0–2.5 mM CuSO_4_ performed as in previous works with other stressors (5, 92).

### Relative quantification of amylovoran and levan

Relative amylovoran production in minimal basal medium A (30, 31) containing 1% sorbitol and 0.05% nicotinic acid (MBMANic) was measured at A_600_ nm in overnight culture supernatants mixed with 50 mg/mL cetylpyridinium chloride (CPC) (30). Sorbitol in this medium enhances amylovoran production in *E. amylovora.* Levan production in nutrient broth (Difco Laboratories Inc., Detroit, MI) was measured at A_580_ nm after mixing 24 h culture supernatants with levansucrase (Lsc) buffer containing 50 mM PBS, 2 M sucrose, and 0.05% sodium azide. Levan measurements are typically tested in rich media without sucrose, like NB or LB, favoring the release of levansucrase to the medium. EPS measurements were expressed relative to the A_600_ nm of the cell cultures (30). Separate uninoculated control samples for each copper concentration were used as blanks.

To compare the effects of the same copper concentrations on amylovoran and levan production simultaneously, we also tested exopolysaccharide (EPS) production in LB and LB with 0–2.5 mM CuSO_4_. We chose this medium because the amylovoran-inducing minimal medium does not promote levan production, but *E. amylovora* produces both EPS in LB. Briefly, 24 h culture supernatants were used to measure both EPSs with CPC and Lsc buffer separately (5). Appropriate sample dilutions before absorbance measurements were performed when required.

These assays were performed in triplicate, in two independent repeats.

### Virulence assays

Virulence was assessed using immature fruitlets from hosts with varying fire blight susceptibility, apple cv. Red Delicious and pear cvs. Bosc and Bartlett. Fruitlets were thoroughly washed with soap and water, rinsed, surface-sterilized in 70% ethanol with 1,000 ppm food-grade natamycin for 60 s, and dried in a laminar flow cabinet. Seven to eight fruits per strain were wound-inoculated using sterile pipette tips and 3 × 10^3^ CFU/wound. Fruits were incubated at 28°C and disease severity monitored over time (92).

### Growth-inhibition disk assays to test hydrogen peroxide (H_2_O_2_), paraquat, and cadmium sensitivity

*E. amylovora* cultures (0.1 mL) adjusted to 3 × 10^8^ CFU/mL in PBS were spread-plated on MBMANic with 0.2% glycerol as the carbon source. Sterile filter paper disks were placed on the agar surface, loaded with 5 µL of 1 M H_2_O_2_, 50 mM paraquat, or 10 mM CdSO_4_ and then dried under the hood. Reagent concentrations were optimized in preliminary assays. After 48 h incubation at 28°C, growth inhibition halo sizes around the paper discs were measured.

### Survival in tobacco leaf apoplast

*E. amylovora* survival in tobacco leaves (*Nicotiana tabacum* cv. Burley was assessed according to a previous protocol (30). Leaves were infiltrated with six 25-µL spots per strain (3 × 10^8^ CFU/mL in PBS). Hypersensitive response and bacterial survival in inoculated sections were evaluated at 0 and 13 days post-infiltration (dpi). *E. amylovora* concentrations were expressed as CFU/cm^2^ infiltrated area, measured by image analysis with ImageJ version 1.53 a (https://imagej.net/ij/). Each strain was inoculated on separate leaves of three plants.

### Monitoring growth in the presence of copper

Growth curves for Ea273, Ea19, EaR2, and Ea17 were performed in LB amended with 0, 0.1, 0.5, 1, and 3 mM CuSO_4_, respectively. The medium was inoculated at a starting A_600_ nm of 0.05, incubated at 28°C (180 rpm), and bacterial growth was monitored spectrophotometrically every 2 h for 24 h in two independent experiments assessed in triplicate. The effects of copper on *E.* amylovora’s growth were determined as differences in AUCs. The time to reach the mid-log phase under each condition was assessed by non-linear regression using GraphPad Prism (version 10.4.1). Doubling time during exponential growth was calculated from optical density measurements (93).

### Copper minimum inhibitory concentration (MIC)

The copper MIC was evaluated in mannitol-glutamate-yeast extract (MGY) broth, a standard medium with low copper-binding capacity used for copper resistance measurements in *Pseudomonas* studies (94). The experiment was repeated twice, in triplicate, and the MIC was calculated by nonlinear regression as the copper concentration reducing *E. amylovora* populations 95% with respect to the control without copper (IC_95_).

### Effects of copper pre-exposure on *E. amylovora* copper-shock survival and virulence

Overnight LB cultures rinsed twice with PBS were used to inoculate fresh LB with 0, 0.1, 0.5, and 1 mM CuSO_4_. After 18 h growth at 28°C (180 rpm), copper in the extracellular medium was removed by rinsing cells twice with PBS. Cells were then resuspended at 10^6^ CFU/mL in 0.9% NaCl. Copper-shock survival was assessed by comparing cell concentrations before and after 5 min exposure to 4 mM CuSO_4_.

To assess potential effects of copper pre-exposure on virulence, 18 h *E. amylovora* cultures in LB and LB with 0.5 mM CuSO_4_, rinsed twice with PBS and wound-inoculated (10^3^ CFU/wound) into immature “Bartlett” fruitlets. After 4 days, virulence was quantified by measuring necrotic areas using image analysis (Image J, version 1.53a).

### RNA-Seq analysis of *E. amylovora* copper shock and copper adaptation responses

To differentiate immediate copper detoxification responses from adaptive strategies during prolonged copper exposure, we compared transcriptomic profiles of copper-tolerant (Ea273) and copper-hypersensitive (EaR2) strains under both copper shock and extended copper exposure conditions. Overnight cultures in LB rinsed with PBS were inoculated at A_600_ nm of 0.05 into fresh medium and grown at 28°C (180 rpm). Copper-shock response was assessed by exposing mid-log cultures in LB to 1 mM CuSO_4_ for 5 min. Copper adaptation responses were studied in cells grown in LB with 1 mM CuSO_4_ (sampled at the mid-exponential phase and after 20 h growth, in the stationary phase) and with 3 mM CuSO4 (sampled after 20 h, in the stationary phase). Control samples without copper were collected for each condition. All treatments were performed in three independent assays. A scheme of the experimental design is provided in Fig. S4.

To address copper’s effects on RNA yield and quality during RNA extractions, cells were pelleted (10,000 *× g*, 2.5 min), rinsed with PBS, and cell pellets treated with RNAprotect Bacteria Reagent (Qiagen, Valencia, CA) diluted in PBS according to the manufacturer’s instructions. Treated cell pellets were stored at −80°C until use. RNA was extracted according to Rivas et al. (95) and cleaned and concentrated according to Kharadi et al. (96). RNA yield and quality were measured via spectrophotometry and agarose-gel electrophoresis.

RNA libraries were prepared at Cornell University’s TREx facility, using NEBNext rRNA Depletion Kit (Bacteria) (New England Biolabs, U.S.A.) and NEBNext Ultra II Directional Library Prep Kit (New England Biolabs, U.S.A.) for Illumina (1,000 ng total RNA) and sequenced on Illumina NovaSeq Sequencer, producing 2 × 150 nucleotide paired-end reads.

RNA-Seq data were analyzed as described in reference 97. Fastp (98) was used for adapter/quality trimming. FastQC was used for quality control. HISAT2 (99) was used to map the clean reads to *E. amylovora* CFBP 1430 genome (assembly GCF_000091565.1). StringTie (100) was used for transcript assembly. Individual transcript assemblies from multiple samples were merged into a unified set of transcripts with StringTie merge, and the merged file was used as reference in a new run of StringTie, providing the gene count files for differential expression analysis, functional annotation, and data visualization. For differential expression analysis, voom-transformed data were used for linear modeling in limma (101, 102), with significance at |log_2_FC| ≥ 1.5 and false discovery rate (FDR) ≤ 0.05.

Heatmaps and principal component analysis were performed with iDEP 2.01 (http://ge-lab.org/idep/). Gene function annotation and gene ontology (GO) enrichment were analyzed with DAVID (https://david.ncifcrf.gov) (103) and STRING (https://string-db.org) (104) platforms. Expression data were validated through correlation with RT-PCR data.

## Data Availability

The data discussed in this publication have been deposited in NCBI's Gene Expression Omnibus (105) and are accessible through GEO Series accession number GSE288253.

## SUPPLEMENTAL MATERIAL

Supplemental material aem.00334-25-s0001.docx Tables S1 to S8; Fig. S1 to S4.

ASM does not own the copyrights to Supplemental Material that may be linked to, or accessed through, an article. The authors have granted ASM a non-exclusive, world-wide license to publish the Supplemental Material files. Please contact the corresponding author directly for reuse.
