# Supplementary material for: Copper-induced stress mechanisms in Erwinia amylovora: a comparative phenotypic and transcriptomic study using copper-sensitive and -tolerant strains
Source: Appl Environ Microbiol. 2025 Sep 18;91(10):e00334-25. doi: 10.1128/aem.00334-25 (PMC12542790; doi:10.1128/aem.00334-25)
Supplement: Supplemental material — Tables S1 to S8; Fig. S1 to S4. [file aem.00334-25-s0001.docx]

**Supplementary information**

**Table S1.** DEGs in EaR2 log-phase cells compared to Ea273^a^

| **Gene ID** | **Symbol** | **Function** | **Log2FC** | **FDR** |
| --- | --- | --- | --- | --- |
| 8912198 | *EAMY_RS33740* | SMP-30/gluconolactonase/LRE family protein | 2.85 | 1.57E-17 |
| 8911765 | *EAMY_RS27355* | glycosyltransferase family 8 protein | 2.86 | 4.92E-17 |
| 8912922 | *aspA* | aspartate ammonia-lyase | 2.66 | 5.67E-17 |
| 8911454 | *EAMY_RS22430* | OsmC family protein | 2.69 | 9.67E-17 |
| 8911260 | *EAMY_RS19365* | porin | -4.05 | 2.08E-16 |
| 8913554 | *EAMY_RS28725* | aldo/keto reductase | -2.50 | 2.08E-16 |
| 8911427 | *lysM* | peptidoglycan-binding protein LysM | 3.19 | 2.85E-16 |
| 8912326 | *EAMY_RS28070* | DUF3320 domain-containing protein | 2.96 | 2.85E-16 |
| 8912211 | *EAMY_RS33860* | AsmA family protein | 2.40 | 2.85E-16 |
| 8912384 | *EAMY_RS23965* | NAD-dependent succinate-semialdehyde dehydrogenase | 4.14 | 5.95E-16 |
| 8913422 | *hchA* | glyoxalase III HchA | 2.21 | 1.81E-15 |
| 8911621 | *EAMY_RS25615* | catalase | 2.74 | 1.84E-15 |
| 8911411 | *EAMY_RS21710* | biofilm development regulator YmgB/AriR family protein | 2.20 | 3.61E-15 |
| 8912570 | *EAMY_RS19625* | SDR family oxidoreductase | 5.22 | 4.50E-15 |
| 8914548 | *arnA* | bifunctional UDP-4-amino-4-deoxy-L-arabinose formyltransferase/UDP-glucuronic acid oxidase ArnA | 2.49 | 4.50E-15 |
| 8912325 | *EAMY_RS27460* | Lrp/AsnC family transcriptional regulator | 2.99 | 6.16E-15 |
| 8911461 | *arnB* | UDP-4-amino-4-deoxy-L-arabinose aminotransferase | 2.83 | 6.16E-15 |
| 8913400 | *EAMY_RS23235* | glycosyltransferase | 2.53 | 6.16E-15 |
| 8911485 | *EAMY_RS23200* | Yip1 family protein | 3.79 | 7.74E-15 |
| 8912070 | *EAMY_RS32050* | ATP-grasp domain-containing protein | 3.41 | 7.74E-15 |
| 8911428 | *EAMY_RS22125* | YbaY family lipoprotein | 1.96 | 8.89E-15 |
| 8912962 | *EAMY_RS23395* | DksA/TraR family C4-type zinc finger protein | 2.27 | 9.20E-15 |
| 23673343 | *arnC* | undecaprenyl-phosphate 4-deoxy-4-formamido-L-arabinose transferase | 2.78 | 1.24E-14 |
| 8913310 | *EAMY_RS24375* | hypothetical protein | 2.04 | 2.38E-14 |
| 8911711 | *EAMY_RS26750* | hypothetical protein | 2.94 | 2.61E-14 |
| 8913162 | *EAMY_RS33810* | serralysin family metalloprotease | 2.46 | 2.61E-14 |
| 8914226 | *EAMY_RS29215* | M4 family metallopeptidase | 2.69 | 3.24E-14 |
| 8913107 | *hrpG* | type III secretion system chaperone HrpG | -2.68 | 3.24E-14 |
| 8913614 | *EAMY_RS23600* | ATP-dependent endonuclease | 2.29 | 3.24E-14 |
| 8913594 | *EAMY_RS31375* | nucleoside hydrolase | -1.93 | 3.70E-14 |
| 8914738 | *EAMY_RS33880* | dicarboxylate/amino acid:cation symporter | -1.88 | 4.37E-14 |
| 8914381 | *grxB* | glutaredoxin 2 | 1.91 | 4.72E-14 |
| 8913507 | *EAMY_RS22435* | RcnB family protein | 3.14 | 5.19E-14 |
| 8914710 | *EAMY_RS33050* | hypothetical protein | 2.38 | 5.19E-14 |
| 8912464 | *EAMY_RS19750* | cytosine permease | 1.91 | 5.54E-14 |
| 8912569 | *EAMY_RS28195* | endonuclease/exonuclease/phosphatase family protein | -1.78 | 5.54E-14 |
| 8913261 | *EAMY_RS34315* | phage holin, lambda family | 2.48 | 5.97E-14 |
| 8914341 | *ybiO* | mechanosensitive channel protein | 2.02 | 5.97E-14 |
| 8914744 | *EAMY_RS28675* | SMP-30/gluconolactonase/LRE family protein | 2.29 | 5.98E-14 |
| 8914331 | *EAMY_RS23085* | NADP-dependent oxidoreductase | 2.88 | 8.38E-14 |
| 41697646 | *glgX* | glycogen debranching protein GlgX | 2.40 | 1.04E-13 |
| 69102811 | *EAMY_RS36740* | pseudo | 1.63 | 1.19E-13 |
| 8913206 | *kdpA* | potassium-transporting ATPase subunit KdpA | 2.06 | 1.23E-13 |
| 8912959 | *dapD* | 2,3,4,5-tetrahydropyridine-2,6-dicarboxylate N-succinyltransferase | -2.76 | 1.26E-13 |
| 8913266 | *EAMY_RS32730* | Na/Pi cotransporter family protein | 2.21 | 1.38E-13 |
| 8912994 | *EAMY_RS33745* | hypothetical protein | 2.09 | 1.44E-13 |
| 8911735 | *flhC* | flagellar transcriptional regulator FlhC | 2.33 | 1.87E-13 |
| 41697605 | *EAMY_RS35880* | pseudo | 2.79 | 1.99E-13 |
| 8911532 | *EAMY_RS24050* | MarR family transcriptional regulator | 2.09 | 2.25E-13 |
| 8911975 | *EAMY_RS31040* | collagen-like triple helix repeat-containing protein | 2.69 | 3.28E-13 |
| 8913685 | *EAMY_RS34310* | glycoside hydrolase family 68 protein | 2.48 | 3.28E-13 |
| 8911850 | *EAMY_RS28690* | hypothetical protein | 2.09 | 3.28E-13 |
| 8912110 | *EAMY_RS32270* | glucose 1-dehydrogenase | 2.47 | 3.44E-13 |
| 8913785 | *acnA* | aconitate hydratase AcnA | 2.19 | 3.82E-13 |
| 8913273 | *malP* | maltodextrin phosphorylase | 2.20 | 3.88E-13 |
| 8914328 | *EAMY_RS23000* | protein YbgS | 2.38 | 5.33E-13 |
| 8914013 | *EAMY_RS25670* | FAD-binding protein | 1.91 | 5.80E-13 |
| 8914043 | *dkgA* | 2,5-didehydrogluconate reductase DkgA | 5.60 | 5.98E-13 |
| 8914604 | *mprA* | transcriptional repressor MprA | 2.81 | 6.88E-13 |
| 8913112 | *EAMY_RS20005* | type III secretion system chaperone | -1.56 | 7.20E-13 |
| 8912475 | *astD* | succinylglutamate-semialdehyde dehydrogenase | 2.49 | 7.43E-13 |
| 8911915 | *EAMY_RS29850* | DUF2002 family protein | 2.86 | 8.23E-13 |
| 8914864 | *EAMY_RS22485* | glucose/quinate/shikimate family membrane-bound PQQ-dependent dehydrogenase | -1.82 | 8.23E-13 |
| 8912608 | *EAMY_RS21815* | DUF4810 domain-containing protein | -2.08 | 9.75E-13 |
| 8912108 | *EAMY_RS32265* | DUF4354 family protein | 1.75 | 1.01E-12 |
| 8912454 | *EAMY_RS30480* | MFS transporter | 1.96 | 1.10E-12 |
| 8914405 | *wbaP* | undecaprenyl-phosphate galactose phosphotransferase WbaP | 2.18 | 1.17E-12 |
| 8913719 | *EAMY_RS26820* | MFS transporter | -2.34 | 1.18E-12 |
| 8911980 | *osmY* | molecular chaperone OsmY | 4.70 | 1.27E-12 |
| 8913649 | *EAMY_RS26530* | YeaH/YhbH family protein | 1.90 | 1.42E-12 |
| 8911654 | *EAMY_RS25865* | hypothetical protein | 6.58 | 1.78E-12 |
| 8914819 | *EAMY_RS31925* | lipocalin family protein | 2.31 | 1.90E-12 |
| 8914874 | *EAMY_RS25055* | fructosamine kinase family protein | 2.39 | 1.91E-12 |
| 8911694 | *yeaG* | protein kinase YeaG | 2.17 | 2.00E-12 |
| 8911612 | *EAMY_RS25565* | hypothetical protein | 2.92 | 2.18E-12 |
| 8912523 | *proY* | proline-specific permease ProY | -1.64 | 2.32E-12 |
| 8911487 | *dld* | D-lactate dehydrogenase | 1.59 | 2.58E-12 |
| 8913929 | *EAMY_RS21915* | DUF3251 domain-containing protein | 2.57 | 2.63E-12 |
| 23673361 | *EAMY_RS25875* | DapH/DapD/GlmU-related protein | 6.24 | 2.73E-12 |
| 8913497 | *EAMY_RS20015* | HrpW-specific chaperone | -1.87 | 3.69E-12 |
| 8912822 | *EAMY_RS24220* | flagellar basal body P-ring protein FlgI | -1.88 | 3.81E-12 |
| 8912477 | *astA* | arginine N-succinyltransferase | 2.39 | 3.83E-12 |
| 41697645 | *glgC* | glucose-1-phosphate adenylyltransferase | 1.91 | 4.20E-12 |
| 8912190 | *EAMY_RS33655* | alpha/beta hydrolase | 2.07 | 4.34E-12 |
| 8911699 | *EAMY_RS26565* | SpoVR family protein | 1.84 | 4.56E-12 |
| 8911810 | *EAMY_RS28200* | trehalose-6-phosphate synthase | -1.89 | 4.56E-12 |
| 8911684 | *EAMY_RS26265* | BON domain-containing protein | 2.48 | 4.75E-12 |
| 8914385 | *EAMY_RS24120* | cytochrome b | 2.25 | 5.14E-12 |
| 8911974 | *EAMY_RS31035* | POTRA domain-containing protein | 2.09 | 5.44E-12 |
| 8912414 | *EAMY_RS27680* | phage tailspike protein | 1.51 | 5.65E-12 |
| 8912590 | *EAMY_RS19755* | PAS and helix-turn-helix domain-containing protein | 1.72 | 6.71E-12 |
| 8914077 | *ybbA* | putative ABC transporter ATP-binding protein YbbA | 2.25 | 7.25E-12 |
| 8914718 | *malQ* | 4-alpha-glucanotransferase | 2.34 | 8.24E-12 |
| 8912357 | *EAMY_RS31520* | SulP family inorganic anion transporter | 1.69 | 8.93E-12 |
| 8912498 | *EAMY_RS29055* | sensor domain-containing diguanylate cyclase | 1.69 | 9.32E-12 |
| 8914442 | *EAMY_RS19660* | YlaC family protein | -1.99 | 1.01E-11 |
|  | *MSTRG.824.1* | Unannotated transcript | 3.27 | 1.14E-11 |
| 23673396 | *fhuA* | pseudo | -2.46 | 1.18E-11 |
| 8913525 | *gspS* | type II secretion system pilot lipoprotein GspS | 1.83 | 1.21E-11 |
| 8914836 | *EAMY_RS19210* | helicase HerA-like C-terminal domain-containing protein | 1.65 | 1.21E-11 |
| 8912402 | *EAMY_RS19205* | DUF445 family protein | 1.69 | 1.22E-11 |
| 8914914 | *EAMY_RS31590* | DUF1090 domain-containing protein | 1.63 | 1.45E-11 |
| 8914223 | *EAMY_RS27515* | EmmdR/YeeO family multidrug/toxin efflux MATE transporter | 2.11 | 1.67E-11 |
| 8912892 | *panB* | 3-methyl-2-oxobutanoate hydroxymethyltransferase | -2.44 | 1.74E-11 |
| 23673438 | *EAMY_RS33805* | protease inhibitor Inh/omp19 family protein | 1.87 | 1.77E-11 |
| 8912658 | *EAMY_RS23490* | HAAAP family serine/threonine permease | -2.06 | 1.80E-11 |
| 8912952 | *glgA* | glycogen synthase GlgA | 1.84 | 1.80E-11 |
| 8912472 | *ompC* | porin OmpC | -1.80 | 1.84E-11 |
| 8914873 | *ghoS* | type V toxin-antitoxin system endoribonuclease antitoxin GhoS | 3.14 | 1.91E-11 |
| 8912857 | *fliG* | flagellar motor switch protein FliG | -2.67 | 2.30E-11 |
| 8913691 | *ptsI* | phosphoenolpyruvate-protein phosphotransferase PtsI | -1.98 | 2.32E-11 |
| 8914339 | *dps* | DNA starvation/stationary phase protection protein Dps | 1.62 | 3.27E-11 |
| 8913100 | *sctW* | type III secretion system gatekeeper subunit SctW | -2.50 | 3.90E-11 |
| 8913125 | *EAMY_RS19845* | ATP-grasp domain-containing protein | -1.73 | 5.87E-11 |
| 8914957 | *brnQ* | branched-chain amino acid transporter carrier protein BrnQ | -1.63 | 6.11E-11 |
| 8914779 | *pldA* | phospholipase A | -1.56 | 7.20E-11 |
| 8913101 | *EAMY_RS19955* | EscI/YscI/HrpB family type III secretion system inner rod protein | -1.97 | 7.65E-11 |
| 8911264 | *EAMY_RS19500* | creatininase family protein | 1.67 | 8.05E-11 |
| 8914073 | *osmE* | osmotically-inducible lipoprotein OsmE | 1.98 | 8.43E-11 |
| 8911753 | *amyA* | alpha-amylase | 2.20 | 8.45E-11 |
| 8914290 | *EAMY_RS22080* | thioesterase family protein | 1.61 | 8.45E-11 |
| 8912476 | *astE* | succinylglutamate desuccinylase | 2.06 | 8.58E-11 |
| 8912812 | *flgD* | flagellar hook assembly protein FlgD | -2.15 | 8.61E-11 |
| 8914248 | *proS* | proline--tRNA ligase | -1.72 | 8.61E-11 |
| 8912808 | *flgB* | flagellar basal body rod protein FlgB | -2.77 | 1.16E-10 |
| 8913903 | *mglC* | galactose/methyl galactoside ABC transporter permease MglC | -1.86 | 1.16E-10 |
| 8911266 | *EAMY_RS19510* | nucleoside 2-deoxyribosyltransferase | 1.54 | 1.16E-10 |
| 8911322 | *tkt* | transketolase | -1.96 | 1.42E-10 |
| 8914411 | *prs* | ribose-phosphate diphosphokinase | -1.51 | 1.49E-10 |
| 8914506 | *fliZ* | flagella biosynthesis regulatory protein FliZ | -1.86 | 1.59E-10 |
| 8911816 | *EAMY_RS28335* | ADP-ribosyltransferase | 3.95 | 1.74E-10 |
| 8914364 | *EAMY_RS23780* | amino acid aminotransferase | -2.27 | 1.97E-10 |
| 8911281 | *EAMY_RS19685* | ABC transporter substrate-binding protein | -1.74 | 2.00E-10 |
| 8912630 | *EAMY_RS25640* | DUF4385 domain-containing protein | 2.16 | 2.35E-10 |
| 8911776 | *EAMY_RS27465* | Dabb family protein | 2.71 | 2.53E-10 |
| 8912816 | *EAMY_RS24205* | flagellar basal body rod protein FlgF | -2.12 | 2.66E-10 |
| 8913683 | *pstS* | phosphate ABC transporter substrate-binding protein PstS | 2.09 | 2.74E-10 |
| 8911628 | *EAMY_RS25655* | SrfA family protein | -2.24 | 3.30E-10 |
| 8912381 | *gltP* | glutamate/aspartate:proton symporter GltP | -1.96 | 3.30E-10 |
| 8911973 | *EAMY_RS31030* | zinc-binding alcohol dehydrogenase family protein | 1.54 | 3.52E-10 |
| 8911609 | *EAMY_RS25555* | Glu/Leu/Phe/Val dehydrogenase | -1.81 | 3.73E-10 |
| 8912365 | *fabF* | beta-ketoacyl-ACP synthase II | -1.77 | 3.74E-10 |
| 8912814 | *flgE* | flagellar hook protein FlgE | -2.17 | 4.44E-10 |
|  | *MSTRG.1243.1* | Unannotated transcript | 2.41 | 5.08E-10 |
| 8913139 | *hutH* | histidine ammonia-lyase | -3.05 | 5.30E-10 |
| 8912462 | *EAMY_RS23575* | lipoprotein | 1.68 | 5.77E-10 |
| 8911851 | *EAMY_RS28695* | universal stress protein | 2.15 | 7.10E-10 |
| 8912871 | *fliN* | flagellar motor switch protein FliN | -2.29 | 8.32E-10 |
| 8913093 | *hrpT* | HrpT family type III secretion system protein | -2.59 | 8.65E-10 |
| 8911484 | *EAMY_RS23190* | DedA family protein | 1.72 | 1.03E-09 |
| 8913110 | *EAMY_RS19840* | hypothetical protein | -2.36 | 1.13E-09 |
| 8914507 | *EAMY_RS24385* | M15 family metallopeptidase | 1.87 | 1.16E-09 |
| 8913013 | *rimJ* | ribosomal protein S5-alanine N-acetyltransferase | 1.52 | 1.27E-09 |
| 8912818 | *flgG* | flagellar basal-body rod protein FlgG | -1.82 | 1.30E-09 |
| 23673369 | *EAMY_RS27215* | DUF2158 domain-containing protein | 3.04 | 1.33E-09 |
| 8913113 | *sctN* | type III secretion system ATPase SctN | -2.10 | 1.39E-09 |
| 8912287 | *EAMY_RS19225* | integrase arm-type DNA-binding domain-containing protein | 1.66 | 1.39E-09 |
| 8913029 | *EAMY_RS19885* | FliM/FliN family flagellar motor switch protein | -1.66 | 1.43E-09 |
| 43500273 | *EAMY_RS36170* | hypothetical protein | 4.72 | 2.02E-09 |
| 8914387 | *EAMY_RS24100* | MysB family protein | 1.84 | 2.06E-09 |
| 8914571 | *dapE* | succinyl-diaminopimelate desuccinylase | -1.63 | 2.08E-09 |
| 8913535 | *panC* | pantoate--beta-alanine ligase | -2.78 | 2.09E-09 |
| 8913134 | *EAMY_RS23320* | HutD family protein | -2.94 | 2.10E-09 |
| 8913095 | *sctD* | type III secretion system inner membrane ring subunit SctD | -2.28 | 2.15E-09 |
| 8913914 | *EAMY_RS24930* | carbohydrate porin | -1.98 | 2.15E-09 |
| 8913882 | *rimM* | ribosome maturation factor RimM | -1.83 | 2.50E-09 |
| 8911655 | *EAMY_RS25880* | hypothetical protein | 5.43 | 2.52E-09 |
| 8913825 | *rpsJ* | 30S ribosomal protein S10 | -2.65 | 2.60E-09 |
| 8913311 | *mglB* | galactose/glucose ABC transporter substrate-binding protein MglB | -1.59 | 3.19E-09 |
| 8912832 | *flgA* | flagellar basal body P-ring formation chaperone FlgA | -1.69 | 3.27E-09 |
| 8911584 | *EAMY_RS24980* | aminotransferase class III-fold pyridoxal phosphate-dependent enzyme | 1.77 | 3.55E-09 |
| 77383446 | *EAMY_RS36780* | hypothetical protein | 2.91 | 3.70E-09 |
| 8914380 | *EAMY_RS24115* | YceI family protein | 1.76 | 4.15E-09 |
| 8913329 | *pncC* | nicotinamide-nucleotide amidase | 1.68 | 4.28E-09 |
| 8912444 | *EAMY_RS26925* | flagellar protein FlhE | -2.03 | 4.29E-09 |
| 8914318 | *EAMY_RS22835* | 2-thiouracil desulfurase family protein | 1.92 | 4.32E-09 |
| 8911801 | *EAMY_RS28075* | restriction endonuclease | 3.71 | 4.33E-09 |
| 8912248 | *mobA* | molybdenum cofactor guanylyltransferase MobA | 2.05 | 4.44E-09 |
| 8914591 | *trxC* | thioredoxin TrxC | 2.02 | 4.45E-09 |
| 8914629 | *EAMY_RS24695* | YgdI/YgdR family lipoprotein | 2.43 | 4.45E-09 |
| 8914764 | *yidC* | membrane protein insertase YidC | -2.55 | 4.56E-09 |
| 8914619 | *cas7e* | type I-E CRISPR-associated protein Cas7/Cse4/CasC | 1.60 | 4.91E-09 |
| 8912749 | *emrB* | multidrug efflux MFS transporter permease subunit EmrB | -1.57 | 5.34E-09 |
| 8913432 | *rpoS* | RNA polymerase sigma factor RpoS | 1.68 | 5.45E-09 |
| 23673348 | *yccX* | acylphosphatase | 2.01 | 5.59E-09 |
| 8913103 | *sctJ* | type III secretion inner membrane ring lipoprotein SctJ | -1.80 | 5.74E-09 |
| 8911874 | *EAMY_RS29060* | M48 family metallopeptidase | -1.73 | 6.15E-09 |
| 8914808 | *EAMY_RS32655* | CsbD family protein | 3.34 | 6.31E-09 |
| 8912210 | *EAMY_RS33855* | MFS transporter | -1.63 | 6.31E-09 |
| 8913671 | *lpxO* | lipid A hydroxylase LpxO | 2.10 | 6.35E-09 |
| 8914698 | *rplQ* | 50S ribosomal protein L17 | -2.10 | 6.87E-09 |
| 8912129 | *EAMY_RS32450* | glutathione S-transferase | 1.85 | 7.22E-09 |
| 8913135 | *hutI* | imidazolonepropionase | -1.82 | 8.66E-09 |
| 8911420 | *EAMY_RS21810* | CsgG/HfaB family protein | -1.53 | 8.83E-09 |
| 8913053 | *hutU* | urocanate hydratase | -2.28 | 9.04E-09 |
| 8912845 | *EAMY_RS27175* | HNH endonuclease signature motif containing protein | -1.57 | 9.61E-09 |
| 8913593 | *uspB* | universal stress protein UspB | 2.06 | 1.01E-08 |
| 8912764 | *EAMY_RS19620* | MFS transporter | -2.09 | 1.12E-08 |
| 8913634 | *pqqE* | pyrroloquinoline quinone biosynthesis protein PqqE | 2.06 | 1.46E-08 |
| 8911600 | *araA* | L-arabinose isomerase | 4.92 | 1.50E-08 |
| 8911625 | *EAMY_RS25630* | methyl-accepting chemotaxis protein | 2.00 | 1.50E-08 |
| 8913059 | *hisD* | histidinol dehydrogenase | -2.05 | 1.57E-08 |
| 8913050 | *tomB* | Hha toxicity modulator TomB | 1.62 | 1.65E-08 |
| 8914241 | *pcnB* | polynucleotide adenylyltransferase PcnB | -2.74 | 1.82E-08 |
| 8914006 | *ansA* | asparaginase | -1.98 | 1.85E-08 |
| 8913823 | *rplJ* | 50S ribosomal protein L10 | -2.19 | 1.85E-08 |
| 8912861 | *fliI* | flagellar protein export ATPase FliI | -1.83 | 1.99E-08 |
| 8911406 | *EAMY_RS21655* | hypothetical protein | 2.03 | 2.08E-08 |
| 8912810 | *flgC* | flagellar basal body rod protein FlgC | -2.00 | 2.24E-08 |
| 8914550 | *arnT* | lipid IV(A) 4-amino-4-deoxy-L-arabinosyltransferase | 1.51 | 2.51E-08 |
| 8911897 | *csiE* | stationary phase inducible protein CsiE | 1.65 | 2.66E-08 |
| 8912601 | *EAMY_RS21630* | hypothetical protein | 2.17 | 3.07E-08 |
| 8913824 | *rplW* | 50S ribosomal protein L23 | -1.78 | 3.07E-08 |
| 8914121 | *rplS* | 50S ribosomal protein L19 | -1.91 | 3.11E-08 |
| 8912516 | *gntX* | DNA utilization protein GntX | 1.65 | 3.45E-08 |
| 8912004 | *tssB* | type VI secretion system contractile sheath small subunit | -2.59 | 3.53E-08 |
| 8912010 | *tssJ* | type VI secretion system lipoprotein TssJ | -4.01 | 3.62E-08 |
| 8912790 | *rpsP* | 30S ribosomal protein S16 | -2.47 | 3.72E-08 |
| 8912927 | *galT* | galactose-1-phosphate uridylyltransferase | -3.62 | 3.72E-08 |
| 8914519 | *hisG* | ATP phosphoribosyltransferase | -1.81 | 4.20E-08 |
| 8914242 | *hpt* | hypoxanthine phosphoribosyltransferase | 1.63 | 4.46E-08 |
| 8911939 | *EAMY_RS30450* | SDR family oxidoreductase | -1.62 | 4.50E-08 |
| 77383444 | *EAMY_RS36770* | hypothetical protein | 2.63 | 4.52E-08 |
| 8912009 | *tssK* | type VI secretion system baseplate subunit TssK | -3.33 | 4.56E-08 |
| 23673412 | *EAMY_RS31935* | papain-like cysteine protease family protein | -2.13 | 4.87E-08 |
| 55585579 | *EAMY_RS36455* | transcriptional regulator | 2.59 | 4.92E-08 |
| 8913463 | *rplK* | 50S ribosomal protein L11 | -2.83 | 5.59E-08 |
| 8914153 | *fadA* | acetyl-CoA C-acyltransferase FadA | 1.95 | 7.73E-08 |
| 8911914 | *EAMY_RS29835* | nucleoside-specific channel-forming protein Tsx | -1.66 | 7.73E-08 |
| 8913805 | *lepB* | signal peptidase I | -2.20 | 8.52E-08 |
| 8914211 | *EAMY_RS24380* | mannosyl-3-phosphoglycerate phosphatase-related protein | 2.04 | 9.09E-08 |
| 8912865 | *EAMY_RS24435* | flagellar hook-length control protein FliK | -1.51 | 1.11E-07 |
| 8912521 | *EAMY_RS31930* | entericidin A/B family lipoprotein | 1.54 | 1.14E-07 |
| 8911466 | *EAMY_RS22550* | hypothetical protein | 1.53 | 1.19E-07 |
| 8912329 | *EAMY_RS28755* | formate/nitrite transporter family protein | -2.38 | 1.19E-07 |
| 23673414 | *EAMY_RS32005* | cupin domain-containing protein | 2.15 | 1.22E-07 |
| 8911820 | *ackA* | acetate kinase | -1.55 | 1.37E-07 |
| 8911211 | *quinone* | malate dehydrogenase | -1.80 | 1.41E-07 |
| 8912820 | *EAMY_RS24215* | flagellar basal body L-ring protein FlgH | -1.72 | 1.43E-07 |
| 8913468 | *EAMY_RS33975* | HD domain-containing protein | 1.88 | 1.58E-07 |
| 8914598 | *trmD* | tRNA (guanosine(37)-N1)-methyltransferase TrmD | -1.58 | 1.66E-07 |
| 8912429 | *osmB* | osmotically-inducible lipoprotein OsmB | 3.83 | 1.69E-07 |
| 8911777 | *EAMY_RS27470* | DUF2000 domain-containing protein | 2.24 | 1.81E-07 |
| 8914845 | *EAMY_RS19830* | VirK family protein | 1.55 | 2.08E-07 |
| 8912843 | *flhA* | flagellar biosynthesis protein FlhA | -1.67 | 2.34E-07 |
| 30316911 | *EAMY_RS34980* | hypothetical protein | 2.47 | 2.36E-07 |
| 8914952 | *sseB* | enhanced serine sensitivity protein SseB | -2.04 | 2.36E-07 |
| 8911542 | *murJ* | murein biosynthesis integral membrane protein MurJ | -2.13 | 2.70E-07 |
| 8913192 | *rplU* | 50S ribosomal protein L21 | -1.58 | 2.77E-07 |
| 8912587 | *EAMY_RS24520* | benzoate/H(+) symporter BenE family transporter | -1.58 | 2.81E-07 |
| 8912007 | *tssM* | type VI secretion system membrane subunit TssM | -3.01 | 2.83E-07 |
| 8914700 | *accC* | acetyl-CoA carboxylase biotin carboxylase subunit | -1.77 | 3.22E-07 |
| 8911558 | *EAMY_RS24600* | hypothetical protein | 2.72 | 3.53E-07 |
| 30317028 | *EAMY_RS35565* | ogr/Delta-like zinc finger family protein | 1.86 | 3.56E-07 |
| 8914526 | *EAMY_RS27800* | CesT family type III secretion system chaperone | -3.42 | 3.59E-07 |
| 8913841 | *rpmA* | 50S ribosomal protein L27 | -1.90 | 3.59E-07 |
| 8913826 | *rplC* | 50S ribosomal protein L3 | -1.71 | 4.23E-07 |
| 8914818 | *EAMY_RS24690* | YgdI/YgdR family lipoprotein | 1.91 | 4.57E-07 |
| 8913661 | *proX* | glycine betaine/L-proline ABC transporter substrate-binding protein ProX | -1.79 | 4.65E-07 |
| 8912654 | *cysK* | cysteine synthase A | 2.05 | 4.66E-07 |
| 8911319 | *EAMY_RS20275* | hypothetical protein | 1.62 | 4.70E-07 |
| 8912875 | *fliP* | flagellar type III secretion system pore protein FliP | -1.91 | 4.79E-07 |
| 8913097 | *sctR* | type III secretion system export apparatus subunit SctR | -1.86 | 4.79E-07 |
| 8912016 | *EAMY_RS31370* | PepSY-associated TM helix domain-containing protein | -1.87 | 5.25E-07 |
|  | *MSTRG.1855.1* | Unannotated transcript | 2.94 | 5.44E-07 |
| 8911989 | *tssH* | type VI secretion system ATPase TssH | -2.46 | 5.51E-07 |
| 8914674 | *nusA* | transcription termination factor NusA | -1.59 | 5.55E-07 |
| 8911459 | *EAMY_RS22470* | leucine-rich repeat domain-containing protein | 1.55 | 5.67E-07 |
| 55585553 | *EAMY_RS36325* | hypothetical protein | 2.33 | 5.92E-07 |
| 8913833 | *rpsI* | 30S ribosomal protein S9 | -4.85 | 6.26E-07 |
| 8913428 | *ampH* | D-alanyl-D-alanine-carboxypeptidase/endopeptidase AmpH | -1.57 | 6.35E-07 |
| 8913226 | *lepA* | translation elongation factor 4 | -2.29 | 7.17E-07 |
| 8913504 | *EAMY_RS19950* | Hrp pili protein HrpA | -2.19 | 8.00E-07 |
| 8912932 | *rssB* | two-component system response regulator RssB | 1.53 | 8.45E-07 |
| 8912703 | *gluQRS* | tRNA glutamyl-Q(34) synthetase GluQRS | -2.39 | 8.74E-07 |
| 8913186 | *EAMY_RS23445* | ABC-F family ATPase | -2.34 | 8.96E-07 |
| 8913873 | *rpsL* | 30S ribosomal protein S12 | -1.65 | 9.11E-07 |
| 8914020 | *gutM* | transcriptional regulator GutM | 1.54 | 1.07E-06 |
| 8913106 | *EAMY_RS19975* | HPr kinase | -3.95 | 1.14E-06 |
| 8913830 | *rplL* | 50S ribosomal protein L7/L12 | -2.22 | 1.18E-06 |
| 8913881 | *pnp* | polyribonucleotide nucleotidyltransferase | -1.87 | 1.27E-06 |
| 8914535 | *EAMY_RS27985* | phosphatase PAP2 family protein | -1.80 | 1.27E-06 |
| 23673330 | *EAMY_RS20630* | hypothetical protein | 2.31 | 1.41E-06 |
| 8913109 | *EAMY_RS19920* | sigma-70 family RNA polymerase sigma factor | -1.94 | 1.41E-06 |
| 8914120 | *murI* | glutamate racemase | -2.50 | 1.52E-06 |
| 8913847 | *rpmG* | 50S ribosomal protein L33 | -2.01 | 1.59E-06 |
| 8913014 | *rpmB* | 50S ribosomal protein L28 | -1.93 | 1.72E-06 |
| 8912867 | *fliL* | flagellar basal body-associated protein FliL | -3.01 | 1.94E-06 |
| 8912646 | *ptsH* | phosphocarrier protein Hpr | -1.76 | 2.04E-06 |
| 8914501 | *EAMY_RS26900* | hypothetical protein | -1.52 | 2.40E-06 |
| 8912616 | *EAMY_RS19650* | spore coat protein U domain-containing protein | 3.30 | 2.61E-06 |
| 8914861 | *EAMY_RS25825* | hypothetical protein | 1.60 | 3.08E-06 |
| 8913235 | *EAMY_RS19710* | phytochelatin synthase family protein | -1.56 | 3.57E-06 |
| 8911514 | *ltaE* | low-specificity L-threonine aldolase | 5.64 | 3.58E-06 |
| 23673395 | *EAMY_RS29195* | pseudo | 2.65 | 3.90E-06 |
| 8914132 | *rpsO* | 30S ribosomal protein S15 | -2.33 | 4.38E-06 |
| 30316919 | *EAMY_RS35020* | hypothetical protein | 3.71 | 4.39E-06 |
| 8911748 | *phoH* | phosphate starvation-inducible protein PhoH | 1.56 | 4.59E-06 |
| 8914688 | *rplM* | 50S ribosomal protein L13 | -3.11 | 4.64E-06 |
| 8911991 | *tssE* | type VI secretion system baseplate subunit TssE | -3.27 | 4.65E-06 |
| 8912005 | *tssA* | type VI secretion system protein TssA | -2.23 | 4.80E-06 |
| 8912855 | *fliF* | flagellar basal-body MS-ring/collar protein FliF | -4.05 | 5.06E-06 |
| 8913536 | *panD* | aspartate 1-decarboxylase | -2.78 | 5.30E-06 |
| 8914643 | *EAMY_RS20320* | UDP-3-O-(3-hydroxymyristoyl)glucosamine N-acyltransferase | 1.80 | 5.30E-06 |
| 8913249 | *matP* | macrodomain Ter protein MatP | 1.53 | 5.71E-06 |
| 8913104 | *thpR* | RNA 2',3'-cyclic phosphodiesterase | -2.53 | 5.77E-06 |
| 30316876 | *EAMY_RS34805* | hypothetical protein | 1.56 | 7.10E-06 |
| 8911401 | *EAMY_RS21635* | hypothetical protein | 2.13 | 7.22E-06 |
| 8914426 | *EAMY_RS26105* | LysR family transcriptional regulator | 1.70 | 7.90E-06 |
| 8911802 | *EAMY_RS28085* | acyltransferase | 1.69 | 7.98E-06 |
| 8914284 | *EAMY_RS22265* | MFS transporter | -1.57 | 8.61E-06 |
| 30316843 | *EAMY_RS34640* | hypothetical protein | 2.12 | 9.29E-06 |
| 8912945 | *EAMY_RS33380* | DUF2756 domain-containing protein | 1.52 | 9.75E-06 |
| 8911947 | *EAMY_RS30505* | DUF6543 domain-containing protein | 1.56 | 9.81E-06 |
| 8914795 | *trmA* | tRNA (uridine(54)-C5)-methyltransferase TrmA | -2.88 | 1.03E-05 |
| 8913178 | *sctW* | type III secretion system gatekeeper subunit SctW | 2.02 | 1.13E-05 |
| 8913871 | *rplR* | 50S ribosomal protein L18 | -1.70 | 1.23E-05 |
| 8912859 | *fliH* | flagellar assembly protein FliH | -1.64 | 1.29E-05 |
| 8914466 | *EAMY_RS25135* | heme ABC transporter ATP-binding protein | -1.54 | 1.70E-05 |
| 8914601 | *nrdH* | glutaredoxin-like protein NrdH | 1.96 | 1.98E-05 |
| 8912853 | *fliE* | flagellar hook-basal body complex protein FliE | -1.96 | 2.55E-05 |
| 8912706 | *rpmH* | 50S ribosomal protein L34 | -1.65 | 2.57E-05 |
| 8913780 | *EAMY_RS32225* | hypothetical protein | 1.76 | 2.65E-05 |
| 8912998 | *gntK* | gluconokinase | -1.61 | 2.87E-05 |
| 23673415 | *EAMY_RS32045* | hypothetical protein | 1.65 | 2.93E-05 |
| 8912888 | *EAMY_RS30860* | LysE family translocator | -1.53 | 3.39E-05 |
| 8911562 | *EAMY_RS24630* | GlsB/YeaQ/YmgE family stress response membrane protein | 2.53 | 3.64E-05 |
| 8911367 | *EAMY_RS21150* | winged helix-turn-helix domain-containing protein | 1.78 | 4.04E-05 |
| 55585556 | *EAMY_RS36340* | hypothetical protein | 3.36 | 4.09E-05 |
| 8911378 | *EAMY_RS21335* | 16S ribosomal RNA | 1.82 | 4.51E-05 |
| 8913269 | *EAMY_RS33720* | siderophore-interacting protein | -2.06 | 4.55E-05 |
| 55585574 | *EAMY_RS36430* | hypothetical protein | 1.79 | 4.69E-05 |
| 8911517 | *lolA* | outer membrane lipoprotein chaperone LolA | 1.65 | 4.69E-05 |
|  | *MSTRG.1720.1* | Unannotated transcript | 2.76 | 5.47E-05 |
| 8912006 | *tagF* | type VI secretion system-associated protein TagF | -2.19 | 5.84E-05 |
| 8913512 | *EAMY_RS28205* | hypothetical protein | -1.70 | 5.93E-05 |
| 8912281 | *EAMY_RS18470* | tRNA-Thr | -1.54 | 6.96E-05 |
| 8912618 | *EAMY_RS19640* | molecular chaperone | 1.89 | 7.35E-05 |
| 8914685 | *zapE* | cell division protein ZapE | -1.82 | 7.48E-05 |
| 8914606 | *cas1e* | type I-E CRISPR-associated endonuclease Cas1e | 1.78 | 7.54E-05 |
| 8913068 | *EAMY_RS32040* | class I SAM-dependent methyltransferase | 1.64 | 8.24E-05 |
| 8913980 | *EAMY_RS24770* | hypothetical protein | 1.70 | 9.13E-05 |
| 8912549 | *punC* | purine nucleoside transporter PunC | -3.90 | 9.33E-05 |
| 8913925 | *secM* | secA translation cis-regulator SecM | -1.67 | 9.99E-05 |
| 8914292 | *EAMY_RS22090* | DMT family transporter | -1.77 | 1.09E-04 |
| 8911276 | *EAMY_RS19630* | spore coat U domain-containing protein | 1.83 | 1.24E-04 |
| 8913742 | *fliR* | flagellar biosynthetic protein FliR | -2.15 | 1.24E-04 |
| 8911990 | *tssF* | type VI secretion system baseplate subunit TssF | -1.79 | 1.36E-04 |
| 43837316 | *EAMY_RS36235* | pseudo | -1.96 | 1.43E-04 |
| 8912478 | *astB* | N-succinylarginine dihydrolase | 4.61 | 1.43E-04 |
| 8913115 | *sctV* | type III secretion system export apparatus subunit SctV | -2.61 | 1.67E-04 |
| 8914542 | *rplY* | 50S ribosomal protein L25 | -2.55 | 1.71E-04 |
|  | *MSTRG.20.1* | Unannotated transcript | 3.03 | 1.76E-04 |
| 23673304 | *EAMY_RS17590* | hypothetical protein | 3.36 | 1.77E-04 |
| 8913133 | *ppiD* | peptidylprolyl isomerase | -1.96 | 1.77E-04 |
| 23673401 | *EAMY_RS31015* | hypothetical protein | 2.08 | 1.94E-04 |
| 8914552 | *EAMY_RS22530* | DUF883 family protein | 1.59 | 2.00E-04 |
| 8914144 | *secE* | preprotein translocase subunit SecE | 2.32 | 2.26E-04 |
| 8912874 | *fliO* | flagellar biosynthetic protein FliO | 1.79 | 2.26E-04 |
| 8914133 | *rho* | transcription termination factor Rho | -1.69 | 2.35E-04 |
| 8912577 | *tssG* | type VI secretion system baseplate subunit TssG | -1.70 | 2.47E-04 |
| 8911413 | *EAMY_RS21725* | PsiF family protein | 1.68 | 2.60E-04 |
| 8914924 | *dolP* | division/outer membrane stress-associated lipid-binding lipoprotein | -4.43 | 2.82E-04 |
| 8912811 | *flgA* | flagellar basal body P-ring formation chaperone FlgA | 2.60 | 2.86E-04 |
| 8913307 | *EAMY_RS29980* | methionine ABC transporter permease MetI | 4.24 | 2.87E-04 |
| 8913647 | *rpsF* | 30S ribosomal protein S6 | -1.54 | 3.19E-04 |
| 8913997 | *sctE* | type III secretion system translocon subunit SctE | 1.90 | 3.76E-04 |
| 69102808 | *kdpF* | K(+)-transporting ATPase subunit F | 3.05 | 3.77E-04 |
| 8912923 | *EAMY_RS23970* | YccJ family protein | 2.76 | 4.00E-04 |
| 8912661 | *EAMY_RS34020* | SemiSWEET family transporter | 2.45 | 4.35E-04 |
|  | *MSTRG.1813.1* | Unannotated transcript | -1.64 | 4.48E-04 |
| 8912761 | *rnc* | ribonuclease III | 1.87 | 4.49E-04 |
| 8912251 | *EAMY_RS18040* | tRNA-Asp | -2.87 | 4.52E-04 |
| 8913774 | *rsxD* | electron transport complex subunit RsxD | -1.82 | 4.52E-04 |
| 8913004 | *rhlB* | ATP-dependent RNA helicase RhlB | -1.98 | 5.41E-04 |
| 8912996 | *hpxA* | allantoin racemase | 1.80 | 5.60E-04 |
| 8912271 | *EAMY_RS18045* | tRNA-Trp | -2.58 | 5.85E-04 |
| 23673375 | *EAMY_RS28080* | hypothetical protein | 2.79 | 6.96E-04 |
| 8912228 | *EAMY_RS34080* | FaeA/PapI family transcriptional regulator | -2.56 | 7.97E-04 |
| 8913494 | *EAMY_RS26315* | ABC transporter ATP-binding protein | -4.94 | 8.73E-04 |
| 8911549 | *EAMY_RS24480* | antiterminator Q family protein | 1.52 | 9.19E-04 |
| 8911702 | *cspE* | transcription antiterminator/RNA stability regulator CspE | -1.67 | 1.07E-03 |
| 69102773 | *EAMY_RS36550* | pseudo | 2.03 | 1.08E-03 |
| 8913248 | *hupB* | nucleoid-associated protein HU-beta | -2.08 | 1.08E-03 |
| 8913926 | *gpsA* | NAD(P)H-dependent glycerol-3-phosphate dehydrogenase | 7.00 | 1.15E-03 |
| 8912335 | *EAMY_RS29965* | 16S ribosomal RNA | 5.66 | 1.20E-03 |
| 8913828 | *rpsH* | 30S ribosomal protein S8 | -2.05 | 1.24E-03 |
| 8913928 | *nusG* | transcription termination/antitermination protein NusG | 4.10 | 1.26E-03 |
| 8914923 | *diaA* | DnaA initiator-associating protein DiaA | -2.41 | 1.62E-03 |
| 8914491 | *EAMY_RS26715* | PqiB family protein | 1.55 | 1.81E-03 |
| 8911246 | *EAMY_RS19245* | restriction endonuclease | 2.70 | 1.98E-03 |
| 8913622 | *cysS* | cysteine--tRNA ligase | -1.98 | 2.11E-03 |
| 8912842 | *EAMY_RS29605* | RNA polymerase sigma factor FliA | 2.20 | 2.38E-03 |
| 8912334 | *EAMY_RS29960* | tRNA-Ile | -1.61 | 2.41E-03 |
| 8913345 | *modC* | molybdenum ABC transporter ATP-binding protein ModC | -1.63 | 2.41E-03 |
| 8912265 | *EAMY_RS32745* | 23S ribosomal RNA | 4.78 | 2.48E-03 |
| 8912344 | *EAMY_RS32755* | 16S ribosomal RNA | 5.24 | 2.68E-03 |
| 23673457 | *EAMY_RS34550* | pseudo | 2.02 | 2.83E-03 |
| 8911262 | *ribB* | 3,4-dihydroxy-2-butanone-4-phosphate synthase | -2.57 | 2.97E-03 |
| 8912953 | *glgB* | 1,4-alpha-glucan branching enzyme | 3.45 | 3.04E-03 |
| 8912795 | *EAMY_RS30200* | hypothetical protein | 1.82 | 3.33E-03 |
| 23673436 | *EAMY_RS33310* | hypothetical protein | 2.01 | 3.66E-03 |
| 23673408 | *EAMY_RS31430* | hypothetical protein | 3.08 | 3.81E-03 |
| 8913509 | *prfC* | peptide chain release factor 3 | -2.46 | 4.02E-03 |
| 69102796 | *EAMY_RS36665* | pseudo | 1.61 | 4.20E-03 |
| 30316869 | *ffs* | ncRNA | 2.47 | 4.20E-03 |
| 8911933 | *cas2e* | type I-E CRISPR-associated endoribonuclease Cas2e | 1.96 | 4.33E-03 |
| 8911995 | *tagH* | type VI secretion system-associated FHA domain protein TagH | -4.36 | 4.73E-03 |
| 8914085 | *thiM* | hydroxyethylthiazole kinase | -1.54 | 5.36E-03 |
| 8913810 | *rsxB* | electron transport complex subunit RsxB | 2.83 | 5.55E-03 |
| 8913002 | *galM* | galactose-1-epimerase | -4.22 | 5.84E-03 |
| 8913857 | *EAMY_RS22155* | type B 50S ribosomal protein L31 | 2.69 | 6.04E-03 |
| 8912970 | *EAMY_RS33315* | hypothetical protein | 1.66 | 6.07E-03 |
| 8911337 | *EAMY_RS20535* | SymE family type I addiction module toxin | 1.74 | 6.93E-03 |
|  | *MSTRG.1589.1* | Unannotated transcript | 2.30 | 7.07E-03 |
| 8913880 | *rplE* | 50S ribosomal protein L5 | -1.60 | 8.25E-03 |
| 8913179 | *EAMY_RS24750* | HrpJ domain-containing protein | 2.00 | 8.38E-03 |
| 23673433 | *yjbE* | exopolysaccharide production protein YjbE | -1.93 | 8.56E-03 |
| 8912504 | *nth* | endonuclease III | 1.50 | 8.60E-03 |
| 8914618 | *cas5e* | type I-E CRISPR-associated protein Cas5/CasD | 1.57 | 9.45E-03 |
| 8912813 | *flgB* | flagellar basal body rod protein FlgB | 1.83 | 1.01E-02 |
| 8913198 | *fkpB* | FKBP-type peptidyl-prolyl cis-trans isomerase | 3.53 | 1.08E-02 |
| 8913908 | *EAMY_RS26095* | hypothetical protein | 1.50 | 1.13E-02 |
| 8911275 | *ftsP* | cell division protein FtsP | 3.72 | 1.14E-02 |
| 41697643 | *EAMY_RS36075* | hypothetical protein | 5.29 | 1.16E-02 |
| 8913297 | *rrf* | 5S ribosomal RNA | 3.30 | 1.20E-02 |
| 8914928 | *mlaB* | lipid asymmetry maintenance protein MlaB | 5.00 | 1.22E-02 |
| 8913216 | *rrf* | 5S ribosomal RNA | 4.97 | 1.23E-02 |
|  | *MSTRG.1016.1* | Unannotated transcript | 1.61 | 1.25E-02 |
| 8913763 | *EAMY_RS30215* | SymE family type I addiction module toxin | 3.91 | 1.26E-02 |
| 8911283 | *EAMY_RS19695* | hypothetical protein | 2.10 | 1.27E-02 |
| 8912763 | *exbD* | TonB system transport protein ExbD | -3.05 | 1.40E-02 |
| 8913991 | *sctT* | type III secretion system export apparatus subunit SctT | 3.17 | 1.44E-02 |
| 8914302 | *purK* | 5-(carboxyamino)imidazole ribonucleotide synthase | -2.12 | 1.45E-02 |
| 8914609 | *EAMY_RS22570* | aldolase | 2.03 | 1.57E-02 |
| 8914029 | *ssuC* | aliphatic sulfonate ABC transporter permease SsuC | 2.22 | 1.65E-02 |
| 8914838 | *EAMY_RS19220* | tRNA-Leu | -1.88 | 1.67E-02 |
| 8913968 | *EAMY_RS29560* | Tar ligand binding domain-containing protein | 1.65 | 1.91E-02 |
| 8912250 | *rrf* | 5S ribosomal RNA | 2.00 | 2.03E-02 |
| 30316910 | *sctF* | pseudo | 3.17 | 2.12E-02 |
| 8912253 | *rrf* | 5S ribosomal RNA | 2.17 | 2.23E-02 |
| 8913510 | *EAMY_RS27310* | hypothetical protein | 1.79 | 2.24E-02 |
|  | *MSTRG.2542.1* | Unannotated transcript | -2.50 | 2.25E-02 |
| 23673312 | *dptH* | pseudo | 2.75 | 2.34E-02 |
| 8913672 | *pspF* | phage shock protein operon transcriptional activator | 1.59 | 2.35E-02 |
| 8914620 | *casB* | type I-E CRISPR-associated protein Cse2/CasB | 2.58 | 2.39E-02 |
| 23673300 | *EAMY_RS17570* | hypothetical protein | 1.67 | 2.39E-02 |
| 8912256 | *rrf* | 5S ribosomal RNA | 3.38 | 2.41E-02 |
| 8912247 | *rrf* | 5S ribosomal RNA | 2.78 | 2.45E-02 |
| 8912401 | *EAMY_RS22025* | lipoprotein | -1.54 | 2.69E-02 |
| 8913636 | *prmC* | peptide chain release factor N(5)-glutamine methyltransferase | 2.69 | 2.75E-02 |
| 8913233 | *thyA* | thymidylate synthase | 3.25 | 2.82E-02 |
| 8913788 | *cfa* | cyclopropane fatty acyl phospholipid synthase | 2.83 | 2.94E-02 |
| 8914072 | *tauC* | taurine ABC transporter permease TauC | 1.69 | 3.34E-02 |
| 8914224 | *yigB* | 5-amino-6-(5-phospho-D-ribitylamino)uracil phosphatase YigB | 2.35 | 3.45E-02 |
| 8913263 | *poxB* | ubiquinone-dependent pyruvate dehydrogenase | 3.05 | 3.46E-02 |
| 8913631 | *pqqB* | pyrroloquinoline quinone biosynthesis protein PqqB | -2.74 | 3.65E-02 |
| 8912080 | *EAMY_RS32100* | hypothetical protein | 2.12 | 4.02E-02 |
|  | *MSTRG.1888.1* | Unannotated transcript | 1.53 | 4.06E-02 |
|  | *MSTRG.791.1* | Unannotated transcript | 1.70 | 4.31E-02 |

^a^ Gene IDs, locus tags, symbols and functions obtained from the DAVID database for *E. amylovora* CFBP 1430. MSTRG identifiers represent assembled and annotated by StringTie, which were not previously annotated in CFBP 1430 strain. They could be potentially new transcripts, alternative splice variants, or transcripts specific to the experimental conditions.

**Table S2.** DEGs in EaR2 stationary-phase cells compared to Ea273^a^

| **Gene ID** | **Symbol** | **Function** | **log2FC** | **FDR** |
| --- | --- | --- | --- | --- |
| 8913482 | EAMY_RS22460 | ABC transporter permease | 2.48 | 1.23E-16 |
| 8913594 | EAMY_RS31375 | nucleoside hydrolase | -2.47 | 3.68E-16 |
| 8912922 | aspA | aspartate ammonia-lyase | 2.57 | 3.68E-16 |
| 8914474 | gapA | glyceraldehyde-3-phosphate dehydrogenase | -2.57 | 6.22E-16 |
| 8912325 | EAMY_RS27460 | Lrp/AsnC family transcriptional regulator | 2.72 | 5.04E-14 |
| 23673361 | EAMY_RS25875 | DapH/DapD/GlmU-related protein | 7.61 | 7.28E-14 |
| 8913483 | EAMY_RS22465 | ABC transporter ATP-binding protein | 1.99 | 7.32E-13 |
| 8913261 | EAMY_RS34315 | phage holin, lambda family | 2.73 | 7.77E-13 |
| 8911654 | EAMY_RS25865 | hypothetical protein | 7.11 | 1.30E-12 |
| 8912822 | EAMY_RS24220 | flagellar basal body P-ring protein FlgI | -2.65 | 2.71E-12 |
| 8914507 | EAMY_RS24385 | M15 family metallopeptidase | 2.35 | 2.99E-12 |
| 8914013 | EAMY_RS25670 | FAD-binding protein | 1.91 | 3.51E-12 |
| 8912326 | EAMY_RS28070 | DUF3320 domain-containing protein | 2.01 | 3.56E-12 |
| 8912814 | flgE | flagellar hook protein FlgE | -4.42 | 5.74E-12 |
| 8912816 | EAMY_RS24205 | flagellar basal body rod protein FlgF | -4.42 | 5.79E-12 |
| 8912818 | flgG | flagellar basal-body rod protein FlgG | -3.77 | 1.46E-11 |
| 8912812 | flgD | flagellar hook assembly protein FlgD | -4.56 | 1.72E-11 |
| 8914385 | EAMY_RS24120 | cytochrome b | 2.06 | 1.83E-11 |
| 8912857 | fliG | flagellar motor switch protein FliG | -4.13 | 1.97E-11 |
| 8913753 | EAMY_RS20730 | DUF1440 domain-containing protein | -2.52 | 2.86E-11 |
| 8912563 | EAMY_RS26950 | chemotaxis response regulator protein-glutamate methylesterase | -1.69 | 4.36E-11 |
| 43500273 | EAMY_RS36170 | hypothetical protein | 5.71 | 6.96E-11 |
| 8913685 | EAMY_RS34310 | glycoside hydrolase family 68 protein | 2.01 | 9.65E-11 |
| 8911735 | flhC | flagellar transcriptional regulator FlhC | 1.74 | 1.48E-10 |
| 8912808 | flgB | flagellar basal body rod protein FlgB | -5.41 | 1.52E-10 |
| 8912861 | fliI | flagellar protein export ATPase FliI | -3.57 | 2.00E-10 |
| 8912843 | flhA | flagellar biosynthesis protein FlhA | -2.95 | 2.29E-10 |
| 8911655 | EAMY_RS25880 | hypothetical protein | 5.10 | 2.54E-10 |
| 8912832 | flgA | flagellar basal body P-ring formation chaperone FlgA | -2.01 | 4.68E-10 |
| 8912810 | flgC | flagellar basal body rod protein FlgC | -4.77 | 7.96E-10 |
| 8912070 | EAMY_RS32050 | ATP-grasp domain-containing protein | 2.34 | 1.22E-09 |
| 8912847 | fliD | flagellar filament capping protein FliD | -2.45 | 1.42E-09 |
| 8912590 | EAMY_RS19755 | PAS and helix-turn-helix domain-containing protein | 1.53 | 1.63E-09 |
| 8912865 | EAMY_RS24435 | flagellar hook-length control protein FliK | -2.76 | 1.83E-09 |
| 8914506 | fliZ | flagella biosynthesis regulatory protein FliZ | -2.84 | 2.81E-09 |
| 8911816 | EAMY_RS28335 | ADP-ribosyltransferase | 3.11 | 6.41E-09 |
| 31493057 | EAMY_RS35740 | hypothetical protein | -1.73 | 7.37E-09 |
| 8912883 | EAMY_RS27170 | RNA polymerase sigma factor FliA | -3.18 | 9.97E-09 |
| 8912871 | fliN | flagellar motor switch protein FliN | -2.60 | 1.82E-08 |
| 8912648 | cysC | adenylyl-sulfate kinase | -3.29 | 2.77E-08 |
| 8912570 | EAMY_RS19625 | SDR family oxidoreductase | 2.38 | 2.88E-08 |
| 30316919 | EAMY_RS35020 | hypothetical protein | 5.09 | 2.93E-08 |
| 8912735 | EAMY_RS20035 | LysR family transcriptional regulator | 1.75 | 5.40E-08 |
| 30317028 | EAMY_RS35565 | ogr/Delta-like zinc finger family protein | 2.12 | 7.48E-08 |
| 8912959 | dapD | 2,3,4,5-tetrahydropyridine-2,6-dicarboxylate N-succinyltransferase | -1.82 | 8.16E-08 |
| 8912859 | fliH | flagellar assembly protein FliH | -3.22 | 1.06E-07 |
| 8912639 | cysN | sulfate adenylyltransferase subunit CysN | -3.45 | 2.20E-07 |
| 8911286 | EAMY_RS19760 | hypothetical protein | 1.93 | 4.12E-07 |
| 8911600 | araA | L-arabinose isomerase | 2.76 | 5.17E-07 |
| 8912820 | EAMY_RS24215 | flagellar basal body L-ring protein FlgH | -2.94 | 5.37E-07 |
| 8914861 | EAMY_RS25825 | hypothetical protein | 1.80 | 6.26E-07 |
| 55585554 | EAMY_RS36330 | hypothetical protein | -1.89 | 6.28E-07 |
| 8911752 | EAMY_RS27180 | flagellin | -2.30 | 1.04E-06 |
| 8913118 | hrpN | type III secretion system harpin HrpN | -1.75 | 1.54E-06 |
| 8911776 | EAMY_RS27465 | Dabb family protein | 2.01 | 1.90E-06 |
| 8912826 | flgK | flagellar hook-associated protein FlgK | -1.75 | 3.25E-06 |
| 8912867 | fliL | flagellar basal body-associated protein FliL | -4.29 | 3.25E-06 |
| 8912647 | cysA | sulfate/thiosulfate ABC transporter ATP-binding protein CysA | -1.69 | 6.53E-06 |
| 8911358 | cysG | siroheme synthase CysG | -2.14 | 7.12E-06 |
| 8911801 | EAMY_RS28075 | restriction endonuclease | 2.49 | 9.26E-06 |
| 8913504 | EAMY_RS19950 | Hrp pili protein HrpA | -3.15 | 1.07E-05 |
| 8912875 | fliP | flagellar type III secretion system pore protein FliP | -2.21 | 1.14E-05 |
| 8914629 | EAMY_RS24695 | YgdI/YgdR family lipoprotein | 1.54 | 1.52E-05 |
| 55585556 | EAMY_RS36340 | hypothetical protein | 3.92 | 1.68E-05 |
| 8913262 | ileS | isoleucine--tRNA ligase | -5.47 | 2.10E-05 |
| 8912835 | flhB | flagellar biosynthesis protein FlhB | -5.05 | 2.16E-05 |
| 8912652 | EAMY_RS28880 | sulfate ABC transporter substrate-binding protein | -1.79 | 2.60E-05 |
| 8912853 | fliE | flagellar hook-basal body complex protein FliE | -2.45 | 2.75E-05 |
| 8912641 | cysD | sulfate adenylyltransferase subunit CysD | -2.73 | 3.51E-05 |
| 8913093 | hrpT | HrpT family type III secretion system protein | -2.57 | 3.74E-05 |
| 8913570 | EAMY_RS17820 | sulfate ABC transporter substrate-binding protein | -2.56 | 3.78E-05 |
| 8913908 | EAMY_RS26095 | hypothetical protein | 2.36 | 5.03E-05 |
| 8912015 | EAMY_RS31365 | DUF2946 family protein | 1.97 | 5.87E-05 |
| 30316974 | EAMY_RS35295 | pseudo | -2.86 | 6.25E-05 |
| 8912654 | cysK | cysteine synthase A | -1.57 | 6.99E-05 |
| 8913101 | EAMY_RS19955 | EscI/YscI/HrpB family type III secretion system inner rod protein | -1.63 | 7.17E-05 |
| 8913661 | proX | glycine betaine/L-proline ABC transporter substrate-binding protein ProX | -2.56 | 8.48E-05 |
| 8914428 | tpx | thiol peroxidase | -1.56 | 1.28E-04 |
| 8913512 | EAMY_RS28205 | hypothetical protein | -1.72 | 3.34E-04 |
| 8911777 | EAMY_RS27470 | DUF2000 domain-containing protein | 1.83 | 4.39E-04 |
| 43837316 | EAMY_RS36235 | pseudo | -2.89 | 6.74E-04 |
| 8912016 | EAMY_RS31370 | PepSY-associated TM helix domain-containing protein | 1.85 | 8.11E-04 |
| 8912873 | fliO | flagellar biosynthetic protein FliO | -3.07 | 1.03E-03 |
| 8913660 | proW | glycine betaine/L-proline ABC transporter permease ProW | -1.68 | 2.41E-03 |
| 8912855 | fliF | flagellar basal-body MS-ring/collar protein FliF | -2.67 | 2.86E-03 |
| 8913386 | EAMY_RS18960 | helix-turn-helix domain-containing protein | -1.59 | 3.16E-03 |
| 8913297 | rrf | 5S ribosomal RNA | 3.65 | 3.60E-03 |
| 30316860 | ssrS | ncRNA | -2.26 | 3.82E-03 |
| 69102796 | EAMY_RS36665 | pseudo | 1.65 | 4.42E-03 |
| 8911562 | EAMY_RS24630 | GlsB/YeaQ/YmgE family stress response membrane protein | 1.51 | 6.35E-03 |
| 8913905 | sapC | putrescine export ABC transporter permease SapC | 1.76 | 8.13E-03 |
| 23673375 | EAMY_RS28080 | hypothetical protein | 3.03 | 9.77E-03 |
| 8912253 | rrf | 5S ribosomal RNA | 2.26 | 1.35E-02 |
| 43837313 | EAMY_RS36220 | hypothetical protein | 1.62 | 1.62E-02 |
| 69102802 | EAMY_RS36695 | hypothetical protein | -1.86 | 1.69E-02 |
| 8912250 | rrf | 5S ribosomal RNA | 2.07 | 1.71E-02 |
| 8911573 | EAMY_RS24825 | acyl carrier protein | 2.49 | 1.71E-02 |
| 8914069 | tauA | taurine ABC transporter substrate-binding protein | -3.24 | 2.02E-02 |
| 8913216 | rrf | 5S ribosomal RNA | 4.32 | 2.18E-02 |
| 8914070 | tauB | taurine ABC transporter ATP-binding subunit | -1.98 | 2.40E-02 |
| 8912763 | exbD | TonB system transport protein ExbD | -4.39 | 2.57E-02 |
| 69102808 | kdpF | K(+)-transporting ATPase subunit F | 2.28 | 2.70E-02 |
| 8912247 | rrf | 5S ribosomal RNA | 2.35 | 3.79E-02 |
| 8912256 | rrf | 5S ribosomal RNA | 2.56 | 4.07E-02 |
| 8913993 | EAMY_RS24800 | EscU/YscU/HrcU family type III secretion system export apparatus switch protein | 2.93 | 4.75E-02 |
| 8912858 | fliG | flagellar motor switch protein FliG | 1.58 | 4.96E-02 |
| 23673433 | yjbE | exopolysaccharide production protein YjbE | -1.87 | 5.28E-02 |
| 23673427 | EAMY_RS32590 | pseudo | 1.73 | 5.44E-02 |
| 8911514 | ltaE | low-specificity L-threonine aldolase | 2.67 | 6.74E-02 |
| 8912154 | tauD | taurine dioxygenase | -1.56 | 7.25E-02 |
| 8913494 | EAMY_RS26315 | ABC transporter ATP-binding protein | -2.76 | 7.91E-02 |
| 8913990 | sctT | type III secretion system export apparatus subunit SctT | 2.44 | 8.00E-02 |
| 8912953 | glgB | 1,4-alpha-glucan branching enzyme | 2.25 | 8.30E-02 |
| 8913106 | EAMY_RS19975 | HPr kinase | -1.98 | 9.57E-02 |
| 8913999 | EAMY_RS20255 | cysteine peptidase family C39 domain-containing protein | 2.07 | 1.03E-01 |
| 8914123 | trpA | tryptophan synthase subunit alpha | -1.71 | 1.05E-01 |
| 8911991 | tssE | type VI secretion system baseplate subunit TssE | -1.82 | 1.07E-01 |
| 8913926 | gpsA | NAD(P)H-dependent glycerol-3-phosphate dehydrogenase | 2.99 | 1.22E-01 |
| 8912229 | EAMY_RS34085 | winged helix-turn-helix domain-containing protein | 2.05 | 1.22E-01 |
| 8912228 | EAMY_RS34080 | FaeA/PapI family transcriptional regulator | -2.07 | 1.41E-01 |
| 8912824 | flgJ | flagellar assembly peptidoglycan hydrolase FlgJ | -2.46 | 1.41E-01 |
| 8913991 | sctT | type III secretion system export apparatus subunit SctT | 2.16 | 1.46E-01 |

^a^ Gene IDs, locus tags, symbols and functions obtained from the DAVID database for *E. amylovora* CFBP 1430.

**Table S3.** DEGs in the copper tolerant strain Ea273 in response to different copper treatments^a,b^.

| **Treatment** | **Gene ID** | **Gene symbol** | **Gene Function** | **Log2FC** | **FDR** |
| --- | --- | --- | --- | --- | --- |
| **Copper Shock (LS1-L)** | 8914287 | *copA* | copper-exporting P-type ATPase CopA | 5.46 | 3.62E-20 |
|  | 8911929 | *cueO* | multicopper oxidase CueO | 5.54 | 1.85E-15 |
|  | 8914283 | *EAMY_RS22290* | TraB/GumN family protein | 3.24 | 5.48E-14 |
|  | 8912322 | *katG* | catalase/peroxidase HPI | 3.90 | 6.16E-13 |
|  | 8914722 | *EAMY_RS33475* | zinc/cadmium/mercury/lead-transporting ATPase | 3.29 | 6.14E-12 |
|  | 8912441 | *EAMY_RS17985* | glutathione peroxidase | 6.77 | 7.73E-12 |
|  | 8911440 | *ybaK* | Cys-tRNA(Pro)/Cys-tRNA(Cys) deacylase YbaK | 1.77 | 1.18E-10 |
|  | 8913976 | *soxS* | superoxide response transcriptional regulator SoxS | 2.71 | 4.94E-10 |
|  | 8911634 | *EAMY_RS25705* | helix-turn-helix domain-containing protein | 3.05 | 5.09E-10 |
|  | 8912892 | *panB* | 3-methyl-2-oxobutanoate hydroxymethyltransferase | -1.91 | 3.47E-09 |
|  | 8912479 | *spy* | ATP-independent periplasmic protein-refolding chaperone Spy | 1.98 | 3.94E-09 |
|  | 23673387 | *EAMY_RS28935* | DUF1471 domain-containing protein | 4.49 | 5.50E-08 |
|  | 8912016 | *EAMY_RS31370* | PepSY-associated TM helix domain-containing protein | -2.57 | 7.82E-08 |
|  | 8914591 | *trxC* | thioredoxin TrxC | 1.77 | 9.11E-07 |
|  | 8911927 | *mrcB* | bifunctional glycosyl transferase/transpeptidase | -1.86 | 9.11E-07 |
|  | 8914348 | *EAMY_RS23505* | GrxA family glutaredoxin | 1.85 | 2.08E-06 |
|  | 8913535 | *panC* | pantoate--beta-alanine ligase | -1.84 | 2.30E-06 |
|  | 8913363 | *hrpB* | ATP-dependent helicase HrpB | -1.59 | 3.64E-06 |
|  | 8912703 | *gluQRS* | tRNA glutamyl-Q(34) synthetase GluQRS | -2.48 | 5.15E-06 |
|  | 69102774 | *EAMY_RS36555* | hypothetical protein | 2.12 | 1.06E-05 |
|  | 8913134 | *EAMY_RS23320* | HutD family protein | -1.79 | 1.58E-05 |
|  | 8914242 | *hpt* | hypoxanthine phosphoribosyltransferase | -1.90 | 5.50E-05 |
|  | 8914241 | *pcnB* | polynucleotide adenylyltransferase PcnB | -1.75 | 6.56E-05 |
|  | 8912596 | *cpxR* | envelope stress response regulator transcription factor CpxR | 1.77 | 3.82E-04 |
|  | 8913536 | *panD* | aspartate 1-decarboxylase | -1.60 | 6.13E-03 |
|  | 8914924 | *dolP* | division/outer membrane stress-associated lipid-binding lipoprotein | 1.66 | 1.56E-02 |
|  | 8913345 | *modC* | molybdenum ABC transporter ATP-binding protein ModC | -1.52 | 3.44E-02 |
|  | 69102773 | *EAMY_RS36550* | pseudo | 1.77 | 3.84E-02 |
| **Copper Adaptation (LA1-L)** | 8914491 | *EAMY_RS26715* | PqiB family protein | 4.74 | 1.41E-18 |
|  | 8913053 | *hutU* | urocanate hydratase | 5.25 | 7.47E-15 |
|  | 8914442 | *EAMY_RS19660* | YlaC family protein | 2.51 | 4.11E-11 |
|  | 8913139 | *hutH* | histidine ammonia-lyase | -4.11 | 1.74E-09 |
|  | 8913134 | *EAMY_RS23320* | HutD family protein | -1.69 | 4.71E-09 |
|  | 8914242 | *hpt* | hypoxanthine phosphoribosyltransferase | -1.72 | 7.62E-07 |
|  | 8913135 | *hutI* | imidazolonepropionase | -2.20 | 2.26E-06 |
|  | 8914287 | *copA* | copper-exporting P-type ATPase CopA | -1.90 | 4.02E-06 |
|  | 8913345 | *modC* | molybdenum ABC transporter ATP-binding protein ModC | -1.94 | 6.17E-06 |
|  | 8914283 | *EAMY_RS22290* | TraB/GumN family protein | 1.55 | 2.86E-05 |
|  | 8914924 | *dolP* | division/outer membrane stress-associated lipid-binding lipoprotein | -1.57 | 8.21E-04 |
|  | 8914722 | *EAMY_RS33475* | zinc/cadmium/mercury/lead-transporting ATPase | 1.87 | 7.25E-03 |
|  | 8911929 | *cueO* | multicopper oxidase CueO | -5.51 | 7.87E-03 |
|  | 8914036 | *sucD* | succinate--CoA ligase subunit alpha | 1.72 | 9.84E-03 |
|  | 23673387 | *EAMY_RS28935* | DUF1471 domain-containing protein | -1.68 | 2.99E-02 |
|  | 8912016 | *EAMY_RS31370* | PepSY-associated TM helix domain-containing protein | 1.79 | 3.65E-02 |
| **Copper Adaptation (SA1-S)** | 8914287 | *copA* | copper-exporting P-type ATPase CopA | 5.07 | 2.41E-19 |
|  | 8914283 | *EAMY_RS22290* | TraB/GumN family protein | 3.57 | 1.13E-15 |
|  | 8911929 | *cueO* | multicopper oxidase CueO | 4.90 | 1.52E-14 |
|  | 8914442 | *EAMY_RS19660* | YlaC family protein | -3.21 | 5.83E-14 |
|  | 8911440 | *ybaK* | Cys-tRNA(Pro)/Cys-tRNA(Cys) deacylase YbaK | 2.30 | 7.02E-14 |
|  | 8913538 | *EAMY_RS30290* | ABC transporter permease | 1.50 | 1.56E-13 |
|  | 8914242 | *hpt* | hypoxanthine phosphoribosyltransferase | -2.53 | 1.39E-09 |
|  | 8912636 | *cysW* | sulfate/thiosulfate ABC transporter permease CysW | 1.59 | 2.19E-07 |
|  | 8914722 | *EAMY_RS33475* | zinc/cadmium/mercury/lead-transporting ATPase | 1.98 | 2.92E-07 |
|  | 8913940 | *dksA* | RNA polymerase-binding protein DksA | -1.89 | 8.36E-07 |
|  | 8913363 | *hrpB* | ATP-dependent helicase HrpB | -1.84 | 1.45E-06 |
|  | 8913092 | *EAMY_RS30310* | cystathionine gamma-synthase family protein | -1.84 | 8.68E-06 |
|  | 8913783 | *cbl* | HTH-type transcriptional regulator Cbl | 1.97 | 3.27E-05 |
|  | 8911927 | *mrcB* | bifunctional glycosyl transferase/transpeptidase | -1.57 | 2.01E-04 |
|  | 8914070 | *tauB* | taurine ABC transporter ATP-binding subunit | 2.22 | 3.02E-04 |
|  | 23673399 | *can* | carbonate dehydratase | -2.08 | 5.63E-04 |
|  | 8912641 | *cysD* | sulfate adenylyltransferase subunit CysD | 1.78 | 9.20E-04 |
|  | 8914861 | *EAMY_RS25825* | hypothetical protein | -1.58 | 1.03E-03 |
|  | 8912892 | *panB* | 3-methyl-2-oxobutanoate hydroxymethyltransferase | -1.80 | 2.42E-03 |
|  | 8913504 | *EAMY_RS19950* | Hrp pili protein HrpA | -1.51 | 1.23E-02 |
|  | 8914241 | *pcnB* | polynucleotide adenylyltransferase PcnB | -1.79 | 1.23E-02 |
|  | 8913570 | *EAMY_RS17820* | sulfate ABC transporter substrate-binding protein | 1.50 | 2.03E-02 |
|  | 8914246 | *sfsA* | DNA/RNA nuclease SfsA | -2.48 | 2.20E-02 |
|  | 8912016 | *EAMY_RS31370* | PepSY-associated TM helix domain-containing protein | 1.55 | 2.20E-02 |
|  | 8913536 | *panD* | aspartate 1-decarboxylase | -2.33 | 2.64E-02 |
|  | 8913104 | *thpR* | RNA 2',3'-cyclic phosphodiesterase | -1.71 | 4.50E-02 |
|  | 8913535 | *panC* | pantoate--beta-alanine ligase | -1.70 | 4.55E-02 |
|  | 8912228 | *EAMY_RS34080* | FaeA/PapI family transcriptional regulator | -3.52 | 4.55E-02 |
|  | 8911361 | *EAMY_RS21000* | lytic transglycosylase domain-containing protein | 2.15 | 4.98E-02 |
| **Copper Adaptation (SA3-S)** | 8912732 | *EAMY_RS20195* | peroxiredoxin | 4.15 | 6.91E-19 |
|  | 8914287 | *copA* | copper-exporting P-type ATPase CopA | 4.72 | 7.29E-19 |
|  | 8913942 | *EAMY_RS20190* | thioredoxin family protein | 3.89 | 3.78E-18 |
|  | 8914651 | *EAMY_RS20185* | sigma-70 family RNA polymerase sigma factor | 3.70 | 1.16E-16 |
|  | 8911929 | *cueO* | multicopper oxidase CueO | 5.02 | 4.90E-15 |
|  | 8914442 | *EAMY_RS19660* | YlaC family protein | -3.43 | 2.09E-14 |
|  | 8914283 | *EAMY_RS22290* | TraB/GumN family protein | 2.50 | 3.37E-12 |
|  | 8913685 | *EAMY_RS34310* | glycoside hydrolase family 68 protein | -2.46 | 6.73E-12 |
|  | 8914013 | *EAMY_RS25670* | FAD-binding protein | -1.82 | 1.38E-11 |
|  | 8911634 | *EAMY_RS25705* | helix-turn-helix domain-containing protein | 4.07 | 1.81E-11 |
|  | 8912558 | *EAMY_RS26960* | methyl-accepting chemotaxis protein | -1.96 | 5.48E-11 |
|  | 8912563 | *EAMY_RS26950* | chemotaxis response regulator protein-glutamate methylesterase | -1.50 | 2.96E-10 |
|  | 8911440 | *ybaK* | Cys-tRNA(Pro)/Cys-tRNA(Cys) deacylase YbaK | 1.60 | 3.84E-10 |
|  | 8913261 | *EAMY_RS34315* | phage holin, lambda family | -3.97 | 1.90E-09 |
|  | 8912128 | *EAMY_RS32395* | PAS domain-containing methyl-accepting chemotaxis protein | -2.05 | 2.44E-09 |
|  | 8914242 | *hpt* | hypoxanthine phosphoribosyltransferase | -2.31 | 3.17E-09 |
|  | 8912554 | *motB* | flagellar motor protein MotB | -2.08 | 1.36E-08 |
|  | 8911459 | *EAMY_RS22470* | leucine-rich repeat domain-containing protein | -2.09 | 1.41E-08 |
|  | 8912929 | *metK* | methionine adenosyltransferase | -1.55 | 2.20E-08 |
|  | 8914722 | *EAMY_RS33475* | zinc/cadmium/mercury/lead-transporting ATPase | 2.15 | 2.20E-08 |
|  | 8911266 | *EAMY_RS19510* | nucleoside 2-deoxyribosyltransferase | -1.60 | 2.46E-08 |
|  | 8912479 | *spy* | ATP-independent periplasmic protein-refolding chaperone Spy | 1.74 | 3.01E-08 |
|  | 8911612 | *EAMY_RS25565* | hypothetical protein | -1.81 | 8.11E-08 |
|  | 8911752 | *EAMY_RS27180* | flagellin | -2.76 | 8.34E-08 |
|  | 8913497 | *EAMY_RS20015* | HrpW-specific chaperone | -1.51 | 1.77E-07 |
|  | 8911546 | *rcsA* | transcriptional regulator RcsA | 2.06 | 2.43E-07 |
|  | 8912587 | *ybaK* | Cys-tRNA(Pro)/Cys-tRNA(Cys) deacylase YbaK | 1.81 | 3.22E-07 |
|  | 8912181 | *EAMY_RS33570* | FAD-binding oxidoreductase | 1.53 | 3.76E-07 |
|  | 8912847 | *fliD* | flagellar filament capping protein FliD | -1.66 | 4.73E-07 |
|  | 8912151 | *metA* | homoserine O-succinyltransferase | -1.66 | 5.24E-07 |
|  | 30317028 | *EAMY_RS35565* | ogr/Delta-like zinc finger family protein | -3.64 | 7.48E-07 |
|  | 8912849 | *fliS* | flagellar export chaperone FliS | -1.52 | 3.19E-06 |
|  | 8913940 | *dksA* | RNA polymerase-binding protein DksA | -1.57 | 5.12E-06 |
|  | 8913118 | *hrpN* | type III secretion system harpin HrpN | -1.61 | 6.56E-06 |
|  | 8912565 | *cheY* | chemotaxis response regulator CheY | -1.92 | 1.05E-05 |
|  | 8913783 | *cbl* | HTH-type transcriptional regulator Cbl | 1.92 | 1.65E-05 |
|  | 8914507 | *EAMY_RS24385* | M15 family metallopeptidase | -1.56 | 1.80E-05 |
|  | 8911927 | *mrcB* | bifunctional glycosyl transferase/transpeptidase | -1.73 | 1.92E-05 |
|  | 8913101 | *EAMY_RS19955* | EscI/YscI/HrpB family type III secretion system inner rod protein | -1.77 | 2.10E-05 |
|  | 8914861 | *EAMY_RS25825* | hypothetical protein | -2.01 | 3.08E-05 |
|  | 8912883 | *EAMY_RS27170* | RNA polymerase sigma factor FliA | -1.57 | 4.13E-05 |
|  | 23673387 | *EAMY_RS28935* | DUF1471 domain-containing protein | 2.33 | 4.13E-05 |
|  | 8912735 | *EAMY_RS20035* | LysR family transcriptional regulator | -1.60 | 4.45E-05 |
|  | 8912016 | *EAMY_RS31370* | PepSY-associated TM helix domain-containing protein | 2.18 | 1.01E-04 |
|  | 8913504 | *EAMY_RS19950* | Hrp pili protein HrpA | -2.23 | 1.07E-04 |
|  | 8914070 | *tauB* | taurine ABC transporter ATP-binding subunit | 2.12 | 1.62E-04 |
|  | 8913067 | *EAMY_RS32030* | 2-dehydropantoate 2-reductase N-terminal domain-containing protein | -1.61 | 2.52E-04 |
|  | 8913450 | *nuoE* | NADH-quinone oxidoreductase subunit NuoE | 3.37 | 6.00E-04 |
|  | 8911644 | *EAMY_RS25785* | acyltransferase | -1.56 | 6.94E-04 |
|  | 8913068 | *EAMY_RS32040* | class I SAM-dependent methyltransferase | -1.58 | 9.00E-04 |
|  | 8914606 | *cas1e* | type I-E CRISPR-associated endonuclease Cas1e | -1.75 | 1.25E-03 |
|  | 8912835 | *flhB* | flagellar biosynthesis protein FlhB | -2.00 | 1.63E-03 |
|  | 8912218 | *EAMY_RS33980* | hypothetical protein | -1.63 | 3.23E-03 |
|  | 8912086 | *tssF* | type VI secretion system baseplate subunit TssF | -1.71 | 1.95E-02 |
|  | 8912504 | *nth* | endonuclease III | -2.03 | 2.21E-02 |
|  | 8912795 | *EAMY_RS30200* | hypothetical protein | -2.00 | 2.33E-02 |
|  | 8912855 | *fliF* | flagellar basal-body MS-ring/collar protein FliF | -1.81 | 2.64E-02 |
|  | 8912203 | *EAMY_RS33785* | DUF1471 domain-containing protein | 2.00 | 3.45E-02 |
|  | 8913932 | *rplO* | 50S ribosomal protein L15 | -1.86 | 3.69E-02 |
|  | 8912998 | *gntK* | gluconokinase | 1.77 | 4.98E-02 |

^a^ Gene IDs, locus tags, symbols and functions obtained from the DAVID database for *E. amylovora* CFBP 1430.

**Table S4.** DEGs in the copper sensitive strain EaR2 in response to different copper treatments^a^.

| **Treatment** | **Gene ID** | **Gene symbol** | **Gene Function** | **Log_2_FC** | **FDR** |
| --- | --- | --- | --- | --- | --- |
| **Copper Shock (LS1-L)** | 8914287 | *copA* | copper-exporting P-type ATPase CopA | 5.01 | 3.02E-19 |
|  | 8911929 | *cueO* | multicopper oxidase CueO | 5.55 | 1.44E-15 |
|  | 8914283 | *EAMY_RS22290* | TraB/GumN family protein | 3.32 | 6.11E-14 |
|  | 8914722 | *EAMY_RS33475* | zinc/cadmium/mercury/lead-transporting ATPase | 3.56 | 9.32E-13 |
|  | 8912322 | *katG* | catalase/peroxidase HPI | 3.13 | 6.39E-10 |
|  | 8911440 | *ybaK* | Cys-tRNA(Pro)/Cys-tRNA(Cys) deacylase YbaK | 1.69 | 6.83E-10 |
|  | 8912479 | *spy* | ATP-independent periplasmic protein-refolding chaperone Spy | 2.02 | 3.18E-09 |
|  | 8914242 | *hpt* | hypoxanthine phosphoribosyltransferase | -2.84 | 4.58E-09 |
|  | 8913940 | *dksA* | RNA polymerase-binding protein DksA | -2.22 | 9.91E-09 |
|  | 8913976 | *soxS* | superoxide response transcriptional regulator SoxS | 2.23 | 1.76E-08 |
|  | 8911634 | *EAMY_RS25705* | helix-turn-helix domain-containing protein | 2.35 | 1.02E-07 |
|  | 8914442 | *EAMY_RS19660* | YlaC family protein | -1.60 | 1.95E-06 |
|  | 8912441 | *EAMY_RS17985* | glutathione peroxidase | 4.98 | 2.70E-06 |
|  | 23673387 | *EAMY_RS28935* | DUF1471 domain-containing protein | 4.02 | 5.10E-06 |
|  | 8913092 | *EAMY_RS30310* | cystathionine gamma-synthase family protein | -1.97 | 5.10E-06 |
|  | 8913363 | *hrpB* | ATP-dependent helicase HrpB | -1.92 | 1.52E-05 |
|  | 8912892 | *panB* | 3-methyl-2-oxobutanoate hydroxymethyltransferase | -1.94 | 1.87E-04 |
|  | 8911927 | *mrcB* | bifunctional glycosyl transferase/transpeptidase | -1.64 | 2.94E-04 |
|  | 8912703 | *gluQRS* | tRNA glutamyl-Q(34) synthetase GluQRS | -2.52 | 1.81E-02 |
|  | 8914241 | *pcnB* | polynucleotide adenylyltransferase PcnB | -1.88 | 2.72E-02 |
| **Copper Adaptation (LA1-L)** | 8912479 | *spy* | ATP-independent periplasmic protein-refolding chaperone Spy | 6.23 | 1.13E-20 |
|  | 8912357 | *EAMY_RS31520* | SulP family inorganic anion transporter | 3.33 | 7.26E-18 |
|  | 8914287 | *copA* | copper-exporting P-type ATPase CopA | 4.04 | 2.48E-17 |
|  | 8913418 | *EAMY_RS24365* | glycine zipper 2TM domain-containing protein | 2.88 | 5.69E-16 |
|  | 8912412 | *wbaP* | undecaprenyl-phosphate galactose phosphotransferase WbaP | 2.57 | 1.25E-15 |
|  | 8911929 | *cueO* | multicopper oxidase CueO | 5.17 | 2.73E-15 |
|  | 8912406 | *EAMY_RS27705* | polysaccharide biosynthesis tyrosine autokinase | 2.01 | 1.75E-14 |
|  | 8911811 | *kup* | low affinity potassium transporter Kup | -2.84 | 1.75E-14 |
|  | 8914129 | *sltY* | murein transglycosylase | 2.03 | 3.93E-14 |
|  | 8913587 | *efeB* | iron uptake transporter deferrochelatase/peroxidase subunit | -2.12 | 5.30E-14 |
|  | 8914077 | *ybbA* | putative ABC transporter ATP-binding protein YbbA | 2.92 | 6.22E-14 |
|  | 8912404 | *ampC* | class C beta-lactamase | 2.59 | 8.76E-14 |
|  | 8914661 | *EAMY_RS19450* | glutathionylspermidine synthase family protein | 2.60 | 1.08E-13 |
|  | 8914463 | *ppsA* | phosphoenolpyruvate synthase | 2.49 | 1.44E-13 |
|  | 8912414 | *EAMY_RS27680* | phage tailspike protein | 1.75 | 3.03E-13 |
|  | 8911807 | *ompC* | porin OmpC | 2.27 | 2.07E-12 |
|  | 8912472 | *ompC* | porin OmpC | -1.91 | 5.91E-12 |
|  | 8912213 | *dppF* | dipeptide ABC transporter ATP-binding subunit DppF | -1.55 | 7.20E-12 |
|  | 8912405 | *EAMY_RS27710* | protein-tyrosine-phosphatase | 1.79 | 1.07E-11 |
|  | 8914283 | *EAMY_RS22290* | TraB/GumN family protein | 2.51 | 1.16E-11 |
|  | 8912926 | *EAMY_RS27665* | lipopolysaccharide biosynthesis protein | 1.57 | 1.16E-11 |
|  | 8914716 | *EAMY_RS33110* | aspartate aminotransferase family protein | 1.83 | 1.52E-11 |
|  | 8913687 | *ychH* | stress-induced protein YchH | -2.34 | 1.66E-11 |
|  | 8914346 | *cspD* | cold shock-like protein CspD | -2.76 | 1.66E-11 |
|  | 8912732 | *EAMY_RS20195* | peroxiredoxin | 1.87 | 2.24E-11 |
|  | 8914413 | *EAMY_RS24975* | porin OmpC | -1.72 | 5.32E-11 |
|  | 8913942 | *EAMY_RS20190* | thioredoxin family protein | 1.87 | 1.05E-10 |
|  | 8913479 | *EAMY_RS26275* | YciC family protein | 2.15 | 1.05E-10 |
|  | 8911738 | *EAMY_RS27055* | flippase | 2.22 | 1.05E-10 |
|  | 8914055 | *EAMY_RS34145* | OmpA family lipoprotein | 1.98 | 1.05E-10 |
|  | 30316917 | *EAMY_RS35010* | glycine zipper 2TM domain-containing protein | 2.87 | 1.61E-10 |
|  | 8911291 | *EAMY_RS19795* | sensor histidine kinase | 1.62 | 1.83E-10 |
|  | 8912994 | *EAMY_RS33745* | hypothetical protein | -1.59 | 1.83E-10 |
|  |  | *MSTRG.363.1* | unannotated transcript | 1.63 | 4.55E-10 |
|  | 8914651 | *EAMY_RS20185* | sigma-70 family RNA polymerase sigma factor | 1.97 | 6.61E-10 |
|  | 8913976 | *soxS* | superoxide response transcriptional regulator SoxS | 2.37 | 1.10E-09 |
|  | 8914376 | *efeO* | iron uptake system protein EfeO | -1.92 | 1.18E-09 |
|  | 8913785 | *acnA* | aconitate hydratase AcnA | 1.62 | 1.39E-09 |
|  | 8912847 | *fliD* | flagellar filament capping protein FliD | -2.10 | 1.39E-09 |
|  | 8914242 | *hpt* | hypoxanthine phosphoribosyltransferase | -2.71 | 2.23E-09 |
|  | 23673387 | *EAMY_RS28935* | DUF1471 domain-containing protein | 5.54 | 2.48E-09 |
|  | 8912408 | *EAMY_RS27695* | EpsG family protein | 2.02 | 2.86E-09 |
|  | 8914128 | *EAMY_RS22220* | hypothetical protein | -1.67 | 2.99E-09 |
|  | 8911433 | *EAMY_RS22225* | NUDIX domain-containing protein | -1.77 | 3.77E-09 |
|  | 8911620 | *EAMY_RS25605* | GNAT family N-acetyltransferase | 1.65 | 3.77E-09 |
|  | 8912711 | *EAMY_RS30930* | acyltransferase | 1.86 | 4.77E-09 |
|  | 8911735 | *flhC* | flagellar transcriptional regulator FlhC | -1.50 | 4.79E-09 |
|  | 8912879 | *fliT* | flagella biosynthesis regulatory protein FliT | -1.75 | 5.80E-09 |
|  | 8914660 | *EAMY_RS19455* | DUF1190 family protein | 2.42 | 8.62E-09 |
|  | 8914792 | *fpr* | ferredoxin--NADP(+) reductase | 2.66 | 1.00E-08 |
|  | 8914406 | *emtA* | membrane-bound lytic murein transglycosylase EmtA | 1.77 | 1.21E-08 |
|  | 8913299 | *exbB* | tol-pal system-associated acyl-CoA thioesterase | 2.85 | 1.87E-08 |
|  | 8913335 | *EAMY_RS23420* | HlyD family secretion protein | -1.63 | 2.48E-08 |
|  | 8912818 | *flgG* | flagellar basal-body rod protein FlgG | -2.15 | 2.77E-08 |
|  | 8912719 | *dppA* | dipeptide ABC transporter periplasmic-binding protein DppA | -1.76 | 2.80E-08 |
|  | 8913529 | *ppiA* | peptidylprolyl isomerase A | 1.74 | 3.00E-08 |
|  | 8912814 | *flgE* | flagellar hook protein FlgE | -2.32 | 4.96E-08 |
|  | 8912812 | *flgD* | flagellar hook assembly protein FlgD | -2.37 | 6.77E-08 |
|  | 8913318 | *EAMY_RS29135* | MFS transporter | 1.80 | 7.08E-08 |
|  | 8912816 | *EAMY_RS24205* | flagellar basal body rod protein FlgF | -2.15 | 9.78E-08 |
|  | 8913670 | *mscM* | miniconductance mechanosensitive channel MscM | 1.66 | 1.06E-07 |
|  | 8912407 | *EAMY_RS27700* | glycosyltransferase family 2 protein | 1.57 | 1.29E-07 |
|  | 8911749 | *EAMY_RS27145* | acyltransferase family protein | 2.43 | 1.82E-07 |
|  | 8912849 | *fliS* | flagellar export chaperone FliS | -1.70 | 1.84E-07 |
|  | 8911526 | *yccA* | FtsH protease modulator YccA | 1.75 | 2.35E-07 |
|  | 8911909 | *EAMY_RS29705* | winged helix-turn-helix domain-containing protein | -1.57 | 2.46E-07 |
|  | 8914442 | *EAMY_RS19660* | YlaC family protein | -1.62 | 2.80E-07 |
|  | 8912422 | *folA* | type 3 dihydrofolate reductase | 1.69 | 3.22E-07 |
|  | 8913471 | *EAMY_RS28135* | hypothetical protein | 1.69 | 3.64E-07 |
|  | 8911546 | *rcsA* | transcriptional regulator RcsA | 1.82 | 3.65E-07 |
|  | 69102765 | *EAMY_RS36510* | GIY-YIG nuclease family protein | -1.85 | 4.21E-07 |
|  | 8913157 | *ilvC* | ketol-acid reductoisomerase | -1.73 | 4.77E-07 |
|  | 8911658 | *asr* | acid resistance repetitive basic protein Asr | 2.22 | 5.37E-07 |
|  | 8911203 | *EAMY_RS17555* | reverse transcriptase family protein | -2.46 | 6.02E-07 |
|  | 8914416 | *EAMY_RS26280* | septation protein A | 1.64 | 6.79E-07 |
|  | 8914018 | *srlD* | sorbitol-6-phosphate dehydrogenase | 1.52 | 7.79E-07 |
|  | 8913940 | *dksA* | RNA polymerase-binding protein DksA | -1.54 | 8.65E-07 |
|  | 8912810 | *flgC* | flagellar basal body rod protein FlgC | -2.84 | 1.38E-06 |
|  | 8913269 | *EAMY_RS33720* | siderophore-interacting protein | 2.77 | 2.04E-06 |
|  | 8913092 | *EAMY_RS30310* | cystathionine gamma-synthase family protein | -1.74 | 4.64E-06 |
|  | 8911776 | *EAMY_RS27465* | Dabb family protein | -1.69 | 4.76E-06 |
|  | 8912746 | *EAMY_RS28240* | MFS transporter | 1.78 | 5.33E-06 |
|  | 8912892 | *panB* | 3-methyl-2-oxobutanoate hydroxymethyltransferase | -2.22 | 6.13E-06 |
|  | 8913439 | *proV* | glycine betaine/L-proline ABC transporter ATP-binding protein ProV | -1.78 | 7.84E-06 |
|  | 8914466 | *EAMY_RS25135* | heme ABC transporter ATP-binding protein | 1.78 | 8.35E-06 |
|  | 8912861 | *fliI* | flagellar protein export ATPase FliI | -1.82 | 1.27E-05 |
|  | 8912003 | *tssC* | type VI secretion system contractile sheath large subunit | -2.30 | 1.67E-05 |
|  | 8911543 | *EAMY_RS24350* | PrpF domain-containing protein | 1.66 | 2.64E-05 |
|  | 8912808 | *flgB* | flagellar basal body rod protein FlgB | -2.48 | 2.88E-05 |
|  | 8912438 | *EAMY_RS33115* | aminodeoxychorismate synthase component II | 1.66 | 3.05E-05 |
|  | 8914432 | *EAMY_RS25810* | DUF2946 domain-containing protein | -1.56 | 3.23E-05 |
|  | 8912768 | *EAMY_RS24005* | GlpM family protein | -3.13 | 3.40E-05 |
|  | 8911568 | *EAMY_RS24670* | flavocytochrome c | -1.76 | 4.12E-05 |
|  | 8911567 | *EAMY_RS24665* | anion permease | -2.00 | 6.64E-05 |
|  | 8913811 | *rsxC* | electron transport complex subunit RsxC | 2.03 | 7.61E-05 |
|  | 23673363 | *adhE* | pseudo | -1.66 | 1.56E-04 |
|  | 8913963 | *glnK* | P-II family nitrogen regulator | -1.92 | 1.63E-04 |
|  | 8912859 | *fliH* | flagellar assembly protein FliH | -2.11 | 1.87E-04 |
|  | 8911777 | *EAMY_RS27470* | DUF2000 domain-containing protein | -1.57 | 2.30E-04 |
|  | 8912522 | *tig* | trigger factor | 1.99 | 2.53E-04 |
|  | 8912857 | *fliG* | flagellar motor switch protein FliG | -1.58 | 3.23E-04 |
|  | 8913660 | *proW* | glycine betaine/L-proline ABC transporter permease ProW | -3.23 | 3.24E-04 |
|  | 8914827 | *nrdG* | anaerobic ribonucleoside-triphosphate reductase-activating protein | -1.58 | 3.49E-04 |
|  | 8912820 | *EAMY_RS24215* | flagellar basal body L-ring protein FlgH | -1.53 | 3.94E-04 |
|  | 8913386 | *EAMY_RS18960* | helix-turn-helix domain-containing protein | -1.82 | 4.39E-04 |
|  | 8912616 | *EAMY_RS19650* | spore coat protein U domain-containing protein | -2.41 | 5.33E-04 |
|  | 8911276 | *EAMY_RS19630* | spore coat U domain-containing protein | -1.95 | 9.18E-04 |
|  | 43837318 | *EAMY_RS36245* | hypothetical protein | -1.67 | 9.34E-04 |
|  | 8914295 | *ybbP* | putative ABC transporter permease subunit YbbP | 3.97 | 9.54E-04 |
|  | 69102775 | *EAMY_RS36560* | YnfU family zinc-binding protein | -1.97 | 1.04E-03 |
|  | 8913133 | *ppiD* | peptidylprolyl isomerase | 1.87 | 1.10E-03 |
|  | 8913570 | *EAMY_RS17820* | sulfate ABC transporter substrate-binding protein | -1.79 | 1.12E-03 |
|  | 8912618 | *EAMY_RS19640* | molecular chaperone | -1.92 | 1.16E-03 |
|  | 8912004 | *tssB* | type VI secretion system contractile sheath small subunit | -2.25 | 1.52E-03 |
|  | 8912016 | *EAMY_RS31370* | PepSY-associated TM helix domain-containing protein | -1.54 | 1.56E-03 |
|  | 8913616 | *argE* | acetylornithine deacetylase | 1.59 | 1.64E-03 |
|  | 8911554 | *EAMY_RS24585* | GNAT family N-acetyltransferase | -2.29 | 1.67E-03 |
|  | 8911839 | *EAMY_RS28575* | hypothetical protein | -1.58 | 3.97E-03 |
|  | 8913504 | *EAMY_RS19950* | Hrp pili protein HrpA | -1.59 | 4.66E-03 |
|  | 8913109 | *EAMY_RS19920* | sigma-70 family RNA polymerase sigma factor | -1.63 | 5.17E-03 |
|  | 8912703 | *gluQRS* | tRNA glutamyl-Q(34) synthetase GluQRS | -2.09 | 5.60E-03 |
|  | 8914435 | *EAMY_RS25980* | alpha-glucosidase | -1.62 | 5.94E-03 |
|  | 8914214 | *rfbB* | dTDP-glucose 4,6-dehydratase | 4.06 | 7.56E-03 |
|  | 8913535 | *panC* | pantoate--beta-alanine ligase | -1.55 | 8.11E-03 |
|  | 8913512 | *EAMY_RS28205* | hypothetical protein | -1.68 | 9.57E-03 |
|  | 8914123 | *trpA* | tryptophan synthase subunit alpha | -2.29 | 1.04E-02 |
|  | 8912577 | *tssG* | type VI secretion system baseplate subunit TssG | -1.95 | 1.06E-02 |
|  | 8911531 | *EAMY_RS24010* | DUF2594 family protein | -1.83 | 1.10E-02 |
|  | 8912007 | *tssM* | type VI secretion system membrane subunit TssM | -2.19 | 1.15E-02 |
|  | 8912504 | *nth* | endonuclease III | -1.74 | 1.33E-02 |
|  | 8913001 | *rpe* | ribulose-phosphate 3-epimerase | -4.62 | 1.36E-02 |
|  | 8912853 | *fliE* | flagellar hook-basal body complex protein FliE | -1.63 | 1.57E-02 |
|  | 8911990 | *tssF* | type VI secretion system baseplate subunit TssF | -1.75 | 1.71E-02 |
|  | 8911578 | *sirB1* | invasion regulator SirB1 | 2.58 | 1.81E-02 |
|  | 8913788 | *cfa* | cyclopropane fatty acyl phospholipid synthase | -5.13 | 1.88E-02 |
|  | 8914070 | *tauB* | taurine ABC transporter ATP-binding subunit | -1.63 | 2.08E-02 |
|  | 8912009 | *tssK* | type VI secretion system baseplate subunit TssK | -2.05 | 2.11E-02 |
|  | 8912005 | *tssA* | type VI secretion system protein TssA | -1.64 | 2.13E-02 |
|  | 23673432 | *EAMY_RS32640* | pyocin activator PrtN family protein | 2.95 | 2.21E-02 |
|  | 8912984 | *gltK* | glutamate/aspartate ABC transporter permease GltK | 1.57 | 2.43E-02 |
|  | 8914753 | *EAMY_RS34075* | LuxR C-terminal-related transcriptional regulator | 2.73 | 3.37E-02 |
|  | 69102808 | *kdpF* | K(+)-transporting ATPase subunit F | -1.92 | 3.67E-02 |
| **Copper Adaptation (SA1-S)** | 8912479 | *spy* | ATP-independent periplasmic protein-refolding chaperone Spy | 6.36 | 2.34E-21 |
|  | 8912415 | *wcaK* | colanic acid biosynthesis pyruvyl transferase WcaK | 3.80 | 2.34E-21 |
|  | 8912414 | *EAMY_RS27680* | phage tailspike protein | 4.07 | 2.34E-21 |
|  | 8913418 | *EAMY_RS24365* | glycine zipper 2TM domain-containing protein | 4.70 | 3.60E-21 |
|  | 8912357 | *EAMY_RS31520* | SulP family inorganic anion transporter | 4.14 | 8.84E-21 |
|  | 8914287 | *copA* | copper-exporting P-type ATPase CopA | 5.08 | 3.52E-20 |
|  | 8912406 | *EAMY_RS27705* | polysaccharide biosynthesis tyrosine autokinase | 3.30 | 3.52E-20 |
|  | 8912413 | *EAMY_RS27715* | polysaccharide export protein | 3.17 | 4.62E-20 |
|  | 8912412 | *wbaP* | undecaprenyl-phosphate galactose phosphotransferase WbaP | 3.56 | 2.43E-19 |
|  | 8912926 | *EAMY_RS27665* | lipopolysaccharide biosynthesis protein | 3.06 | 2.06E-18 |
|  | 8914463 | *ppsA* | phosphoenolpyruvate synthase | 3.44 | 1.65E-17 |
|  | 8914055 | *EAMY_RS34145* | OmpA family lipoprotein | 4.22 | 2.50E-17 |
|  | 8913587 | *efeB* | iron uptake transporter deferrochelatase/peroxidase subunit | -3.24 | 5.13E-17 |
|  | 8912405 | *EAMY_RS27710* | protein-tyrosine-phosphatase | 3.29 | 6.97E-17 |
|  | 8912410 | *EAMY_RS27685* | glycosyltransferase | 3.76 | 1.81E-16 |
|  | 8911620 | *EAMY_RS25605* | GNAT family N-acetyltransferase | 3.10 | 4.19E-16 |
|  | 8914378 | *efeU* | iron uptake transporter permease EfeU | -4.11 | 5.27E-16 |
|  | 8913162 | *EAMY_RS33810* | serralysin family metalloprotease | -2.84 | 5.27E-16 |
|  | 8914283 | *EAMY_RS22290* | TraB/GumN family protein | 3.38 | 1.10E-15 |
|  | 8913538 | *EAMY_RS30290* | ABC transporter permease | 1.72 | 1.10E-15 |
|  | 8911811 | *kup* | low affinity potassium transporter Kup | -3.48 | 1.19E-15 |
|  | 8911929 | *cueO* | multicopper oxidase CueO | 5.29 | 1.20E-15 |
|  | 8914174 | *ispC* | 1-deoxy-D-xylulose-5-phosphate reductoisomerase | 2.89 | 4.15E-15 |
|  | 8914129 | *sltY* | murein transglycosylase | 2.11 | 4.36E-15 |
|  | 30316917 | *EAMY_RS35010* | glycine zipper 2TM domain-containing protein | 3.90 | 5.35E-15 |
|  | 8912732 | *EAMY_RS20195* | peroxiredoxin | 2.70 | 1.13E-14 |
|  | 8914376 | *efeO* | iron uptake system protein EfeO | -3.51 | 2.01E-14 |
|  | 8912408 | *EAMY_RS27695* | EpsG family protein | 3.57 | 2.56E-14 |
|  | 8912409 | *EAMY_RS27690* | glycosyltransferase family 4 protein | 3.80 | 2.88E-14 |
|  | 8912404 | *ampC* | class C beta-lactamase | 2.47 | 2.94E-14 |
|  | 8914661 | *EAMY_RS19450* | glutathionylspermidine synthase family protein | 2.81 | 3.96E-14 |
|  | 8913942 | *EAMY_RS20190* | thioredoxin family protein | 2.71 | 3.96E-14 |
|  | 8914406 | *emtA* | membrane-bound lytic murein transglycosylase EmtA | 3.28 | 5.51E-14 |
|  | 8914077 | *ybbA* | putative ABC transporter ATP-binding protein YbbA | 2.79 | 6.76E-14 |
|  | 8911546 | *rcsA* | transcriptional regulator RcsA | 3.72 | 7.29E-14 |
|  | 8911267 | *EAMY_RS19515* | aminotransferase class III-fold pyridoxal phosphate-dependent enzyme | -2.43 | 9.97E-14 |
|  | 8911284 | *EAMY_RS19705* | disulfide bond formation protein B | 3.19 | 1.17E-13 |
|  | 8913630 | *EAMY_RS31515* | dipeptidase | 2.37 | 1.49E-13 |
|  | 8911849 | *EAMY_RS28685* | hypothetical protein | 3.23 | 2.02E-13 |
|  | 8912407 | *EAMY_RS27700* | glycosyltransferase family 2 protein | 3.32 | 2.45E-13 |
|  | 8913626 | *EAMY_RS19540* | type I polyketide synthase | -1.90 | 2.95E-13 |
|  | 8914346 | *cspD* | cold shock-like protein CspD | -3.79 | 2.95E-13 |
|  | 8912683 | *truA* | tRNA pseudouridine(38-40) synthase TruA | 1.90 | 3.72E-13 |
|  | 8913471 | *EAMY_RS28135* | hypothetical protein | 2.84 | 4.30E-13 |
|  | 8912198 | *EAMY_RS33740* | SMP-30/gluconolactonase/LRE family protein | 1.84 | 6.79E-13 |
|  | 8912510 | *EAMY_RS23225* | suppressor of fused domain protein | 2.74 | 8.39E-13 |
|  | 8913687 | *ychH* | stress-induced protein YchH | -2.52 | 8.80E-13 |
|  | 8914716 | *EAMY_RS33110* | aspartate aminotransferase family protein | 1.92 | 8.80E-13 |
|  | 8911738 | *EAMY_RS27055* | flippase | 2.56 | 8.92E-13 |
|  | 8912711 | *EAMY_RS30930* | acyltransferase | 2.99 | 1.06E-12 |
|  | 8911440 | *ybaK* | Cys-tRNA(Pro)/Cys-tRNA(Cys) deacylase YbaK | 2.03 | 1.07E-12 |
|  | 8914242 | *hpt* | hypoxanthine phosphoribosyltransferase | -3.08 | 1.13E-12 |
|  | 8914804 | *EAMY_RS32695* | YjbH domain-containing protein | 1.67 | 1.19E-12 |
|  | 8912924 | *galF* | UTP--glucose-1-phosphate uridylyltransferase GalF | 1.96 | 1.63E-12 |
|  | 8911735 | *flhC* | flagellar transcriptional regulator FlhC | -1.96 | 4.91E-12 |
|  | 8912994 | *EAMY_RS33745* | hypothetical protein | 2.10 | 5.27E-12 |
|  | 8913281 | *EAMY_RS19525* | beta-ketoacyl synthase N-terminal-like domain-containing protein | -2.16 | 5.33E-12 |
|  | 8914052 | *EAMY_RS19535* | type I polyketide synthase | -2.28 | 7.93E-12 |
|  | 8914013 | *EAMY_RS25670* | FAD-binding protein | -1.72 | 8.17E-12 |
|  | 8912695 | *EAMY_RS32250* | lysine N(6)-hydroxylase/L-ornithine N(5)-oxygenase family protein | -3.11 | 8.17E-12 |
|  | 8911268 | *EAMY_RS19545* | condensation domain-containing protein | -1.73 | 1.12E-11 |
|  | 8911612 | *EAMY_RS25565* | hypothetical protein | -2.49 | 3.04E-11 |
|  | 8914405 | *wbaP* | undecaprenyl-phosphate galactose phosphotransferase WbaP | 1.90 | 3.49E-11 |
|  | 8914651 | *EAMY_RS20185* | sigma-70 family RNA polymerase sigma factor | 2.09 | 3.80E-11 |
|  | 8914548 | *arnA* | bifunctional UDP-4-amino-4-deoxy-L-arabinose formyltransferase/UDP-glucuronic acid oxidase ArnA | 1.54 | 8.20E-11 |
|  | 8912693 | *EAMY_RS32255* | GNAT family N-acetyltransferase | -3.02 | 8.75E-11 |
|  | 8914507 | *EAMY_RS24385* | M15 family metallopeptidase | -1.95 | 1.24E-10 |
|  | 8912694 | *EAMY_RS32260* | TonB-dependent siderophore receptor | -1.52 | 2.17E-10 |
|  | 8912735 | *EAMY_RS20035* | LysR family transcriptional regulator | -2.64 | 2.36E-10 |
|  | 8914851 | *EAMY_RS23485* | serine hydrolase | 1.50 | 2.83E-10 |
|  | 8913280 | *EAMY_RS19520* | polyketide synthase | -2.07 | 3.20E-10 |
|  | 8913479 | *EAMY_RS26275* | YciC family protein | 1.91 | 4.61E-10 |
|  | 8911264 | *EAMY_RS19500* | creatininase family protein | -2.01 | 8.19E-10 |
|  | 23673343 | *arnC* | undecaprenyl-phosphate 4-deoxy-4-formamido-L-arabinose transferase | 1.62 | 8.62E-10 |
|  | 8911749 | *EAMY_RS27145* | acyltransferase family protein | 2.55 | 1.51E-09 |
|  | 8912604 | *EAMY_RS33105* | YccS/YhfK family putative transporter | 1.57 | 1.51E-09 |
|  | 69102764 | *EAMY_RS36505* | DUF5993 family protein | 3.35 | 1.51E-09 |
|  | 8914944 | *EAMY_RS28760* | YfeC-like transcriptional regulator | -2.73 | 2.37E-09 |
|  | 8911759 | *EAMY_RS27275* | metal ABC transporter substrate-binding protein | -1.67 | 2.38E-09 |
|  | 8911859 | *EAMY_RS28940* | histidine kinase | -1.63 | 3.41E-09 |
|  | 8911266 | *EAMY_RS19510* | nucleoside 2-deoxyribosyltransferase | -2.31 | 3.92E-09 |
|  | 8913786 | *ygiD* | 4,5-DOPA dioxygenase extradiol | 1.81 | 3.92E-09 |
|  | 8911543 | *EAMY_RS24350* | PrpF domain-containing protein | 2.61 | 3.92E-09 |
|  | 8914660 | *EAMY_RS19455* | DUF1190 family protein | 2.65 | 5.11E-09 |
|  | 8911707 | *htpX* | protease HtpX | 1.69 | 5.89E-09 |
|  | 8911706 | *EAMY_RS26685* | YobH family protein | 1.88 | 8.63E-09 |
|  | 8914442 | *EAMY_RS19660* | YlaC family protein | -2.00 | 1.34E-08 |
|  | 8913940 | *dksA* | RNA polymerase-binding protein DksA | -2.11 | 1.50E-08 |
|  | 23673438 | *EAMY_RS33805* | protease inhibitor Inh/omp19 family protein | -2.07 | 2.94E-08 |
|  | 8912636 | *cysW* | sulfate/thiosulfate ABC transporter permease CysW | 2.10 | 4.22E-08 |
|  | 8912639 | *cysN* | sulfate adenylyltransferase subunit CysN | 3.59 | 4.97E-08 |
|  | 8913529 | *ppiA* | peptidylprolyl isomerase A | 1.70 | 5.34E-08 |
|  | 8912107 | *EAMY_RS32245* | aspartate aminotransferase family protein | -3.08 | 5.87E-08 |
|  | 8911640 | *EAMY_RS25755* | ABC transporter substrate-binding protein | 1.69 | 6.04E-08 |
|  | 8912422 | *folA* | type 3 dihydrofolate reductase | 1.66 | 8.09E-08 |
|  | 30317028 | *EAMY_RS35565* | ogr/Delta-like zinc finger family protein | -2.26 | 8.85E-08 |
|  | 8912174 | *iolC* | 5-dehydro-2-deoxygluconokinase | 2.20 | 1.30E-07 |
|  | 8912630 | *EAMY_RS25640* | DUF4385 domain-containing protein | -1.58 | 1.37E-07 |
|  | 8911508 | *EAMY_RS23440* | sugar porter family MFS transporter | 2.92 | 1.40E-07 |
|  | 8914416 | *EAMY_RS26280* | septation protein A | 1.82 | 1.53E-07 |
|  | 8912554 | *motB* | flagellar motor protein MotB | -1.56 | 1.89E-07 |
|  | 8911281 | *EAMY_RS19685* | ABC transporter substrate-binding protein | -1.63 | 2.43E-07 |
|  | 8912648 | *cysC* | adenylyl-sulfate kinase | 2.88 | 2.83E-07 |
|  | 8913121 | *EAMY_RS19935* | sigma 54-interacting transcriptional regulator | 1.63 | 3.05E-07 |
|  | 8913299 | *exbB* | tol-pal system-associated acyl-CoA thioesterase | 2.36 | 3.13E-07 |
|  | 8913363 | *hrpB* | ATP-dependent helicase HrpB | -2.00 | 3.17E-07 |
|  | 8914861 | *EAMY_RS25825* | hypothetical protein | -1.88 | 4.69E-07 |
|  | 8912647 | *cysA* | sulfate/thiosulfate ABC transporter ATP-binding protein CysA | 1.87 | 6.29E-07 |
|  | 8912570 | *EAMY_RS19625* | SDR family oxidoreductase | -1.95 | 6.32E-07 |
|  | 8913976 | *soxS* | superoxide response transcriptional regulator SoxS | 1.76 | 7.10E-07 |
|  | 8911775 | *EAMY_RS27435* | YetF domain-containing protein | -1.54 | 7.54E-07 |
|  |  | *MSTRG.1974.1* | unannotated transcript | -1.66 | 1.24E-06 |
|  | 8913092 | *EAMY_RS30310* | cystathionine gamma-synthase family protein | -2.16 | 1.25E-06 |
|  | 8913184 | *iolD* | 3D-(3,5/4)-trihydroxycyclohexane-1,2-dione acylhydrolase (decyclizing) | 1.80 | 1.47E-06 |
|  | 8911644 | *EAMY_RS25785* | acyltransferase | -2.25 | 1.69E-06 |
|  | 8913439 | *proV* | glycine betaine/L-proline ABC transporter ATP-binding protein ProV | -2.11 | 2.07E-06 |
|  | 23673361 | *EAMY_RS25875* | DapH/DapD/GlmU-related protein | -1.91 | 2.11E-06 |
|  | 8911526 | *yccA* | FtsH protease modulator YccA | 1.50 | 2.28E-06 |
|  | 8911760 | *EAMY_RS27305* | protein kinase family protein | -1.58 | 2.63E-06 |
|  | 8913269 | *EAMY_RS33720* | siderophore-interacting protein | -4.83 | 4.11E-06 |
|  | 8913118 | *hrpN* | type III secretion system harpin HrpN | 1.58 | 4.66E-06 |
|  | 8913235 | *EAMY_RS19710* | phytochelatin synthase family protein | 2.02 | 6.33E-06 |
|  | 8913180 | *EAMY_RS33525* | CoA-acylating methylmalonate-semialdehyde dehydrogenase | 1.81 | 6.50E-06 |
|  | 8912641 | *cysD* | sulfate adenylyltransferase subunit CysD | 2.97 | 6.58E-06 |
|  | 8912438 | *EAMY_RS33115* | aminodeoxychorismate synthase component II | 1.99 | 1.05E-05 |
|  | 23673387 | *EAMY_RS28935* | DUF1471 domain-containing protein | 2.36 | 1.52E-05 |
|  | 8911358 | *cysG* | siroheme synthase CysG | 1.94 | 1.70E-05 |
|  | 8914091 | *EAMY_RS27825* | hypothetical protein | -2.03 | 2.22E-05 |
|  | 8914758 | *EAMY_RS17540* | response regulator transcription factor | -1.59 | 2.65E-05 |
|  | 8911692 | *tdk* | thymidine kinase | -1.60 | 4.87E-05 |
|  | 8911801 | *EAMY_RS28075* | restriction endonuclease | -1.94 | 2.32E-04 |
|  | 8913570 | *EAMY_RS17820* | sulfate ABC transporter substrate-binding protein | 2.15 | 2.49E-04 |
|  | 8914643 | *EAMY_RS20320* | UDP-3-O-(3-hydroxymyristoyl)glucosamine N-acyltransferase | -1.57 | 3.56E-04 |
|  | 8911203 | *EAMY_RS17555* | reverse transcriptase family protein | -1.65 | 3.60E-04 |
|  | 8913093 | *hrpT* | HrpT family type III secretion system protein | 2.16 | 4.92E-04 |
|  | 8914606 | *cas1e* | type I-E CRISPR-associated endonuclease Cas1e | -1.72 | 6.01E-04 |
|  | 8911998 | *EAMY_RS31245* | lysozyme inhibitor LprI family protein | -1.65 | 6.34E-04 |
|  | 8912522 | *tig* | trigger factor | 1.79 | 6.73E-04 |
|  | 8914295 | *ybbP* | putative ABC transporter permease subunit YbbP | 4.54 | 1.01E-03 |
|  | 23673399 | *can* | carbonate dehydratase | -1.80 | 1.37E-03 |
|  | 8914241 | *pcnB* | polynucleotide adenylyltransferase PcnB | -2.06 | 1.46E-03 |
|  | 8912923 | *EAMY_RS23970* | YccJ family protein | -1.66 | 1.92E-03 |
|  | 8913441 | *nrdI* | class Ib ribonucleoside-diphosphate reductase assembly flavoprotein NrdI | -1.66 | 2.32E-03 |
|  | 8913952 | *EAMY_RS27295* | tRNA-Asn | -1.94 | 2.62E-03 |
|  | 8912520 | *EAMY_RS20525* | Ail/Lom family outer membrane beta-barrel protein | -1.51 | 2.79E-03 |
|  | 8913908 | *EAMY_RS26095* | hypothetical protein | -1.55 | 3.46E-03 |
|  | 8913504 | *EAMY_RS19950* | Hrp pili protein HrpA | 2.01 | 3.50E-03 |
|  | 8913660 | *proW* | glycine betaine/L-proline ABC transporter permease ProW | -2.44 | 3.88E-03 |
|  | 8911531 | *EAMY_RS24010* | DUF2594 family protein | -1.87 | 4.12E-03 |
|  | 8914246 | *sfsA* | DNA/RNA nuclease SfsA | -2.80 | 5.45E-03 |
|  | 30316913 | *rprA* | ncRNA | 3.90 | 5.50E-03 |
|  | 8914161 | *EAMY_RS27745* | phosphatase PAP2 family protein | 1.67 | 6.92E-03 |
|  | 8912452 | *EAMY_RS21755* | YaiA family protein | -2.00 | 6.93E-03 |
|  | 8914625 | *rlmM* | 23S rRNA (cytidine(2498)-2'-O)-methyltransferase RlmM | 2.24 | 1.02E-02 |
|  | 8913104 | *thpR* | RNA 2',3'-cyclic phosphodiesterase | -1.81 | 1.28E-02 |
|  | 8912998 | *gntK* | gluconokinase | 1.86 | 1.47E-02 |
|  | 8913512 | *EAMY_RS28205* | hypothetical protein | -1.59 | 1.50E-02 |
|  | 8912689 | *deoA* | thymidine phosphorylase | 1.83 | 1.57E-02 |
|  | 8912154 | *tauD* | taurine dioxygenase | 2.01 | 1.64E-02 |
|  | 8911979 | *deoC* | deoxyribose-phosphate aldolase | 1.80 | 2.34E-02 |
|  | 8914069 | *tauA* | taurine ABC transporter substrate-binding protein | 3.10 | 2.42E-02 |
|  | 8914070 | *tauB* | taurine ABC transporter ATP-binding subunit | 1.93 | 2.76E-02 |
|  | 8913535 | *panC* | pantoate--beta-alanine ligase | -1.74 | 3.18E-02 |
|  | 8914054 | *tri1* | ADP-ribosylarginine hydrolase Tri1 | 1.91 | 3.34E-02 |
|  | 23673433 | *yjbE* | exopolysaccharide production protein YjbE | 2.02 | 3.34E-02 |
|  | 8912063 | *EAMY_RS31885* | tRNA-Gly | -1.56 | 3.61E-02 |
|  | 8912873 | *fliO* | flagellar biosynthetic protein FliO | 2.00 | 4.08E-02 |
|  | 8913509 | *prfC* | peptide chain release factor 3 | 1.68 | 4.18E-02 |
|  | 8913990 | *sctT* | type III secretion system export apparatus subunit SctT | -2.76 | 4.65E-02 |
|  | 8912868 | *fliL* | flagellar basal body-associated protein FliL | -1.71 | 4.95E-02 |
| **Copper Adaptation (SA3-S)** | 8912415 | *wcaK* | colanic acid biosynthesis pyruvyl transferase WcaK | 4.32 | 2.18E-22 |
|  | 8912479 | *spy* | ATP-independent periplasmic protein-refolding chaperone Spy | 6.97 | 3.35E-22 |
|  | 8912414 | *EAMY_RS27680* | phage tailspike protein | 4.31 | 3.35E-22 |
|  | 8912406 | *EAMY_RS27705* | polysaccharide biosynthesis tyrosine autokinase | 4.12 | 3.35E-22 |
|  | 8912413 | *EAMY_RS27715* | polysaccharide export protein | 3.89 | 3.35E-22 |
|  | 8911759 | *EAMY_RS27275* | metal ABC transporter substrate-binding protein | -5.29 | 1.84E-21 |
|  | 8913950 | *sitC* | iron/manganese ABC transporter permease subunit SitC | -6.04 | 8.52E-21 |
|  | 8912732 | *EAMY_RS20195* | peroxiredoxin | 4.74 | 1.31E-20 |
|  | 8914463 | *ppsA* | phosphoenolpyruvate synthase | 4.53 | 2.32E-20 |
|  | 8913942 | *EAMY_RS20190* | thioredoxin family protein | 4.75 | 3.64E-20 |
|  | 8912926 | *EAMY_RS27665* | lipopolysaccharide biosynthesis protein | 3.55 | 3.64E-20 |
|  | 8914055 | *EAMY_RS34145* | OmpA family lipoprotein | 5.44 | 3.64E-20 |
|  | 8912412 | *wbaP* | undecaprenyl-phosphate galactose phosphotransferase WbaP | 3.79 | 3.79E-20 |
|  | 8913418 | *EAMY_RS24365* | glycine zipper 2TM domain-containing protein | 4.13 | 4.07E-20 |
|  | 8911454 | *EAMY_RS22430* | OsmC family protein | -3.32 | 4.77E-20 |
|  | 8912357 | *EAMY_RS31520* | SulP family inorganic anion transporter | 3.74 | 6.24E-20 |
|  | 8914287 | *copA* | copper-exporting P-type ATPase CopA | 4.68 | 1.75E-19 |
|  | 8912405 | *EAMY_RS27710* | protein-tyrosine-phosphatase | 4.03 | 2.51E-19 |
|  | 8913389 | *mraY* | phospho-N-acetylmuramoyl-pentapeptide-transferase | 2.16 | 7.80E-19 |
|  | 8912410 | *EAMY_RS27685* | glycosyltransferase | 4.45 | 1.07E-18 |
|  | 8912962 | *EAMY_RS23395* | DksA/TraR family C4-type zinc finger protein | -2.92 | 1.77E-18 |
|  | 8911411 | *EAMY_RS21710* | biofilm development regulator YmgB/AriR family protein | -2.38 | 5.57E-18 |
|  | 8913392 | *ftsW* | cell division protein FtsW | 2.05 | 1.31E-17 |
|  | 8914651 | *EAMY_RS20185* | sigma-70 family RNA polymerase sigma factor | 3.76 | 1.62E-17 |
|  | 8911284 | *EAMY_RS19705* | disulfide bond formation protein B | 4.32 | 2.01E-17 |
|  | 8913391 | *murE* | UDP-N-acetylmuramoyl-L-alanyl-D-glutamate--2,6-diaminopimelate ligase | 2.05 | 2.15E-17 |
|  | 8914413 | *EAMY_RS24975* | porin OmpC | -3.55 | 2.34E-17 |
|  | 8911807 | *ompC* | porin OmpC | 4.93 | 2.75E-17 |
|  | 8914129 | *sltY* | murein transglycosylase | 2.59 | 2.78E-17 |
|  | 8911811 | *kup* | low affinity potassium transporter Kup | -3.48 | 3.59E-17 |
|  | 8912409 | *EAMY_RS27690* | glycosyltransferase family 4 protein | 4.57 | 1.14E-16 |
|  | 8914378 | *efeU* | iron uptake transporter permease EfeU | -3.38 | 1.78E-16 |
|  | 8914716 | *EAMY_RS33110* | aspartate aminotransferase family protein | 2.73 | 2.28E-16 |
|  | 8913388 | *murG* | undecaprenyldiphospho-muramoylpentapeptide beta-N-acetylglucosaminyltransferase | 1.94 | 2.51E-16 |
|  | 8914269 | *EAMY_RS21760* | IclR family transcriptional regulator | -2.63 | 3.49E-16 |
|  | 8913587 | *efeB* | iron uptake transporter deferrochelatase/peroxidase subunit | -2.47 | 3.61E-16 |
|  | 8914242 | *hpt* | hypoxanthine phosphoribosyltransferase | -4.71 | 3.92E-16 |
|  | 8911735 | *flhC* | flagellar transcriptional regulator FlhC | -2.81 | 5.39E-16 |
|  | 8911738 | *EAMY_RS27055* | flippase | 3.33 | 5.39E-16 |
|  | 8914661 | *EAMY_RS19450* | glutathionylspermidine synthase family protein | 3.30 | 5.45E-16 |
|  | 8911929 | *cueO* | multicopper oxidase CueO | 5.39 | 5.45E-16 |
|  | 8911942 | *EAMY_RS30490* | omptin family outer membrane protease | -2.30 | 5.45E-16 |
|  | 8912408 | *EAMY_RS27695* | EpsG family protein | 4.02 | 5.53E-16 |
|  | 8914851 | *EAMY_RS23485* | serine hydrolase | 2.53 | 6.94E-16 |
|  | 8913538 | *EAMY_RS30290* | ABC transporter permease | 1.70 | 9.93E-16 |
|  | 8912407 | *EAMY_RS27700* | glycosyltransferase family 2 protein | 4.03 | 1.03E-15 |
|  | 8914804 | *EAMY_RS32695* | YjbH domain-containing protein | 2.20 | 1.03E-15 |
|  | 8911855 | *EAMY_RS28735* | Nramp family divalent metal transporter | -2.68 | 1.13E-15 |
|  | 8914111 | *EAMY_RS26430* | UDP-glucose/GDP-mannose dehydrogenase family protein | 2.22 | 1.58E-15 |
|  | 8911877 | *denD* | D-erythronate dehydrogenase | 2.63 | 2.08E-15 |
|  | 8912711 | *EAMY_RS30930* | acyltransferase | 3.78 | 2.18E-15 |
|  | 8911849 | *EAMY_RS28685* | hypothetical protein | 3.72 | 3.04E-15 |
|  | 8914035 | *sucC* | ADP-forming succinate--CoA ligase subunit beta | 1.99 | 3.04E-15 |
|  | 8914406 | *emtA* | membrane-bound lytic murein transglycosylase EmtA | 3.59 | 3.87E-15 |
|  | 8913507 | *EAMY_RS22435* | RcnB family protein | -3.04 | 4.42E-15 |
|  | 8912568 | *EAMY_RS31450* | KpsF/GutQ family sugar-phosphate isomerase | 1.74 | 4.54E-15 |
|  | 8913687 | *ychH* | stress-induced protein YchH | -2.99 | 5.07E-15 |
|  | 8912753 | *emrA* | multidrug efflux MFS transporter periplasmic adaptor subunit EmrA | 2.52 | 6.76E-15 |
|  | 8912198 | *EAMY_RS33740* | SMP-30/gluconolactonase/LRE family protein | 2.13 | 7.72E-15 |
|  | 8914507 | *EAMY_RS24385* | M15 family metallopeptidase | -2.78 | 1.00E-14 |
|  | 8911821 | *pta* | phosphate acetyltransferase | 2.08 | 1.42E-14 |
|  | 41697605 | *EAMY_RS35880* | pseudo | -2.46 | 1.42E-14 |
|  | 8914346 | *cspD* | cold shock-like protein CspD | -3.56 | 1.56E-14 |
|  | 8911532 | *EAMY_RS24050* | MarR family transcriptional regulator | -1.95 | 1.68E-14 |
|  | 8913162 | *EAMY_RS33810* | serralysin family metalloprotease | -2.33 | 1.94E-14 |
|  | 8913315 | *EAMY_RS23920* | DUF2057 family protein | -2.33 | 2.20E-14 |
|  | 8913909 | *EAMY_RS27540* | APC family permease | 2.62 | 2.77E-14 |
|  | 8912908 | *ftsL* | cell division protein FtsL | 1.60 | 2.81E-14 |
|  | 8914174 | *ispC* | 1-deoxy-D-xylulose-5-phosphate reductoisomerase | 2.57 | 2.96E-14 |
|  | 8914163 | *galU* | UTP--glucose-1-phosphate uridylyltransferase GalU | 1.82 | 3.21E-14 |
|  | 41697647 | *EAMY_RS36095* | hypothetical protein | 1.98 | 3.63E-14 |
|  | 8911605 | *guaD* | guanine deaminase | 1.91 | 5.08E-14 |
|  | 8914283 | *EAMY_RS22290* | TraB/GumN family protein | 2.78 | 5.56E-14 |
|  | 69102764 | *EAMY_RS36505* | DUF5993 family protein | 4.83 | 5.59E-14 |
|  | 8914226 | *EAMY_RS29215* | M4 family metallopeptidase | -2.52 | 6.16E-14 |
|  | 8913012 | *EAMY_RS23510* | YbjC family protein | 1.81 | 6.20E-14 |
|  | 8911850 | *EAMY_RS28690* | hypothetical protein | -1.89 | 6.65E-14 |
|  | 8912211 | *EAMY_RS33860* | AsmA family protein | -1.77 | 8.00E-14 |
|  | 8911546 | *rcsA* | transcriptional regulator RcsA | 3.57 | 8.52E-14 |
|  | 8914013 | *EAMY_RS25670* | FAD-binding protein | -2.04 | 9.16E-14 |
|  | 8913318 | *EAMY_RS29135* | MFS transporter | 3.33 | 1.33E-13 |
|  | 8914339 | *dps* | DNA starvation/stationary phase protection protein Dps | -2.06 | 1.41E-13 |
|  | 8911765 | *EAMY_RS27355* | glycosyltransferase family 8 protein | -1.51 | 2.05E-13 |
|  | 8914929 | *mlaC* | phospholipid-binding protein MlaC | 2.16 | 2.18E-13 |
|  | 8911485 | *EAMY_RS23200* | Yip1 family protein | -3.18 | 2.33E-13 |
|  | 8914930 | *mlaD* | outer membrane lipid asymmetry maintenance protein MlaD | 1.91 | 2.57E-13 |
|  | 8913479 | *EAMY_RS26275* | YciC family protein | 2.61 | 2.65E-13 |
|  | 8911620 | *EAMY_RS25605* | GNAT family N-acetyltransferase | 2.26 | 2.69E-13 |
|  | 8912749 | *emrB* | multidrug efflux MFS transporter permease subunit EmrB | 2.87 | 2.69E-13 |
|  | 8913135 | *hutI* | imidazolonepropionase | -2.80 | 2.73E-13 |
|  | 8912604 | *EAMY_RS33105* | YccS/YhfK family putative transporter | 2.14 | 2.73E-13 |
|  | 8914034 | *odhB* | 2-oxoglutarate dehydrogenase complex dihydrolipoyllysine-residue succinyltransferase | 1.86 | 3.06E-13 |
|  | 8914077 | *ybbA* | putative ABC transporter ATP-binding protein YbbA | 2.53 | 4.12E-13 |
|  | 8911758 | *mtfA* | DgsA anti-repressor MtfA | -1.80 | 4.28E-13 |
|  | 8914250 | *degP* | serine endoprotease DegP | 2.51 | 4.75E-13 |
|  | 8913021 | *tgt* | tRNA guanosine(34) transglycosylase Tgt | 1.52 | 5.09E-13 |
|  | 8913256 | *skp* | molecular chaperone Skp | 1.94 | 5.30E-13 |
|  | 8914581 | *murQ* | N-acetylmuramic acid 6-phosphate etherase | 1.96 | 5.46E-13 |
|  | 8914376 | *efeO* | iron uptake system protein EfeO | -2.55 | 5.51E-13 |
|  | 30316917 | *EAMY_RS35010* | glycine zipper 2TM domain-containing protein | 3.08 | 5.72E-13 |
|  | 8913266 | *EAMY_RS32730* | Na/Pi cotransporter family protein | -1.96 | 6.02E-13 |
|  | 8911612 | *EAMY_RS25565* | hypothetical protein | -2.54 | 8.75E-13 |
|  | 8912719 | *dppA* | dipeptide ABC transporter periplasmic-binding protein DppA | -2.78 | 8.75E-13 |
|  | 8912913 | *EAMY_RS30680* | D-alanine--D-alanine ligase | 1.68 | 9.48E-13 |
|  | 8913934 | *pgm* | phosphoglucomutase (alpha-D-glucose-1,6-bisphosphate-dependent) | 1.94 | 9.85E-13 |
|  | 8914275 | *secD* | protein translocase subunit SecD | 1.99 | 1.11E-12 |
|  | 8912059 | *hflC* | protease modulator HflC | 1.77 | 1.14E-12 |
|  | 8914075 | *hns* | histone-like nucleoid-structuring protein H-NS | -1.82 | 1.47E-12 |
|  | 8913134 | *EAMY_RS23320* | HutD family protein | -3.40 | 1.63E-12 |
|  | 8914640 | *dcp* | peptidyl-dipeptidase Dcp | 1.88 | 1.63E-12 |
|  | 8912683 | *truA* | tRNA pseudouridine(38-40) synthase TruA | 1.73 | 1.83E-12 |
|  | 8913151 | *ilvD* | dihydroxy-acid dehydratase | 1.67 | 1.90E-12 |
|  | 8912465 | *gntU* | gluconate transporter | 1.73 | 2.43E-12 |
|  | 8913310 | *EAMY_RS24375* | hypothetical protein | -1.50 | 2.54E-12 |
|  | 8913129 | *prc* | carboxy terminal-processing peptidase | 1.56 | 2.61E-12 |
|  | 8912721 | *dppB* | dipeptide ABC transporter permease DppB | -1.72 | 3.74E-12 |
|  | 8914320 | *seqA* | replication initiation negative regulator SeqA | 1.62 | 4.96E-12 |
|  | 8912630 | *EAMY_RS25640* | DUF4385 domain-containing protein | -2.40 | 7.50E-12 |
|  | 8913046 | *hflK* | FtsH protease activity modulator HflK | 1.93 | 7.90E-12 |
|  | 8912542 | *EAMY_RS27870* | NAD-dependent malic enzyme | 1.89 | 9.83E-12 |
|  | 8911783 | *EAMY_RS27520* | DUF496 family protein | 1.81 | 1.01E-11 |
|  | 8911608 | *EAMY_RS25545* | DUF441 domain-containing protein | -1.63 | 1.21E-11 |
|  | 8911707 | *htpX* | protease HtpX | 2.22 | 1.21E-11 |
|  | 8913671 | *lpxO* | lipid A hydroxylase LpxO | -2.30 | 1.37E-11 |
|  | 8911550 | *EAMY_RS24485* | hypothetical protein | -1.57 | 1.37E-11 |
|  |  | *MSTRG.363.1* | unannotated transcript | 1.82 | 1.37E-11 |
|  | 8913400 | *EAMY_RS23235* | glycosyltransferase | -1.60 | 1.63E-11 |
|  | 8914689 | *yhcN* | peroxide/acid stress response protein YhcN | 4.20 | 1.83E-11 |
|  | 8913439 | *proV* | glycine betaine/L-proline ABC transporter ATP-binding protein ProV | -4.49 | 1.87E-11 |
|  | 8914714 | *crp* | cAMP-activated global transcriptional regulator CRP | 1.60 | 2.30E-11 |
|  | 8913927 | *secF* | protein translocase subunit SecF | 1.90 | 2.35E-11 |
|  | 8914891 | *amiA* | N-acetylmuramoyl-L-alanine amidase AmiA | 1.58 | 2.40E-11 |
|  | 8914943 | *ATP* | phosphoenolpyruvate carboxykinase | 1.64 | 2.71E-11 |
|  | 8914018 | *srlD* | sorbitol-6-phosphate dehydrogenase | 3.03 | 2.72E-11 |
|  | 8912554 | *motB* | flagellar motor protein MotB | -2.23 | 2.91E-11 |
|  | 8912365 | *fabF* | beta-ketoacyl-ACP synthase II | 2.00 | 3.13E-11 |
|  | 8912990 | *EAMY_RS34040* | NAD(P)-dependent alcohol dehydrogenase | 1.90 | 3.13E-11 |
|  | 8911440 | *ybaK* | Cys-tRNA(Pro)/Cys-tRNA(Cys) deacylase YbaK | 1.67 | 3.64E-11 |
|  | 8912601 | *EAMY_RS21630* | hypothetical protein | -2.40 | 3.85E-11 |
|  | 8914540 | *EAMY_RS28020* | YejG family protein | -2.17 | 3.96E-11 |
|  | 8913053 | *hutU* | urocanate hydratase | -2.72 | 4.05E-11 |
|  | 8914874 | *EAMY_RS25055* | fructosamine kinase family protein | -1.90 | 4.45E-11 |
|  | 8914405 | *wbaP* | undecaprenyl-phosphate galactose phosphotransferase WbaP | 1.81 | 4.45E-11 |
|  | 8912523 | *proY* | proline-specific permease ProY | 1.57 | 5.58E-11 |
|  | 8912282 | *tuf* | elongation factor Tu | 1.65 | 5.86E-11 |
|  | 8914708 | *fkpA* | FKBP-type peptidyl-prolyl cis-trans isomerase | 1.93 | 6.14E-11 |
|  | 8911760 | *EAMY_RS27305* | protein kinase family protein | -2.60 | 7.27E-11 |
|  | 8913922 | *sucA* | 2-oxoglutarate dehydrogenase E1 component | 1.61 | 7.39E-11 |
|  | 8914408 | *kdsA* | 3-deoxy-8-phosphooctulonate synthase | 1.81 | 7.39E-11 |
|  | 8911466 | *EAMY_RS22550* | hypothetical protein | -2.01 | 8.03E-11 |
|  | 8913529 | *ppiA* | peptidylprolyl isomerase A | 2.25 | 8.37E-11 |
|  | 8912482 | *atpD* | F0F1 ATP synthase subunit beta | 1.74 | 8.58E-11 |
|  | 8914015 | *EAMY_RS31475* | PTS glucitol/sorbitol transporter subunit IIB | 2.67 | 1.02E-10 |
|  | 8914347 | *EAMY_RS26745* | 2OG-Fe dioxygenase family protein | -2.39 | 1.05E-10 |
|  | 8912765 | *ppnN* | nucleotide 5'-monophosphate nucleosidase PpnN | 1.96 | 1.06E-10 |
|  | 8912735 | *EAMY_RS20035* | LysR family transcriptional regulator | -2.17 | 1.22E-10 |
|  | 8913943 | *EAMY_RS21120* | hypothetical protein | 1.58 | 1.48E-10 |
|  | 8913630 | *EAMY_RS31515* | dipeptidase | 1.66 | 1.49E-10 |
|  | 8914058 | *EAMY_RS25635* | carbonic anhydrase | -1.71 | 1.59E-10 |
|  | 8914660 | *EAMY_RS19455* | DUF1190 family protein | 3.01 | 1.61E-10 |
|  | 8914591 | *trxC* | thioredoxin TrxC | -2.04 | 1.62E-10 |
|  | 8911291 | *EAMY_RS19795* | sensor histidine kinase | 1.51 | 1.81E-10 |
|  | 8912245 | *EAMY_RS34340* | F0F1 ATP synthase subunit epsilon | 1.87 | 1.86E-10 |
|  | 8914016 | *EAMY_RS31470* | glucitol/sorbitol-specific PTS transporter subunit IIA | 2.77 | 1.96E-10 |
|  | 8912164 | *glpD* | glycerol-3-phosphate dehydrogenase | -1.65 | 2.03E-10 |
|  | 8911193 | *EAMY_RS17320* | hypothetical protein | 1.77 | 2.10E-10 |
|  | 8913361 | *murF* | UDP-N-acetylmuramoyl-tripeptide--D-alanyl-D-alanine ligase | 1.96 | 2.36E-10 |
|  | 8911706 | *EAMY_RS26685* | YobH family protein | 2.07 | 2.36E-10 |
|  | 8911808 | *rcsD* | phosphotransferase RcsD | 1.63 | 2.36E-10 |
|  | 8914743 | *EAMY_RS33965* | organic hydroperoxide resistance protein | 1.50 | 2.36E-10 |
|  | 8914416 | *EAMY_RS26280* | septation protein A | 2.28 | 2.48E-10 |
|  | 30316876 | *EAMY_RS34805* | hypothetical protein | -2.70 | 2.56E-10 |
|  | 8914504 | *EAMY_RS27250* | hypothetical protein | -1.74 | 2.67E-10 |
|  | 30316924 | *EAMY_RS35045* | YmiA family putative membrane protein | -2.24 | 2.72E-10 |
|  | 8912705 | *udp* | uridine phosphorylase | 1.54 | 3.40E-10 |
|  | 8913311 | *mglB* | galactose/glucose ABC transporter substrate-binding protein MglB | 1.71 | 3.45E-10 |
|  | 8912909 | *ftsH* | ATP-dependent zinc metalloprotease FtsH | 2.18 | 3.58E-10 |
|  | 8911658 | *asr* | acid resistance repetitive basic protein Asr | 2.52 | 3.58E-10 |
|  | 8914121 | *rplS* | 50S ribosomal protein L19 | 2.59 | 4.23E-10 |
|  | 8912755 | *bamB* | outer membrane protein assembly factor BamB | 1.52 | 4.27E-10 |
|  | 8913092 | *EAMY_RS30310* | cystathionine gamma-synthase family protein | -3.05 | 4.57E-10 |
|  | 8913824 | *rplW* | 50S ribosomal protein L23 | 2.08 | 4.66E-10 |
|  | 23673368 | *EAMY_RS27210* | DUF6388 family protein | -1.90 | 5.14E-10 |
|  | 23673361 | *EAMY_RS25875* | DapH/DapD/GlmU-related protein | -2.73 | 5.21E-10 |
|  | 8911401 | *EAMY_RS21635* | hypothetical protein | -2.62 | 5.40E-10 |
|  | 8912932 | *rssB* | two-component system response regulator RssB | -1.79 | 5.42E-10 |
|  | 8914008 | *EAMY_RS20230* | sugar porter family MFS transporter | 1.51 | 6.28E-10 |
|  | 8911886 | *EAMY_RS29225* | membrane-bound PQQ-dependent dehydrogenase, glucose/quinate/shikimate family | -1.69 | 6.48E-10 |
|  | 8911830 | *EAMY_RS28455* | DedA family protein | 1.83 | 7.93E-10 |
|  | 8913632 | *pqqC* | pyrroloquinoline-quinone synthase PqqC | 2.66 | 7.93E-10 |
|  | 8913525 | *gspS* | type II secretion system pilot lipoprotein GspS | -1.58 | 8.17E-10 |
|  | 8912521 | *EAMY_RS31930* | entericidin A/B family lipoprotein | -1.83 | 8.63E-10 |
|  | 8914792 | *fpr* | ferredoxin--NADP(+) reductase | 2.89 | 8.92E-10 |
|  | 8912110 | *EAMY_RS32270* | glucose 1-dehydrogenase | -1.65 | 9.29E-10 |
|  | 8911851 | *EAMY_RS28695* | universal stress protein | -1.97 | 1.24E-09 |
|  | 8914629 | *EAMY_RS24695* | YgdI/YgdR family lipoprotein | -2.37 | 1.37E-09 |
|  | 8914146 | *EAMY_RS25480* | DUF1460 domain-containing protein | 1.94 | 1.40E-09 |
|  | 8912570 | *EAMY_RS19625* | SDR family oxidoreductase | -2.49 | 1.50E-09 |
|  | 8913299 | *exbB* | tol-pal system-associated acyl-CoA thioesterase | 2.98 | 1.88E-09 |
|  | 77383444 | *EAMY_RS36770* | hypothetical protein | -2.33 | 1.89E-09 |
|  | 8914598 | *trmD* | tRNA (guanosine(37)-N1)-methyltransferase TrmD | 1.96 | 1.97E-09 |
|  | 8911322 | *tkt* | transketolase | 1.64 | 2.04E-09 |
|  | 30317028 | *EAMY_RS35565* | ogr/Delta-like zinc finger family protein | -2.20 | 2.56E-09 |
|  | 8914945 | *rplI* | 50S ribosomal protein L9 | 1.54 | 2.63E-09 |
|  | 8912391 | *budA* | acetolactate decarboxylase | 2.27 | 2.75E-09 |
|  | 8911262 | *ribB* | 3,4-dihydroxy-2-butanone-4-phosphate synthase | -3.13 | 2.78E-09 |
|  | 8912438 | *EAMY_RS33115* | aminodeoxychorismate synthase component II | 2.86 | 2.84E-09 |
|  | 8914909 | *parE* | DNA topoisomerase IV subunit B | 2.03 | 2.97E-09 |
|  | 8911328 | *EAMY_RS20430* | hemolysin III family protein | -1.93 | 3.06E-09 |
|  | 8914619 | *cas7e* | type I-E CRISPR-associated protein Cas7/Cse4/CasC | 1.58 | 3.14E-09 |
|  | 8911595 | *EAMY_RS25320* | DUF1289 domain-containing protein | -1.92 | 3.36E-09 |
|  | 8911614 | *EAMY_RS25570* | TonB-dependent receptor | -1.60 | 3.40E-09 |
|  | 8914410 | *EAMY_RS24840* | SulP family inorganic anion transporter | -1.62 | 3.51E-09 |
|  | 8911564 | *EAMY_RS24655* | hypothetical protein | -1.69 | 3.54E-09 |
|  | 23673438 | *EAMY_RS33805* | protease inhibitor Inh/omp19 family protein | -1.84 | 3.54E-09 |
|  | 8914571 | *dapE* | succinyl-diaminopimelate desuccinylase | 1.97 | 4.17E-09 |
|  | 8912483 | *atpG* | F0F1 ATP synthase subunit gamma | 1.69 | 4.26E-09 |
|  | 8914442 | *EAMY_RS19660* | YlaC family protein | -1.75 | 4.35E-09 |
|  |  | *MSTRG.2581.1* | unannotated transcript | 1.51 | 4.36E-09 |
|  | 8912480 | *atpH* | F0F1 ATP synthase subunit delta | 1.75 | 4.71E-09 |
|  | 8913826 | *rplC* | 50S ribosomal protein L3 | 2.08 | 5.54E-09 |
|  | 8911885 | *EAMY_RS29190* | pseudo | -3.23 | 5.61E-09 |
|  | 8911281 | *EAMY_RS19685* | ABC transporter substrate-binding protein | -1.72 | 5.74E-09 |
|  | 8911824 | *EAMY_RS28370* | 4-aminobutyrate--2-oxoglutarate transaminase | 2.08 | 5.74E-09 |
|  | 8911558 | *EAMY_RS24600* | hypothetical protein | -2.71 | 6.15E-09 |
|  | 8914109 | *EAMY_RS26295* | YciI family protein | -1.70 | 6.19E-09 |
|  | 8913836 | *rpsC* | 30S ribosomal protein S3 | 1.50 | 6.35E-09 |
|  | 8914758 | *EAMY_RS17540* | response regulator transcription factor | -2.33 | 6.84E-09 |
|  | 8914617 | *EAMY_RS20880* | amino acid ABC transporter substrate-binding protein | 1.69 | 7.47E-09 |
|  | 23673348 | *yccX* | acylphosphatase | -1.83 | 7.47E-09 |
|  | 8913040 | *EAMY_RS24890* | SirB2 family protein | 1.61 | 7.56E-09 |
|  | 8913363 | *hrpB* | ATP-dependent helicase HrpB | -2.11 | 7.75E-09 |
|  | 8911587 | *EAMY_RS25050* | YniB family protein | -1.65 | 8.25E-09 |
|  | 8911909 | *EAMY_RS29705* | winged helix-turn-helix domain-containing protein | -1.82 | 9.44E-09 |
|  | 77383446 | *EAMY_RS36780* | hypothetical protein | -1.98 | 9.71E-09 |
|  | 8911321 | *EAMY_RS20285* | M48 family metallopeptidase | 1.71 | 1.09E-08 |
|  | 8911964 | *dnaJ* | molecular chaperone DnaJ | 2.37 | 1.10E-08 |
|  | 8912422 | *folA* | type 3 dihydrofolate reductase | 1.72 | 1.19E-08 |
|  | 8913670 | *mscM* | miniconductance mechanosensitive channel MscM | 1.60 | 1.20E-08 |
|  | 8911625 | *EAMY_RS25630* | methyl-accepting chemotaxis protein | -1.86 | 1.21E-08 |
|  | 8911692 | *tdk* | thymidine kinase | -2.40 | 1.25E-08 |
|  | 8913842 | *rpsS* | 30S ribosomal protein S19 | 1.59 | 1.29E-08 |
|  | 8913139 | *hutH* | histidine ammonia-lyase | -2.22 | 1.36E-08 |
|  | 23673369 | *EAMY_RS27215* | DUF2158 domain-containing protein | -1.88 | 1.36E-08 |
|  | 8911684 | *EAMY_RS26265* | BON domain-containing protein | -1.68 | 1.50E-08 |
|  | 8912195 | *EAMY_RS33705* | hypothetical protein | -1.55 | 1.63E-08 |
|  | 8913163 | *EAMY_RS22475* | hypothetical protein | -2.57 | 1.64E-08 |
|  | 8912999 | *EAMY_RS28375* | ABC transporter substrate-binding protein | 1.68 | 1.66E-08 |
|  | 8914869 | *EAMY_RS25515* | DUF1283 family protein | 1.81 | 1.74E-08 |
|  | 8913976 | *soxS* | superoxide response transcriptional regulator SoxS | 2.02 | 1.83E-08 |
|  | 8912896 | *EAMY_RS24675* | FAD:protein FMN transferase | -1.91 | 1.95E-08 |
|  | 8912208 | *EAMY_RS33840* | DUF1158 family protein | -1.68 | 2.03E-08 |
|  | 8912695 | *EAMY_RS32250* | lysine N(6)-hydroxylase/L-ornithine N(5)-oxygenase family protein | -1.75 | 2.21E-08 |
|  | 8912525 | *EAMY_RS25120* | NlpC/P60 family protein | -1.97 | 2.23E-08 |
|  | 8914470 | *EAMY_RS26495* | DUF1315 family protein | -1.63 | 2.28E-08 |
|  | 8912151 | *metA* | homoserine O-succinyltransferase | -1.54 | 2.30E-08 |
|  | 8914105 | *pal* | peptidoglycan-associated lipoprotein Pal | 1.80 | 2.34E-08 |
|  | 8912486 | *atpA* | F0F1 ATP synthase subunit alpha | 1.72 | 2.35E-08 |
|  | 8913691 | *ptsI* | phosphoenolpyruvate-protein phosphotransferase PtsI | 1.55 | 2.69E-08 |
|  | 8912463 | *artJ* | arginine ABC transporter substrate-binding protein | 1.82 | 3.15E-08 |
|  | 8914818 | *EAMY_RS24690* | YgdI/YgdR family lipoprotein | -2.10 | 3.61E-08 |
|  | 8911634 | *EAMY_RS25705* | helix-turn-helix domain-containing protein | 2.48 | 3.66E-08 |
|  | 8913829 | *rpsR* | 30S ribosomal protein S18 | 1.58 | 3.91E-08 |
|  | 55585574 | *EAMY_RS36430* | hypothetical protein | -2.63 | 4.74E-08 |
|  | 8913471 | *EAMY_RS28135* | hypothetical protein | 1.58 | 4.75E-08 |
|  | 8913885 | *rplB* | 50S ribosomal protein L2 | 1.53 | 4.96E-08 |
|  | 8914822 | *groL* | chaperonin GroEL | 2.82 | 5.14E-08 |
|  | 8911418 | *EAMY_RS21805* | cell envelope integrity TolA C-terminal domain-containing protein | -2.20 | 5.15E-08 |
|  | 8914583 | *tadA* | tRNA adenosine(34) deaminase TadA | 1.53 | 5.60E-08 |
|  | 8914697 | *zntR* | Zn(2+)-responsive transcriptional regulator | -1.53 | 5.61E-08 |
|  | 8914643 | *EAMY_RS20320* | UDP-3-O-(3-hydroxymyristoyl)glucosamine N-acyltransferase | -2.60 | 5.62E-08 |
|  | 8914084 | *EAMY_RS18575* | Rsd/AlgQ family anti-sigma factor | -2.08 | 5.93E-08 |
|  | 8913952 | *EAMY_RS27295* | tRNA-Asn | -4.76 | 6.64E-08 |
|  | 8914873 | *ghoS* | type V toxin-antitoxin system endoribonuclease antitoxin GhoS | -1.67 | 6.83E-08 |
|  | 8914254 | *rof* | Rho-binding antiterminator | -1.55 | 7.12E-08 |
|  | 8912522 | *tig* | trigger factor | 3.25 | 7.21E-08 |
|  | 8914698 | *rplQ* | 50S ribosomal protein L17 | 1.97 | 8.10E-08 |
|  | 8912847 | *fliD* | flagellar filament capping protein FliD | -2.40 | 8.83E-08 |
|  | 8911556 | *EAMY_RS24590* | putative holin | -2.43 | 9.15E-08 |
|  | 8912761 | *rnc* | ribonuclease III | -2.53 | 9.48E-08 |
|  | 23673330 | *EAMY_RS20630* | hypothetical protein | -2.86 | 9.48E-08 |
|  |  | *MSTRG.797.1* | unannotated transcript | -1.60 | 9.48E-08 |
|  | 8914043 | *dkgA* | 2,5-didehydrogluconate reductase DkgA | -1.98 | 1.06E-07 |
|  | 69102784 | *EAMY_RS36605* | DUF3142 domain-containing protein | -2.11 | 1.14E-07 |
|  |  | *MSTRG.2609.1* | unannotated transcript | -3.53 | 1.19E-07 |
|  | 8914091 | *EAMY_RS27825* | hypothetical protein | -2.62 | 1.39E-07 |
|  | 8912361 | *EAMY_RS26190* | hypothetical protein | -1.97 | 1.48E-07 |
|  | 41697620 | *EAMY_RS35955* | pseudo | -1.57 | 1.51E-07 |
|  | 8912923 | *EAMY_RS23970* | YccJ family protein | -3.41 | 1.51E-07 |
|  | 8913760 | *htpG* | molecular chaperone HtpG | 2.75 | 1.98E-07 |
|  | 8914318 | *EAMY_RS22835* | 2-thiouracil desulfurase family protein | -1.54 | 1.98E-07 |
|  | 23673323 | *EAMY_RS20225* | SprT family zinc-dependent metalloprotease | -1.80 | 2.13E-07 |
|  | 8914496 | *EAMY_RS26885* | MAPEG family protein | -1.54 | 2.25E-07 |
|  | 43837317 | *EAMY_RS36240* | hypothetical protein | 2.32 | 2.28E-07 |
|  | 8893782 | *EAMY_RS34430* | H-NS family nucleoid-associated regulatory protein | 1.61 | 2.47E-07 |
|  | 8913898 | *pth* | aminoacyl-tRNA hydrolase | -1.59 | 2.53E-07 |
|  | 55585553 | *EAMY_RS36325* | hypothetical protein | -1.78 | 2.56E-07 |
|  | 8911776 | *EAMY_RS27465* | Dabb family protein | -1.90 | 2.65E-07 |
|  | 8912974 | *EAMY_RS17890* | MIP/aquaporin family protein | -1.62 | 2.78E-07 |
|  | 8911655 | *EAMY_RS25880* | hypothetical protein | -1.94 | 2.81E-07 |
|  | 88183296 | *EAMY_RS36865* | pseudo | -2.12 | 3.70E-07 |
|  | 8912768 | *EAMY_RS24005* | GlpM family protein | -2.76 | 3.84E-07 |
|  | 8912433 | *creA* | protein CreA | 1.55 | 3.93E-07 |
|  | 8913883 | *rpmC* | 50S ribosomal protein L29 | 1.51 | 4.01E-07 |
|  | 8912397 | *EAMY_RS27445* | 2,3-butanediol dehydrogenase | 2.20 | 4.14E-07 |
|  | 8911820 | *ackA* | acetate kinase | 1.54 | 4.30E-07 |
|  | 8912237 | *EAMY_RS34165* | protein-tyrosine phosphatase family protein | -1.80 | 4.46E-07 |
|  | 8913010 | *EAMY_RS31960* | co-chaperone GroES | 2.52 | 4.61E-07 |
|  | 8911904 | *ung* | uracil-DNA glycosylase | 2.55 | 5.26E-07 |
|  | 8913908 | *EAMY_RS26095* | hypothetical protein | -2.88 | 5.60E-07 |
|  | 8914211 | *EAMY_RS24380* | mannosyl-3-phosphoglycerate phosphatase-related protein | -1.74 | 5.84E-07 |
|  | 8914674 | *nusA* | transcription termination factor NusA | 2.04 | 6.17E-07 |
|  | 8913235 | *EAMY_RS19710* | phytochelatin synthase family protein | 2.06 | 6.44E-07 |
|  | 8913157 | *ilvC* | ketol-acid reductoisomerase | 1.66 | 7.41E-07 |
|  |  | *MSTRG.1243.1* | unannotated transcript | -1.58 | 7.87E-07 |
|  | 8911770 | *EAMY_RS27385* | LysE family translocator | -1.91 | 8.34E-07 |
|  | 8914764 | *yidC* | membrane protein insertase YidC | 2.09 | 9.39E-07 |
|  | 8911543 | *EAMY_RS24350* | PrpF domain-containing protein | 1.80 | 1.05E-06 |
|  | 8911749 | *EAMY_RS27145* | acyltransferase family protein | 1.66 | 1.39E-06 |
|  | 8914432 | *EAMY_RS25810* | DUF2946 domain-containing protein | -2.03 | 1.57E-06 |
|  | 8911888 | *sseA* | 3-mercaptopyruvate sulfurtransferase | 1.66 | 1.73E-06 |
|  | 23673398 | *EAMY_RS30220* | hypothetical protein | -1.90 | 1.74E-06 |
|  | 8912107 | *EAMY_RS32245* | aspartate aminotransferase family protein | -1.94 | 1.74E-06 |
|  | 8912207 | *mntP* | manganese efflux pump MntP | 1.92 | 1.79E-06 |
|  | 30316911 | *EAMY_RS34980* | hypothetical protein | -1.87 | 1.92E-06 |
|  | 8913888 | *hslU* | HslU--HslV peptidase ATPase subunit | 2.35 | 1.93E-06 |
|  | 23673352 | *EAMY_RS24580* | hypothetical protein | -1.95 | 2.65E-06 |
|  | 8913659 | *EAMY_RS30005* | hypothetical protein | -2.12 | 2.82E-06 |
|  | 8912657 | *greA* | transcription elongation factor GreA | 1.73 | 3.08E-06 |
|  | 8911568 | *EAMY_RS24670* | flavocytochrome c | -1.94 | 3.25E-06 |
|  | 8912094 | *tssH* | type VI secretion system ATPase TssH | 1.62 | 3.28E-06 |
|  | 8911549 | *EAMY_RS24480* | antiterminator Q family protein | -2.03 | 3.81E-06 |
|  | 8912944 | *EAMY_RS21465* | hypothetical protein | 2.40 | 3.95E-06 |
|  | 30316846 | *EAMY_RS34655* | GIY-YIG nuclease family protein | -1.95 | 4.35E-06 |
|  | 8911931 | *EAMY_RS30350* | winged helix-turn-helix domain-containing protein | -1.85 | 4.50E-06 |
|  | 8911644 | *EAMY_RS25785* | acyltransferase | -1.66 | 4.63E-06 |
|  | 41697650 | *EAMY_RS36110* | pseudo | -1.52 | 6.13E-06 |
|  | 8913906 | *sapD* | putrescine export ABC transporter ATP-binding protein SapD | -1.60 | 6.31E-06 |
|  | 8911825 | *EAMY_RS28380* | ABC transporter permease | 1.62 | 6.33E-06 |
|  | 8913660 | *proW* | glycine betaine/L-proline ABC transporter permease ProW | -4.09 | 6.36E-06 |
|  | 30316904 | *EAMY_RS34945* | YqaE/Pmp3 family membrane protein | -1.82 | 7.43E-06 |
|  | 8911203 | *EAMY_RS17555* | reverse transcriptase family protein | -1.77 | 8.04E-06 |
|  | 8913144 | *EAMY_RS24575* | LPS 1,2-N-acetylglucosaminetransferase | -2.26 | 8.44E-06 |
|  | 8914120 | *murI* | glutamate racemase | 2.15 | 1.02E-05 |
|  | 8914535 | *EAMY_RS27985* | phosphatase PAP2 family protein | 1.68 | 1.04E-05 |
|  |  | *MSTRG.824.1* | unannotated transcript | -1.53 | 1.21E-05 |
|  | 8913810 | *rsxB* | electron transport complex subunit RsxB | -3.27 | 1.22E-05 |
|  | 8913341 | *EAMY_RS17410* | pseudo | -1.56 | 1.23E-05 |
|  | 8913825 | *rpsJ* | 30S ribosomal protein S10 | 1.68 | 1.26E-05 |
|  | 8914553 | *EAMY_RS28330* | ADP-ribosyltransferase | -1.80 | 1.32E-05 |
|  | 8912511 | *bioB* | biotin synthase BioB | -1.62 | 1.61E-05 |
|  | 30316899 | *rmf* | ribosome modulation factor | -1.60 | 1.61E-05 |
|  | 8914808 | *EAMY_RS32655* | CsbD family protein | -1.83 | 1.68E-05 |
|  | 8912682 | *EAMY_RS19090* | LLM class flavin-dependent oxidoreductase | 2.25 | 1.88E-05 |
|  | 8912710 | *dnaK* | molecular chaperone DnaK | 2.10 | 2.03E-05 |
|  | 8911600 | *araA* | L-arabinose isomerase | -1.57 | 2.22E-05 |
|  | 8911413 | *EAMY_RS21725* | PsiF family protein | -1.67 | 2.26E-05 |
|  | 8914426 | *EAMY_RS26105* | LysR family transcriptional regulator | -1.58 | 2.43E-05 |
|  | 8911351 | *xni* | flap endonuclease Xni | 1.92 | 2.48E-05 |
|  | 8914215 | *rffA* | dTDP-4-amino-4,6-dideoxygalactose transaminase | 1.86 | 2.49E-05 |
|  | 8914172 | *grpE* | nucleotide exchange factor GrpE | 2.74 | 2.49E-05 |
|  | 8913429 | *EAMY_RS19345* | helix-turn-helix transcriptional regulator | -2.25 | 2.50E-05 |
|  | 8914830 | *EAMY_RS33555* | LuxR C-terminal-related transcriptional regulator | -2.23 | 2.50E-05 |
|  | 8913576 | *dxs* | 1-deoxy-D-xylulose-5-phosphate synthase | 1.56 | 2.55E-05 |
|  | 8914065 | *tatC* | Sec-independent protein translocase subunit TatC | 1.55 | 2.64E-05 |
|  | 23673395 | *EAMY_RS29195* | pseudo | -1.71 | 2.91E-05 |
|  | 8913104 | *thpR* | RNA 2',3'-cyclic phosphodiesterase | -3.01 | 3.04E-05 |
|  | 8914513 | *EAMY_RS27260* | tRNA-Asn | -1.71 | 3.17E-05 |
|  | 55585554 | *EAMY_RS36330* | hypothetical protein | -1.68 | 3.34E-05 |
|  | 43837323 | *EAMY_RS36270* | pseudo | -1.55 | 3.74E-05 |
|  | 8913802 | *rffC* | dTDP-4-amino-4,6-dideoxy-D-galactose acyltransferase | 2.56 | 3.83E-05 |
|  | 8913653 | *EAMY_RS21620* | YbaN family protein | -1.72 | 3.95E-05 |
|  |  | *MSTRG.28.1* | unannotated transcript | -1.86 | 4.14E-05 |
|  | 8913805 | *lepB* | signal peptidase I | 1.63 | 4.39E-05 |
|  | 8912478 | *astB* | N-succinylarginine dihydrolase | -2.72 | 4.66E-05 |
|  | 23673399 | *can* | carbonate dehydratase | -2.01 | 4.78E-05 |
|  | 8913387 | *birA* | bifunctional biotin--[acetyl-CoA-carboxylase] ligase/biotin operon repressor BirA | 3.63 | 4.80E-05 |
|  | 8912667 | *EAMY_RS30145* | DUF3461 family protein | -1.80 | 5.54E-05 |
|  | 8912580 | *lon* | endopeptidase La | 1.60 | 5.59E-05 |
|  | 8911500 | *EAMY_RS23355* | hypothetical protein | -2.37 | 6.58E-05 |
|  | 8912063 | *EAMY_RS31885* | tRNA-Gly | -2.85 | 8.10E-05 |
|  | 8912870 | *fliM* | flagellar motor switch protein FliM | -2.22 | 8.31E-05 |
|  | 8911839 | *EAMY_RS28575* | hypothetical protein | -1.58 | 1.11E-04 |
|  | 8913696 | *EAMY_RS34155* | hypothetical protein | -1.77 | 1.17E-04 |
|  | 8911531 | *EAMY_RS24010* | DUF2594 family protein | -2.25 | 1.19E-04 |
|  | 30316988 | *pqqA* | pyrroloquinoline quinone precursor peptide PqqA | 2.38 | 1.19E-04 |
|  | 8912820 | *EAMY_RS24215* | flagellar basal body L-ring protein FlgH | 1.85 | 1.35E-04 |
|  | 23673387 | *EAMY_RS28935* | DUF1471 domain-containing protein | 1.81 | 1.62E-04 |
|  | 8911476 | *EAMY_RS23010* | RHS repeat-associated core domain-containing protein | 1.75 | 1.71E-04 |
|  | 8914246 | *sfsA* | DNA/RNA nuclease SfsA | -3.35 | 1.77E-04 |
|  | 8911933 | *cas2e* | type I-E CRISPR-associated endoribonuclease Cas2e | 2.74 | 1.82E-04 |
|  | 8911562 | *EAMY_RS24630* | GlsB/YeaQ/YmgE family stress response membrane protein | -1.85 | 2.37E-04 |
|  | 8911403 | *EAMY_RS21645* | hypothetical protein | -2.18 | 2.51E-04 |
|  | 8914241 | *pcnB* | polynucleotide adenylyltransferase PcnB | -1.86 | 2.66E-04 |
|  | 30316913 | *rprA* | ncRNA | 4.47 | 2.89E-04 |
|  |  | *MSTRG.2244.1* | unannotated transcript | -1.59 | 3.10E-04 |
|  | 8911802 | *EAMY_RS28085* | acyltransferase | -1.50 | 3.10E-04 |
|  | 8914506 | *fliZ* | flagella biosynthesis regulatory protein FliZ | -1.69 | 3.11E-04 |
|  |  | *MSTRG.79.1* | unannotated transcript | -1.62 | 3.37E-04 |
|  | 23673457 | *EAMY_RS34550* | pseudo | -2.29 | 3.61E-04 |
|  | 8913838 | *rplF* | 50S ribosomal protein L6 | 2.03 | 3.69E-04 |
|  | 8911367 | *EAMY_RS21150* | winged helix-turn-helix domain-containing protein | -1.57 | 4.19E-04 |
|  | 8913441 | *nrdI* | class Ib ribonucleoside-diphosphate reductase assembly flavoprotein NrdI | -1.52 | 4.19E-04 |
|  | 8912858 | *fliG* | flagellar motor switch protein FliG | -2.74 | 4.62E-04 |
|  | 8913734 | *dtd* | D-aminoacyl-tRNA deacylase | 2.96 | 5.22E-04 |
|  | 8913123 | *hslV* | ATP-dependent protease subunit HslV | 1.94 | 5.28E-04 |
|  | 8914061 | *bsmA* | biofilm peroxide resistance protein BsmA | -1.89 | 5.48E-04 |
|  | 8911525 | *hspQ* | heat shock protein HspQ | 1.65 | 5.50E-04 |
|  | 41697632 | *ypfM* | protein YpfM | -2.02 | 5.61E-04 |
|  | 8914133 | *rho* | transcription termination factor Rho | 1.61 | 5.82E-04 |
|  | 8911629 | *EAMY_RS25685* | barstar family protein | -1.63 | 6.00E-04 |
|  | 23673300 | *EAMY_RS17570* | hypothetical protein | -2.88 | 6.10E-04 |
|  | 30316919 | *EAMY_RS35020* | hypothetical protein | -1.86 | 6.12E-04 |
|  | 43500274 | *EAMY_RS36175* | hypothetical protein | -1.73 | 6.60E-04 |
|  | 8911237 | *secG* | preprotein translocase subunit SecG | -1.62 | 7.03E-04 |
|  | 8914295 | *ybbP* | putative ABC transporter permease subunit YbbP | 4.19 | 7.28E-04 |
|  | 8913907 | *sapF* | putrescine export ABC transporter ATP-binding protein SapF | -2.51 | 7.40E-04 |
|  | 8912874 | *fliO* | flagellar biosynthetic protein FliO | -1.69 | 8.16E-04 |
|  | 8913679 | *pstC* | phosphate ABC transporter permease PstC | -1.73 | 8.24E-04 |
|  | 8914592 | *clpB* | ATP-dependent chaperone ClpB | 1.61 | 1.02E-03 |
|  | 8912452 | *EAMY_RS21755* | YaiA family protein | -1.90 | 1.08E-03 |
|  | 8913295 | *aroC* | chorismate synthase | 1.53 | 1.14E-03 |
|  | 8912007 | *tssM* | type VI secretion system membrane subunit TssM | -2.61 | 1.14E-03 |
|  | 8911554 | *EAMY_RS24585* | GNAT family N-acetyltransferase | -2.36 | 1.25E-03 |
|  | 8913269 | *EAMY_RS33720* | siderophore-interacting protein | -1.51 | 1.30E-03 |
|  | 69102796 | *EAMY_RS36665* | pseudo | -1.57 | 1.33E-03 |
|  | 8913746 | *EAMY_RS28655* | tRNA-Arg | -2.70 | 1.40E-03 |
|  | 8911216 | *fabR* | HTH-type transcriptional repressor FabR | -1.68 | 1.57E-03 |
|  | 8911276 | *EAMY_RS19630* | spore coat U domain-containing protein | -1.52 | 1.58E-03 |
|  | 8913835 | *rpmD* | 50S ribosomal protein L30 | 1.90 | 1.59E-03 |
|  | 8913297 | *rrf* | 5S ribosomal RNA | -3.00 | 1.66E-03 |
|  | 8914659 | *tolC* | outer membrane channel protein TolC | 2.07 | 1.91E-03 |
|  | 8913968 | *EAMY_RS29560* | Tar ligand binding domain-containing protein | -1.82 | 2.05E-03 |
|  |  | *MSTRG.791.1* | unannotated transcript | -1.94 | 2.09E-03 |
|  | 8913464 | *EAMY_RS33585* | helix-turn-helix transcriptional regulator | -1.55 | 2.38E-03 |
|  | 8912854 | *fliE* | flagellar hook-basal body complex protein FliE | -2.81 | 2.67E-03 |
|  |  | *MSTRG.1230.1* | unannotated transcript | -1.68 | 2.81E-03 |
|  | 8913833 | *rpsI* | 30S ribosomal protein S9 | 2.69 | 2.83E-03 |
|  | 8914625 | *rlmM* | 23S rRNA (cytidine(2498)-2'-O)-methyltransferase RlmM | 2.25 | 2.92E-03 |
|  | 8914161 | *EAMY_RS27745* | phosphatase PAP2 family protein | 1.55 | 3.20E-03 |
|  | 8913839 | *EAMY_RS21270* | hypothetical protein | -1.69 | 3.39E-03 |
|  | 8912256 | *rrf* | 5S ribosomal RNA | -3.31 | 3.91E-03 |
|  | 8912009 | *tssK* | type VI secretion system baseplate subunit TssK | -2.39 | 4.33E-03 |
|  | 8912549 | *punC* | purine nucleoside transporter PunC | 3.47 | 4.64E-03 |
|  | 8913616 | *argE* | acetylornithine deacetylase | 1.87 | 4.67E-03 |
|  | 8911444 | *EAMY_RS22400* | tRNA-Arg | -1.52 | 4.91E-03 |
|  | 8913904 | *sapB* | putrescine export ABC transporter permease SapB | -1.78 | 5.78E-03 |
|  | 77383448 | *EAMY_RS36790* | hypothetical protein | -1.91 | 5.87E-03 |
|  | 8913891 | *rseB* | sigma-E factor regulatory protein RseB | -2.25 | 6.61E-03 |
|  | 30316889 | *EAMY_RS34870* | protein YohO | -1.64 | 7.26E-03 |
|  | 8913742 | *fliR* | flagellar biosynthetic protein FliR | -1.61 | 7.34E-03 |
|  | 8912247 | *rrf* | 5S ribosomal RNA | -2.51 | 7.75E-03 |
|  | 8913535 | *panC* | pantoate--beta-alanine ligase | -1.73 | 7.81E-03 |
|  | 69102808 | *kdpF* | K(+)-transporting ATPase subunit F | -2.29 | 7.84E-03 |
|  | 69102807 | *EAMY_RS36720* | pseudo | -1.71 | 7.94E-03 |
|  | 8912098 | *tssB* | type VI secretion system contractile sheath small subunit | 1.75 | 7.99E-03 |
|  | 8911990 | *tssF* | type VI secretion system baseplate subunit TssF | -1.87 | 8.38E-03 |
|  | 8913828 | *rpsH* | 30S ribosomal protein S8 | 2.63 | 8.81E-03 |
|  | 8912154 | *tauD* | taurine dioxygenase | 1.84 | 9.75E-03 |
|  | 8914834 | *lptG* | LPS export ABC transporter permease LptG | 3.15 | 9.84E-03 |
|  |  | *MSTRG.1122.1* | unannotated transcript | -1.67 | 9.91E-03 |
|  | 8914618 | *cas5e* | type I-E CRISPR-associated protein Cas5/CasD | 1.91 | 9.96E-03 |
|  | 8912927 | *galT* | galactose-1-phosphate uridylyltransferase | 1.77 | 1.10E-02 |
|  | 8912872 | *fliN* | flagellar motor switch protein FliN | -1.87 | 1.33E-02 |
|  | 8912577 | *tssG* | type VI secretion system baseplate subunit TssG | -1.76 | 1.34E-02 |
|  | 8911246 | *EAMY_RS19245* | restriction endonuclease | -2.11 | 1.38E-02 |
|  | 8913216 | *rrf* | 5S ribosomal RNA | -3.18 | 1.51E-02 |
|  | 8913633 | *pqqD* | pyrroloquinoline quinone biosynthesis peptide chaperone PqqD | -1.97 | 1.73E-02 |
|  | 8912253 | *rrf* | 5S ribosomal RNA | -1.61 | 1.91E-02 |
|  | 8913409 | *nagB* | glucosamine-6-phosphate deaminase | 3.02 | 2.06E-02 |
|  | 8911573 | *EAMY_RS24825* | acyl carrier protein | -1.87 | 2.08E-02 |
|  | 8912661 | *EAMY_RS34020* | SemiSWEET family transporter | -1.67 | 2.09E-02 |
|  | 8911989 | *tssH* | type VI secretion system ATPase TssH | -1.56 | 2.20E-02 |
|  | 8913462 | *thiL* | thiamine-phosphate kinase | -1.75 | 2.21E-02 |
|  | 8912229 | *EAMY_RS34085* | winged helix-turn-helix domain-containing protein | -2.54 | 2.31E-02 |
|  | 8913536 | *panD* | aspartate 1-decarboxylase | -1.97 | 2.32E-02 |
|  | 8913179 | *EAMY_RS24750* | HrpJ domain-containing protein | -1.58 | 2.40E-02 |
|  | 8913091 | *EAMY_RS21005* | hypothetical protein | -1.75 | 2.42E-02 |
|  | 8913353 | *flhC* | flagellar transcriptional regulator FlhC | -2.30 | 2.44E-02 |
|  | 8913510 | *EAMY_RS27310* | hypothetical protein | -1.59 | 2.49E-02 |
|  | 8913661 | *proX* | glycine betaine/L-proline ABC transporter substrate-binding protein ProX | -1.59 | 2.55E-02 |
|  | 8914905 | *EAMY_RS20205* | YqgE/AlgH family protein | -3.29 | 2.65E-02 |
|  | 8912005 | *tssA* | type VI secretion system protein TssA | -1.61 | 2.65E-02 |
|  | 8914500 | *cmoB* | tRNA 5-methoxyuridine(34)/uridine 5-oxyacetic acid(34) synthase CmoB | 2.51 | 2.76E-02 |
|  | 8912006 | *tagF* | type VI secretion system-associated protein TagF | -1.70 | 2.78E-02 |
|  | 8914036 | *sucD* | succinate--CoA ligase subunit alpha | 2.09 | 3.07E-02 |
|  | 8913354 | *motA* | flagellar motor stator protein MotA | -2.48 | 3.08E-02 |
|  | 8914960 | *EAMY_RS32835* | DUF1992 domain-containing protein | -2.55 | 3.28E-02 |
|  | 8914203 | *rfaQ* | putative lipopolysaccharide heptosyltransferase III | -2.79 | 3.33E-02 |
|  | 8913233 | *thyA* | thymidylate synthase | 4.12 | 3.65E-02 |
|  | 8911275 | *ftsP* | cell division protein FtsP | -2.19 | 3.81E-02 |
|  | 8914302 | *purK* | 5-(carboxyamino)imidazole ribonucleotide synthase | -1.68 | 3.90E-02 |
|  | 8914214 | *rfbB* | dTDP-glucose 4,6-dehydratase | 2.63 | 3.95E-02 |
|  | 8912791 | *flgN* | flagellar export chaperone FlgN | -1.53 | 3.99E-02 |
|  | 8914306 | *EAMY_RS32595* | phage holin, lambda family | -1.67 | 4.33E-02 |
|  | 23673375 | *EAMY_RS28080* | hypothetical protein | -1.63 | 4.66E-02 |
|  | 8913763 | *EAMY_RS30215* | SymE family type I addiction module toxin | -1.86 | 4.67E-02 |
|  | 8912242 | *phoU* | phosphate signaling complex protein PhoU | -1.66 | 4.68E-02 |
|  | 8912203 | *EAMY_RS33785* | DUF1471 domain-containing protein | 1.69 | 4.76E-02 |

^a^ Gene IDs, locus tags, symbols and functions obtained from the DAVID database for *E. amylovora* CFBP 1430. MSTRG identifiers represent assembled and annotated by StringTie, which were not previously annotated in CFBP 1430 strain. They could be potentially new transcripts, alternative splice variants, or transcripts specific to the experimental conditions.

**Table S5**. DEGs in EaR2 with respect to Ea273 log-phase cells exposed to copper shock

| **GeneID** | **Symbol** | **GeneName** | **Gene Function** | **Log_2_FC** | **FDRw** |
| --- | --- | --- | --- | --- | --- |
| 8912016 | *EAMY_RS31370* | EAMY_RS31370 | PepSY-associated TM helix domain-containing protein | 2.45 | 6.42E-03 |
| 8914591 | *trxC* | EAMY_RS29915 | thioredoxin TrxC | -1.45 | 3.34E-02 |

**Table S6**. DEGs in EaR2 with respect to Ea273 cells grown with 1 mM CuSO_4_ up to mid-log phase

| **Gene ID** | **Symbol** | **Gene Function** | **logFC** | **adj.P.Val** |
| --- | --- | --- | --- | --- |
| 8912479 | *spy* | ATP-independent periplasmic protein-refolding chaperone Spy | 5.59 | 1.70E-15 |
| 8912357 | *EAMY_RS31520* | SulP family inorganic anion transporter | 3.46 | 5.03E-14 |
| 8912412 | *wbaP* | undecaprenyl-phosphate galactose phosphotransferase WbaP | 3.14 | 3.93E-13 |
| 8912406 | *EAMY_RS27705* | polysaccharide biosynthesis tyrosine autokinase | 2.46 | 1.41E-12 |
| 8913418 | *EAMY_RS24365* | glycine zipper 2TM domain-containing protein | 2.74 | 3.12E-11 |
| 8914463 | *ppsA* | phosphoenolpyruvate synthase | 2.75 | 1.18E-10 |
| 8911811 | *kup* | low affinity potassium transporter Kup | -2.69 | 1.36E-10 |
| 8914077 | *ybbA* | putative ABC transporter ATP-binding protein YbbA | 3.06 | 2.47E-10 |
| 8912415 | *wcaK* | colanic acid biosynthesis pyruvyl transferase WcaK | 1.81 | 2.47E-10 |
| 8912414 | *EAMY_RS27680* | phage tailspike protein | 2.03 | 2.47E-10 |
| 8912405 | *EAMY_RS27710* | protein-tyrosine-phosphatase | 2.38 | 2.47E-10 |
| 8912413 | *EAMY_RS27715* | polysaccharide export protein | 1.76 | 2.47E-10 |
| 8912926 | *EAMY_RS27665* | lipopolysaccharide biosynthesis protein | 1.98 | 1.32E-09 |
| 8913587 | *efeB* | iron uptake transporter deferrochelatase/peroxidase subunit | -1.94 | 1.41E-09 |
| 8914661 | *EAMY_RS19450* | glutathionylspermidine synthase family protein | 2.24 | 1.29E-08 |
| 8914129 | *sltY* | murein transglycosylase | 1.63 | 2.39E-08 |
| 8914837 | *EAMY_RS19810* | carbon starvation CstA family protein | 1.51 | 3.67E-08 |
| 8912404 | *ampC* | class C beta-lactamase | 2.00 | 8.05E-08 |
| 8912408 | *EAMY_RS27695* | EpsG family protein | 2.80 | 1.16E-07 |
| 8913978 | *EAMY_RS33715* | LysR substrate-binding domain-containing protein | -1.60 | 1.32E-07 |
| 8913479 | *EAMY_RS26275* | YciC family protein | 2.22 | 1.64E-07 |
| 8912472 | *ompC* | porin OmpC | -1.81 | 1.67E-07 |
| 8914716 | *EAMY_RS33110* | aspartate aminotransferase family protein | 1.70 | 2.17E-07 |
| 8913687 | *ychH* | stress-induced protein YchH | -2.12 | 2.87E-07 |
| 8914406 | *emtA* | membrane-bound lytic murein transglycosylase EmtA | 2.29 | 4.16E-07 |
| 8913785 | *acnA* | aconitate hydratase AcnA | 1.78 | 4.24E-07 |
| 8914055 | *EAMY_RS34145* | OmpA family lipoprotein | 1.94 | 4.24E-07 |
| 8912410 | *EAMY_RS27685* | glycosyltransferase | 1.98 | 4.25E-07 |
| 8911738 | *EAMY_RS27055* | flippase | 2.47 | 4.47E-07 |
| 8914346 | *cspD* | cold shock-like protein CspD | -2.23 | 6.37E-07 |
|  | *MSTRG.363.1* | Unannotated transcript | 1.68 | 6.37E-07 |
| 8912711 | *EAMY_RS30930* | acyltransferase | 2.07 | 2.15E-06 |
| 8912409 | *EAMY_RS27690* | glycosyltransferase family 4 protein | 2.03 | 2.16E-06 |
| 8911543 | *EAMY_RS24350* | PrpF domain-containing protein | 2.88 | 3.09E-06 |
| 8911807 | *ompC* | porin OmpC | 1.70 | 3.16E-06 |
| 8911433 | *EAMY_RS22225* | NUDIX domain-containing protein | -1.78 | 3.27E-06 |
| 8914796 | *ftnA* | non-heme ferritin | -1.50 | 4.60E-06 |
| 8912990 | *EAMY_RS34040* | NAD(P)-dependent alcohol dehydrogenase | 1.51 | 4.60E-06 |
| 8912812 | *flgD* | flagellar hook assembly protein FlgD | -2.29 | 5.90E-06 |
| 8912814 | *flgE* | flagellar hook protein FlgE | -2.34 | 6.09E-06 |
| 8914018 | *srlD* | sorbitol-6-phosphate dehydrogenase | 1.98 | 6.66E-06 |
| 8913053 | *hutU* | urocanate hydratase | 2.50 | 8.69E-06 |
| 8912407 | *EAMY_RS27700* | glycosyltransferase family 2 protein | 2.01 | 8.69E-06 |
| 8912359 | *aceE* | pyruvate dehydrogenase (acetyl-transferring), homodimeric type | -1.51 | 8.69E-06 |
| 30316917 | *EAMY_RS35010* | glycine zipper 2TM domain-containing protein | 2.54 | 8.69E-06 |
| 8912818 | *flgG* | flagellar basal-body rod protein FlgG | -2.00 | 1.00E-05 |
| 8911759 | *EAMY_RS27275* | metal ABC transporter substrate-binding protein | -1.54 | 1.00E-05 |
| 8913299 | *exbB* | tol-pal system-associated acyl-CoA thioesterase | 2.80 | 1.49E-05 |
| 8911620 | *EAMY_RS25605* | GNAT family N-acetyltransferase | 1.55 | 1.60E-05 |
| 8914128 | *EAMY_RS22220* | hypothetical protein | -1.51 | 1.62E-05 |
| 8914792 | *fpr* | ferredoxin--NADP(+) reductase | 2.54 | 1.92E-05 |
| 8912391 | *budA* | acetolactate decarboxylase | -1.69 | 2.11E-05 |
| 8911203 | *EAMY_RS17555* | reverse transcriptase family protein | -2.58 | 2.11E-05 |
| 8913139 | *hutH* | histidine ammonia-lyase | 2.67 | 2.11E-05 |
| 8913135 | *hutI* | imidazolonepropionase | 1.88 | 2.11E-05 |
| 8914405 | *wbaP* | undecaprenyl-phosphate galactose phosphotransferase WbaP | 1.51 | 2.11E-05 |
| 8914376 | *efeO* | iron uptake system protein EfeO | -1.65 | 2.11E-05 |
| 8912847 | *fliD* | flagellar filament capping protein FliD | -1.75 | 2.11E-05 |
| 8912816 | *EAMY_RS24205* | flagellar basal body rod protein FlgF | -2.01 | 2.50E-05 |
| 60596820 | *EAMY_RS36490* | pseudo | -1.74 | 2.66E-05 |
| 8913963 | *glnK* | P-II family nitrogen regulator | -2.65 | 3.70E-05 |
| 8912810 | *flgC* | flagellar basal body rod protein FlgC | -2.65 | 5.33E-05 |
| 8911749 | *EAMY_RS27145* | acyltransferase family protein | 2.87 | 5.47E-05 |
| 8913269 | *EAMY_RS33720* | siderophore-interacting protein | 3.06 | 6.07E-05 |
| 8913471 | *EAMY_RS28135* | hypothetical protein | 1.84 | 1.16E-04 |
| 8912719 | *dppA* | dipeptide ABC transporter periplasmic-binding protein DppA | -1.59 | 1.18E-04 |
| 8912003 | *tssC* | type VI secretion system contractile sheath large subunit | -2.72 | 1.19E-04 |
| 8911546 | *rcsA* | transcriptional regulator RcsA | 1.80 | 1.29E-04 |
| 8914466 | *EAMY_RS25135* | heme ABC transporter ATP-binding protein | 2.08 | 1.29E-04 |
| 8912422 | *folA* | type 3 dihydrofolate reductase | 1.67 | 1.38E-04 |
| 8913318 | *EAMY_RS29135* | MFS transporter | 1.53 | 1.99E-04 |
| 69102765 | *EAMY_RS36510* | GIY-YIG nuclease family protein | -1.70 | 2.08E-04 |
| 8913157 | *ilvC* | ketol-acid reductoisomerase | -1.73 | 2.32E-04 |
| 8913134 | *EAMY_RS23320* | HutD family protein | 2.42 | 2.38E-04 |
| 8912808 | *flgB* | flagellar basal body rod protein FlgB | -2.45 | 2.58E-04 |
| 8914068 | *EAMY_RS33075* | LysE family translocator | -1.71 | 3.30E-04 |
| 8911658 | *asr* | acid resistance repetitive basic protein Asr | 2.09 | 3.31E-04 |
| 8914660 | *EAMY_RS19455* | DUF1190 family protein | 1.83 | 3.31E-04 |
| 23673387 | *EAMY_RS28935* | DUF1471 domain-containing protein | 3.76 | 3.92E-04 |
| 8913976 | *soxS* | superoxide response transcriptional regulator SoxS | 1.68 | 4.01E-04 |
| 8912016 | *EAMY_RS31370* | PepSY-associated TM helix domain-containing protein | 2.57 | 4.93E-04 |
| 8912004 | *tssB* | type VI secretion system contractile sheath small subunit | -2.88 | 4.99E-04 |
| 8913570 | *EAMY_RS17820* | sulfate ABC transporter substrate-binding protein | -2.62 | 9.79E-04 |
| 8912764 | *EAMY_RS19620* | MFS transporter | 1.51 | 9.79E-04 |
| 8911568 | *EAMY_RS24670* | flavocytochrome c | -2.01 | 1.11E-03 |
| 8914432 | *EAMY_RS25810* | DUF2946 domain-containing protein | -1.69 | 1.15E-03 |
| 8913660 | *proW* | glycine betaine/L-proline ABC transporter permease ProW | -3.25 | 1.70E-03 |
| 8913386 | *EAMY_RS18960* | helix-turn-helix domain-containing protein | -1.97 | 2.29E-03 |
| 8913783 | *cbl* | HTH-type transcriptional regulator Cbl | -2.17 | 2.53E-03 |
| 8913441 | *nrdI* | class Ib ribonucleoside-diphosphate reductase assembly flavoprotein NrdI | -1.52 | 2.87E-03 |
| 23673363 | *adhE* | pseudo | -1.89 | 2.97E-03 |
| 8893804 | *EAMY_RS34525* | class I SAM-dependent methyltransferase | -1.54 | 3.28E-03 |
| 8911567 | *EAMY_RS24665* | anion permease | -2.08 | 3.39E-03 |
| 8911776 | *EAMY_RS27465* | Dabb family protein | -1.66 | 3.78E-03 |
| 8913766 | *EAMY_RS19435* | accessory factor UbiK family protein | -1.91 | 3.81E-03 |
| 8914036 | *sucD* | succinate--CoA ligase subunit alpha | 5.71 | 4.33E-03 |
| 8912746 | *EAMY_RS28240* | MFS transporter | 1.60 | 4.60E-03 |
| 8912522 | *tig* | trigger factor | 2.23 | 5.03E-03 |
| 8912768 | *EAMY_RS24005* | GlpM family protein | -2.69 | 5.97E-03 |
| 8912577 | *tssG* | type VI secretion system baseplate subunit TssG | -2.50 | 6.12E-03 |
| 69102775 | *EAMY_RS36560* | YnfU family zinc-binding protein | -2.36 | 6.12E-03 |
| 8913679 | *pstC* | phosphate ABC transporter permease PstC | 2.69 | 6.84E-03 |
| 8911777 | *EAMY_RS27470* | DUF2000 domain-containing protein | -1.78 | 7.46E-03 |
| 55585552 | *EAMY_RS36320* | hypothetical protein | 4.03 | 7.47E-03 |
| 77383450 | *EAMY_RS36800* | hypothetical protein | 2.88 | 7.72E-03 |
| 8912984 | *gltK* | glutamate/aspartate ABC transporter permease GltK | 2.83 | 7.87E-03 |
| 8913811 | *rsxC* | electron transport complex subunit RsxC | 1.81 | 8.28E-03 |
| 30316913 | *rprA* | ncRNA | 4.76 | 8.73E-03 |
| 8914070 | *tauB* | taurine ABC transporter ATP-binding subunit | -2.46 | 9.06E-03 |
| 8911358 | *cysG* | siroheme synthase CysG | -1.70 | 9.58E-03 |
| 8913850 | *metJ* | met regulon transcriptional regulator MetJ | -1.50 | 1.01E-02 |
| 8914827 | *nrdG* | anaerobic ribonucleoside-triphosphate reductase-activating protein | -1.64 | 1.01E-02 |
| 8913001 | *rpe* | ribulose-phosphate 3-epimerase | -5.39 | 1.23E-02 |
| 8912900 | *fruB* | fused PTS fructose transporter subunit IIA/HPr protein | -2.17 | 1.26E-02 |
| 8912641 | *cysD* | sulfate adenylyltransferase subunit CysD | -2.07 | 1.49E-02 |
| 8912007 | *tssM* | type VI secretion system membrane subunit TssM | -2.46 | 1.60E-02 |
| 8912859 | *fliH* | flagellar assembly protein FliH | -1.63 | 1.63E-02 |
| 23673330 | *EAMY_RS20630* | hypothetical protein | -1.73 | 1.79E-02 |
| 8912616 | *EAMY_RS19650* | spore coat protein U domain-containing protein | -2.65 | 2.35E-02 |
| 8913616 | *argE* | acetylornithine deacetylase | 1.62 | 2.39E-02 |
| 8913133 | *ppiD* | peptidylprolyl isomerase | 1.76 | 2.40E-02 |
| 8912009 | *tssK* | type VI secretion system baseplate subunit TssK | -2.32 | 2.44E-02 |
| 8912005 | *tssA* | type VI secretion system protein TssA | -1.90 | 2.47E-02 |
| 8914491 | *EAMY_RS26715* | PqiB family protein | -1.78 | 2.57E-02 |
| 8912396 | *EAMY_RS20745* | tRNA-Met | -1.60 | 2.68E-02 |
| 8911262 | *ribB* | 3,4-dihydroxy-2-butanone-4-phosphate synthase | 3.01 | 2.80E-02 |
| 8912618 | *EAMY_RS19640* | molecular chaperone | -1.94 | 2.81E-02 |
| 8911276 | *EAMY_RS19630* | spore coat U domain-containing protein | -1.92 | 2.98E-02 |
| 8911989 | *tssH* | type VI secretion system ATPase TssH | -1.75 | 3.11E-02 |
| 8911356 | *queD* | 6-carboxytetrahydropterin synthase QueD | -1.60 | 3.18E-02 |
| 8914295 | *ybbP* | putative ABC transporter permease subunit YbbP | 4.04 | 3.22E-02 |
| 8912639 | *cysN* | sulfate adenylyltransferase subunit CysN | -1.64 | 3.37E-02 |
| 8913429 | *EAMY_RS19345* | helix-turn-helix transcriptional regulator | -1.80 | 3.50E-02 |
| 8912429 | *osmB* | osmotically-inducible lipoprotein OsmB | 2.17 | 3.72E-02 |
| 8912154 | *tauD* | taurine dioxygenase | -1.60 | 3.73E-02 |

^a^ Gene IDs, locus tags, symbols and functions obtained from the DAVID database for *E. amylovora* CFBP 1430. MSTRG identifiers represent assembled and annotated by StringTie, which were not previously annotated in CFBP 1430 strain. They could be potentially new transcripts, alternative splice variants, or transcripts specific to the experimental conditions.

**Table S7**. DEGs in EaR2 with respect to Ea273 cells grown with 1 mM CuSO_4_ up to stationary phase^a^

| **Gene ID** | ***Symbol*** | **Gene Function** | **logFC** | **FDR** |
| --- | --- | --- | --- | --- |
| 8912415 | *wcaK* | colanic acid biosynthesis pyruvyl transferase WcaK | 3.95 | 1.48E-16 |
| 8912414 | *EAMY_RS27680* | phage tailspike protein | 3.97 | 1.63E-16 |
| 8912479 | *spy* | ATP-independent periplasmic protein-refolding chaperone Spy | 5.82 | 1.76E-16 |
| 8913418 | *EAMY_RS24365* | glycine zipper 2TM domain-containing protein | 4.70 | 2.12E-16 |
| 8912357 | *EAMY_RS31520* | SulP family inorganic anion transporter | 4.04 | 2.12E-16 |
| 8912413 | *EAMY_RS27715* | polysaccharide export protein | 3.34 | 2.17E-16 |
| 8912406 | *EAMY_RS27705* | polysaccharide biosynthesis tyrosine autokinase | 3.26 | 5.27E-16 |
| 8912926 | *EAMY_RS27665* | lipopolysaccharide biosynthesis protein | 3.25 | 9.82E-15 |
| 8912412 | *wbaP* | undecaprenyl-phosphate galactose phosphotransferase WbaP | 3.64 | 1.18E-14 |
| 8913587 | *efeB* | iron uptake transporter deferrochelatase/peroxidase subunit | -3.39 | 1.58E-14 |
| 8914378 | *efeU* | iron uptake transporter permease EfeU | -3.94 | 5.27E-13 |
| 8914463 | *ppsA* | phosphoenolpyruvate synthase | 3.23 | 6.29E-13 |
| 8911811 | *kup* | low affinity potassium transporter Kup | -3.49 | 7.33E-13 |
| 8914055 | *EAMY_RS34145* | OmpA family lipoprotein | 4.01 | 1.16E-12 |
| 8912405 | *EAMY_RS27710* | protein-tyrosine-phosphatase | 3.32 | 1.33E-12 |
| 8913162 | *EAMY_RS33810* | serralysin family metalloprotease | -2.95 | 1.74E-12 |
| 8914376 | *efeO* | iron uptake system protein EfeO | -3.85 | 3.59E-12 |
| 8911620 | *EAMY_RS25605* | GNAT family N-acetyltransferase | 3.15 | 7.01E-12 |
| 8912410 | *EAMY_RS27685* | glycosyltransferase | 3.92 | 7.18E-12 |
| 30316917 | *EAMY_RS35010* | glycine zipper 2TM domain-containing protein | 3.98 | 4.75E-11 |
| 8912695 | *EAMY_RS32250* | lysine N(6)-hydroxylase/L-ornithine N(5)-oxygenase family protein | -3.89 | 7.98E-11 |
| 8914406 | *emtA* | membrane-bound lytic murein transglycosylase EmtA | 3.49 | 1.64E-10 |
| 8912711 | *EAMY_RS30930* | acyltransferase | 3.82 | 1.98E-10 |
| 8914174 | *ispC* | 1-deoxy-D-xylulose-5-phosphate reductoisomerase | 2.61 | 2.58E-10 |
| 8911284 | *EAMY_RS19705* | disulfide bond formation protein B | 3.70 | 3.37E-10 |
| 8913471 | *EAMY_RS28135* | hypothetical protein | 3.22 | 4.81E-10 |
| 8911849 | *EAMY_RS28685* | hypothetical protein | 3.60 | 6.92E-10 |
| 8912407 | *EAMY_RS27700* | glycosyltransferase family 2 protein | 3.66 | 7.95E-10 |
| 8912409 | *EAMY_RS27690* | glycosyltransferase family 4 protein | 3.94 | 8.51E-10 |
| 8914405 | *wbaP* | undecaprenyl-phosphate galactose phosphotransferase WbaP | 2.51 | 1.00E-09 |
| 8914346 | *cspD* | cold shock-like protein CspD | -3.32 | 1.11E-09 |
| 8911267 | *EAMY_RS19515* | aminotransferase class III-fold pyridoxal phosphate-dependent enzyme | -2.10 | 1.31E-09 |
| 8912693 | *EAMY_RS32255* | GNAT family N-acetyltransferase | -3.75 | 1.31E-09 |
| 8912198 | *EAMY_RS33740* | SMP-30/gluconolactonase/LRE family protein | 2.05 | 1.31E-09 |
| 8914129 | *sltY* | murein transglycosylase | 1.78 | 1.42E-09 |
| 8912510 | *EAMY_RS23225* | suppressor of fused domain protein | 2.81 | 1.64E-09 |
| 8912924 | *galF* | UTP--glucose-1-phosphate uridylyltransferase GalF | 2.09 | 1.81E-09 |
| 8913630 | *EAMY_RS31515* | dipeptidase | 2.35 | 1.98E-09 |
| 8912408 | *EAMY_RS27695* | EpsG family protein | 3.81 | 2.21E-09 |
| 8912994 | *EAMY_RS33745* | hypothetical protein | 2.39 | 2.21E-09 |
| 8911546 | *rcsA* | transcriptional regulator RcsA | 3.79 | 2.24E-09 |
| 8912404 | *ampC* | class C beta-lactamase | 2.22 | 3.67E-09 |
| 8914804 | *EAMY_RS32695* | YjbH domain-containing protein | 1.68 | 4.98E-09 |
| 8914077 | *ybbA* | putative ABC transporter ATP-binding protein YbbA | 2.38 | 9.00E-09 |
| 8912683 | *truA* | tRNA pseudouridine(38-40) synthase TruA | 1.76 | 9.50E-09 |
| 8911738 | *EAMY_RS27055* | flippase | 2.64 | 1.30E-08 |
| 8914837 | *EAMY_RS19810* | carbon starvation CstA family protein | 1.54 | 1.34E-08 |
| 8914661 | *EAMY_RS19450* | glutathionylspermidine synthase family protein | 2.23 | 1.71E-08 |
| 8913626 | *EAMY_RS19540* | type I polyketide synthase | -1.63 | 2.89E-08 |
| 8913497 | *EAMY_RS20015* | HrpW-specific chaperone | 2.28 | 3.39E-08 |
| 8914052 | *EAMY_RS19535* | type I polyketide synthase | -2.14 | 8.68E-08 |
| 8913942 | *EAMY_RS20190* | thioredoxin family protein | 2.00 | 8.68E-08 |
| 8912732 | *EAMY_RS20195* | peroxiredoxin | 1.89 | 8.68E-08 |
| 8912694 | *EAMY_RS32260* | TonB-dependent siderophore receptor | -1.61 | 1.40E-07 |
| 8914716 | *EAMY_RS33110* | aspartate aminotransferase family protein | 1.61 | 1.58E-07 |
| 8914851 | *EAMY_RS23485* | serine hydrolase | 1.59 | 1.63E-07 |
| 8913281 | *EAMY_RS19525* | beta-ketoacyl synthase N-terminal-like domain-containing protein | -1.94 | 1.65E-07 |
| 8913687 | *ychH* | stress-induced protein YchH | -2.06 | 2.07E-07 |
| 8911268 | *EAMY_RS19545* | condensation domain-containing protein | -1.52 | 2.58E-07 |
| 8914619 | *cas7e* | type I-E CRISPR-associated protein Cas7/Cse4/CasC | -1.90 | 2.77E-07 |
| 69102764 | *EAMY_RS36505* | DUF5993 family protein | 3.99 | 2.85E-07 |
| 8911735 | *flhC* | flagellar transcriptional regulator FlhC | -1.66 | 4.80E-07 |
| 8912107 | *EAMY_RS32245* | aspartate aminotransferase family protein | -3.59 | 6.23E-07 |
| 8913280 | *EAMY_RS19520* | polyketide synthase | -1.76 | 2.77E-06 |
| 8911749 | *EAMY_RS27145* | acyltransferase family protein | 2.78 | 3.08E-06 |
| 8911759 | *EAMY_RS27275* | metal ABC transporter substrate-binding protein | -1.71 | 3.65E-06 |
| 8913479 | *EAMY_RS26275* | YciC family protein | 1.73 | 4.74E-06 |
| 8911706 | *EAMY_RS26685* | YobH family protein | 1.97 | 6.37E-06 |
| 23673438 | *EAMY_RS33805* | protease inhibitor Inh/omp19 family protein | -2.11 | 7.46E-06 |
|  | *MSTRG.1974.1* | Unannotated transcript | -1.98 | 7.52E-06 |
| 8913118 | *hrpN* | type III secretion system harpin HrpN | 2.18 | 9.10E-06 |
| 8913101 | *EAMY_RS19955* | EscI/YscI/HrpB family type III secretion system inner rod protein | 2.51 | 1.46E-05 |
| 8913121 | *EAMY_RS19935* | sigma 54-interacting transcriptional regulator | 2.04 | 1.50E-05 |
| 8911612 | *EAMY_RS25565* | hypothetical protein | -1.75 | 1.78E-05 |
| 8913333 | *EAMY_RS33535* | AI-2E family transporter | 1.69 | 2.02E-05 |
| 8911264 | *EAMY_RS19500* | creatininase family protein | -1.53 | 2.09E-05 |
| 8911614 | *EAMY_RS25570* | TonB-dependent receptor | -1.51 | 2.46E-05 |
| 8914651 | *EAMY_RS20185* | sigma-70 family RNA polymerase sigma factor | 1.60 | 2.58E-05 |
| 8913269 | *EAMY_RS33720* | siderophore-interacting protein | -5.13 | 2.69E-05 |
| 8914614 | *cas6e* | type I-E CRISPR-associated protein Cas6/Cse3/CasE | -1.71 | 2.83E-05 |
| 8912225 | *EAMY_RS34025* | acyltransferase | 1.82 | 2.87E-05 |
| 8913529 | *ppiA* | peptidylprolyl isomerase A | 1.72 | 3.07E-05 |
| 8912422 | *folA* | type 3 dihydrofolate reductase | 1.71 | 3.31E-05 |
| 8913639 | *EAMY_RS21045* | helix-turn-helix domain-containing protein | 2.06 | 3.95E-05 |
| 8911266 | *EAMY_RS19510* | nucleoside 2-deoxyribosyltransferase | -1.69 | 4.14E-05 |
| 8911543 | *EAMY_RS24350* | PrpF domain-containing protein | 2.73 | 4.95E-05 |
| 8914660 | *EAMY_RS19455* | DUF1190 family protein | 2.24 | 5.55E-05 |
| 8911640 | *EAMY_RS25755* | ABC transporter substrate-binding protein | 1.62 | 6.22E-05 |
| 8913134 | *EAMY_RS23320* | HutD family protein | 1.85 | 6.55E-05 |
| 8913786 | *ygiD* | 4,5-DOPA dioxygenase extradiol | 1.57 | 6.68E-05 |
| 8914416 | *EAMY_RS26280* | septation protein A | 1.82 | 7.16E-05 |
| 8913669 | *EAMY_RS33795* | HlyD family type I secretion periplasmic adaptor subunit | -1.55 | 8.26E-05 |
| 8911281 | *EAMY_RS19685* | ABC transporter substrate-binding protein | -1.51 | 8.38E-05 |
| 8913139 | *hutH* | histidine ammonia-lyase | 1.89 | 8.72E-05 |
| 8913504 | *EAMY_RS19950* | Hrp pili protein HrpA | 3.52 | 9.90E-05 |
| 8911508 | *EAMY_RS23440* | sugar porter family MFS transporter | 2.86 | 9.90E-05 |
| 8914944 | *EAMY_RS28760* | YfeC-like transcriptional regulator | -2.00 | 1.04E-04 |
| 8913439 | *proV* | glycine betaine/L-proline ABC transporter ATP-binding protein ProV | -1.88 | 1.70E-04 |
| 8912174 | *iolC* | 5-dehydro-2-deoxygluconokinase | 2.05 | 2.03E-04 |
| 8911760 | *EAMY_RS27305* | protein kinase family protein | -1.51 | 3.67E-04 |
| 8914722 | *EAMY_RS33475* | zinc/cadmium/mercury/lead-transporting ATPase | -1.60 | 3.67E-04 |
| 8913235 | *EAMY_RS19710* | phytochelatin synthase family protein | 2.27 | 4.65E-04 |
| 8913053 | *hutU* | urocanate hydratase | 1.61 | 4.66E-04 |
| 8913184 | *iolD* | 3D-(3,5/4)-trihydroxycyclohexane-1,2-dione acylhydrolase (decyclizing) | 1.70 | 8.00E-04 |
| 8913299 | *exbB* | tol-pal system-associated acyl-CoA thioesterase | 1.93 | 1.29E-03 |
| 8912648 | *cysC* | adenylyl-sulfate kinase | 1.93 | 1.61E-03 |
| 8913113 | *sctN* | type III secretion system ATPase SctN | 1.88 | 1.75E-03 |
| 8913180 | *EAMY_RS33525* | CoA-acylating methylmalonate-semialdehyde dehydrogenase | 1.74 | 1.90E-03 |
| 8912570 | *EAMY_RS19625* | SDR family oxidoreductase | -1.66 | 2.14E-03 |
| 8913093 | *hrpT* | HrpT family type III secretion system protein | 2.35 | 2.32E-03 |
| 8912639 | *cysN* | sulfate adenylyltransferase subunit CysN | 2.21 | 3.11E-03 |
| 8913660 | *proW* | glycine betaine/L-proline ABC transporter permease ProW | -2.91 | 3.28E-03 |
| 8913100 | *sctW* | type III secretion system gatekeeper subunit SctW | 1.79 | 4.22E-03 |
| 8912438 | *EAMY_RS33115* | aminodeoxychorismate synthase component II | 1.62 | 9.25E-03 |
| 8913095 | *sctD* | type III secretion system inner membrane ring subunit SctD | 1.56 | 1.09E-02 |
| 8911203 | *EAMY_RS17555* | reverse transcriptase family protein | -1.54 | 1.20E-02 |
| 8911998 | *EAMY_RS31245* | lysozyme inhibitor LprI family protein | -1.59 | 1.32E-02 |
| 8914606 | *cas1e* | type I-E CRISPR-associated endonuclease Cas1e | -1.80 | 1.48E-02 |
| 8914091 | *EAMY_RS27825* | hypothetical protein | -1.71 | 1.56E-02 |
| 8912016 | *EAMY_RS31370* | PepSY-associated TM helix domain-containing protein | -1.79 | 2.03E-02 |
| 8913106 | *EAMY_RS19975* | HPr kinase | 3.72 | 3.24E-02 |
| 23673361 | *EAMY_RS25875* | DapH/DapD/GlmU-related protein | -2.09 | 3.58E-02 |
| 8914161 | *EAMY_RS27745* | phosphatase PAP2 family protein | 2.11 | 3.68E-02 |
| 8914466 | *EAMY_RS25135* | heme ABC transporter ATP-binding protein | -1.58 | 3.69E-02 |
| 8914317 | *miaB* | tRNA (N6-isopentenyl adenosine(37)-C2)-methylthiotransferase MiaB | 1.68 | 3.92E-02 |

^a^ Gene IDs, locus tags, symbols and functions obtained from the DAVID database for *E. amylovora* CFBP 1430. MSTRG identifiers represent assembled and annotated by StringTie, which were not previously annotated in CFBP 1430 strain. They could be potentially new transcripts, alternative splice variants, or transcripts specific to the experimental conditions.

**Table S8**. DEGs in EaR2 with respect to Ea273 cells grown with 3 mM CuSO_4_ up to stationary phase^a^

| **Gene ID** | ***Symbol*** | **Gene function** | **logFC** | **FDR** |
| --- | --- | --- | --- | --- |
| 8912406 | *EAMY_RS27705* | polysaccharide biosynthesis tyrosine autokinase | 3.74 | 1.20E-16 |
| 8911759 | *EAMY_RS27275* | metal ABC transporter substrate-binding protein | -5.14 | 2.09E-16 |
| 8913950 | *sitC* | iron/manganese ABC transporter permease subunit SitC | -5.68 | 2.09E-16 |
| 8912413 | *EAMY_RS27715* | polysaccharide export protein | 3.39 | 2.09E-16 |
| 8912415 | *wcaK* | colanic acid biosynthesis pyruvyl transferase WcaK | 3.55 | 2.69E-16 |
| 8912479 | *spy* | ATP-independent periplasmic protein-refolding chaperone Spy | 5.23 | 2.50E-15 |
| 8914055 | *EAMY_RS34145* | OmpA family lipoprotein | 5.18 | 3.74E-15 |
| 8912414 | *EAMY_RS27680* | phage tailspike protein | 3.25 | 5.83E-15 |
| 8914463 | *ppsA* | phosphoenolpyruvate synthase | 3.94 | 6.76E-15 |
| 8911454 | *EAMY_RS22430* | OsmC family protein | -3.07 | 7.18E-15 |
| 8912926 | *EAMY_RS27665* | lipopolysaccharide biosynthesis protein | 3.09 | 2.21E-14 |
| 8913418 | *EAMY_RS24365* | glycine zipper 2TM domain-containing protein | 3.75 | 2.56E-14 |
| 8912357 | *EAMY_RS31520* | SulP family inorganic anion transporter | 3.09 | 1.11E-13 |
| 8913389 | *mraY* | phospho-N-acetylmuramoyl-pentapeptide-transferase | 1.93 | 1.45E-13 |
| 8912405 | *EAMY_RS27710* | protein-tyrosine-phosphatase | 3.58 | 1.48E-13 |
| 8912962 | *EAMY_RS23395* | DksA/TraR family C4-type zinc finger protein | -2.67 | 1.71E-13 |
| 8911411 | *EAMY_RS21710* | biofilm development regulator YmgB/AriR family protein | -2.25 | 2.47E-13 |
| 8913587 | *efeB* | iron uptake transporter deferrochelatase/peroxidase subunit | -2.72 | 2.52E-13 |
| 8912412 | *wbaP* | undecaprenyl-phosphate galactose phosphotransferase WbaP | 2.89 | 1.13E-12 |
| 8911811 | *kup* | low affinity potassium transporter Kup | -3.07 | 2.14E-12 |
| 8913392 | *ftsW* | cell division protein FtsW | 1.76 | 5.03E-12 |
| 8912059 | *hflC* | protease modulator HflC | 2.47 | 6.20E-12 |
| 8911877 | *denD* | D-erythronate dehydrogenase | 2.75 | 7.87E-12 |
| 8912568 | *EAMY_RS31450* | KpsF/GutQ family sugar-phosphate isomerase | 1.85 | 1.16E-11 |
| 8914378 | *efeU* | iron uptake transporter permease EfeU | -2.95 | 1.52E-11 |
| 8912410 | *EAMY_RS27685* | glycosyltransferase | 3.53 | 1.86E-11 |
| 8914413 | *EAMY_RS24975* | porin OmpC | -2.72 | 2.48E-11 |
| 8913134 | *EAMY_RS23320* | HutD family protein | -4.24 | 2.72E-11 |
| 8913135 | *hutI* | imidazolonepropionase | -3.44 | 2.72E-11 |
| 8912198 | *EAMY_RS33740* | SMP-30/gluconolactonase/LRE family protein | 2.43 | 2.73E-11 |
| 8911942 | *EAMY_RS30490* | omptin family outer membrane protease | -2.18 | 3.29E-11 |
| 8914129 | *sltY* | murein transglycosylase | 2.11 | 3.29E-11 |
| 8911821 | *pta* | phosphate acetyltransferase | 2.19 | 3.89E-11 |
| 8914269 | *EAMY_RS21760* | IclR family transcriptional regulator | -2.32 | 4.20E-11 |
| 8914406 | *emtA* | membrane-bound lytic murein transglycosylase EmtA | 3.63 | 4.20E-11 |
| 8913391 | *murE* | UDP-N-acetylmuramoyl-L-alanyl-D-glutamate--2,6-diaminopimelate ligase | 1.62 | 4.20E-11 |
| 8911284 | *EAMY_RS19705* | disulfide bond formation protein B | 3.66 | 4.29E-11 |
| 41697647 | *EAMY_RS36095* | hypothetical protein | 2.17 | 4.29E-11 |
| 8914930 | *mlaD* | outer membrane lipid asymmetry maintenance protein MlaD | 2.20 | 7.53E-11 |
| 8911807 | *ompC* | porin OmpC | 4.19 | 7.53E-11 |
| 8912164 | *glpD* | glycerol-3-phosphate dehydrogenase | -2.59 | 7.76E-11 |
| 8914716 | *EAMY_RS33110* | aspartate aminotransferase family protein | 2.31 | 8.21E-11 |
| 8914929 | *mlaC* | phospholipid-binding protein MlaC | 2.45 | 8.56E-11 |
| 8913388 | *murG* | undecaprenyldiphospho-muramoylpentapeptide beta-N-acetylglucosaminyltransferase | 1.60 | 1.20E-10 |
| 8914804 | *EAMY_RS32695* | YjbH domain-containing protein | 1.96 | 1.21E-10 |
| 8914111 | *EAMY_RS26430* | UDP-glucose/GDP-mannose dehydrogenase family protein | 2.01 | 1.24E-10 |
| 8911849 | *EAMY_RS28685* | hypothetical protein | 3.68 | 1.47E-10 |
| 8914851 | *EAMY_RS23485* | serine hydrolase | 2.16 | 1.50E-10 |
| 8913458 | *nuoM* | NADH-quinone oxidoreductase subunit M | 1.58 | 1.73E-10 |
| 8913256 | *skp* | molecular chaperone Skp | 2.21 | 1.73E-10 |
| 8912407 | *EAMY_RS27700* | glycosyltransferase family 2 protein | 3.69 | 1.84E-10 |
| 8914837 | *EAMY_RS19810* | carbon starvation CstA family protein | 1.84 | 1.89E-10 |
| 8913909 | *EAMY_RS27540* | APC family permease | 2.53 | 2.07E-10 |
| 8912711 | *EAMY_RS30930* | acyltransferase | 3.50 | 2.07E-10 |
| 8913315 | *EAMY_RS23920* | DUF2057 family protein | -2.22 | 2.14E-10 |
| 8914339 | *dps* | DNA starvation/stationary phase protection protein Dps | -2.20 | 2.61E-10 |
| 8911855 | *EAMY_RS28735* | Nramp family divalent metal transporter | -2.32 | 2.72E-10 |
| 8912409 | *EAMY_RS27690* | glycosyltransferase family 4 protein | 3.75 | 3.64E-10 |
| 8913507 | *EAMY_RS22435* | RcnB family protein | -2.65 | 4.88E-10 |
| 8914226 | *EAMY_RS29215* | M4 family metallopeptidase | -2.51 | 4.88E-10 |
| 8914661 | *EAMY_RS19450* | glutathionylspermidine synthase family protein | 2.57 | 5.48E-10 |
| 8914376 | *efeO* | iron uptake system protein EfeO | -2.75 | 5.74E-10 |
| 8911532 | *EAMY_RS24050* | MarR family transcriptional regulator | -1.79 | 6.11E-10 |
| 8911738 | *EAMY_RS27055* | flippase | 2.87 | 6.32E-10 |
| 8913318 | *EAMY_RS29135* | MFS transporter | 3.42 | 7.20E-10 |
| 8913046 | *hflK* | FtsH protease activity modulator HflK | 2.29 | 7.74E-10 |
| 8914581 | *murQ* | N-acetylmuramic acid 6-phosphate etherase | 2.06 | 8.54E-10 |
| 8912719 | *dppA* | dipeptide ABC transporter periplasmic-binding protein DppA | -2.94 | 1.17E-09 |
| 8911760 | *EAMY_RS27305* | protein kinase family protein | -3.01 | 1.39E-09 |
| 8911735 | *flhC* | flagellar transcriptional regulator FlhC | -2.16 | 1.43E-09 |
| 69102764 | *EAMY_RS36505* | DUF5993 family protein | 4.61 | 1.97E-09 |
| 8913053 | *hutU* | urocanate hydratase | -3.33 | 2.00E-09 |
| 8912211 | *EAMY_RS33860* | AsmA family protein | -1.64 | 2.57E-09 |
| 8912775 | *fabD* | ACP S-malonyltransferase | 1.50 | 3.30E-09 |
| 8914619 | *cas7e* | type I-E CRISPR-associated protein Cas7/Cse4/CasC | 2.31 | 3.84E-09 |
| 8911267 | *EAMY_RS19515* | aminotransferase class III-fold pyridoxal phosphate-dependent enzyme | 1.72 | 4.21E-09 |
| 8914174 | *ispC* | 1-deoxy-D-xylulose-5-phosphate reductoisomerase | 2.19 | 4.38E-09 |
| 30316917 | *EAMY_RS35010* | glycine zipper 2TM domain-containing protein | 3.09 | 4.64E-09 |
| 8913479 | *EAMY_RS26275* | YciC family protein | 2.43 | 4.93E-09 |
| 8911262 | *ribB* | 3,4-dihydroxy-2-butanone-4-phosphate synthase | -4.11 | 5.12E-09 |
| 8914075 | *hns* | histone-like nucleoid-structuring protein H-NS | -1.85 | 5.48E-09 |
| 8911605 | *guaD* | guanine deaminase | 1.59 | 6.78E-09 |
| 8914346 | *cspD* | cold shock-like protein CspD | -2.65 | 7.42E-09 |
| 8914909 | *parE* | DNA topoisomerase IV subunit B | 2.94 | 8.12E-09 |
| 8911485 | *EAMY_RS23200* | Yip1 family protein | -2.92 | 8.22E-09 |
| 8912604 | *EAMY_RS33105* | YccS/YhfK family putative transporter | 1.95 | 9.98E-09 |
| 8914018 | *srlD* | sorbitol-6-phosphate dehydrogenase | 3.43 | 1.09E-08 |
| 8911758 | *mtfA* | DgsA anti-repressor MtfA | -1.59 | 1.29E-08 |
| 8913422 | *hchA* | glyoxalase III HchA | -1.51 | 1.40E-08 |
| 8911214 | *glpK* | glycerol kinase GlpK | -2.03 | 1.43E-08 |
| 8914275 | *secD* | protein translocase subunit SecD | 1.87 | 1.64E-08 |
| 8911850 | *EAMY_RS28690* | hypothetical protein | -1.56 | 2.29E-08 |
| 8911608 | *EAMY_RS25545* | DUF441 domain-containing protein | -1.61 | 2.85E-08 |
| 8913151 | *ilvD* | dihydroxy-acid dehydratase | 1.54 | 3.22E-08 |
| 8914640 | *dcp* | peptidyl-dipeptidase Dcp | 1.74 | 3.26E-08 |
| 8913261 | *EAMY_RS34315* | phage holin, lambda family | 3.45 | 3.51E-08 |
| 8913691 | *ptsI* | phosphoenolpyruvate-protein phosphotransferase PtsI | 2.30 | 3.77E-08 |
| 8912408 | *EAMY_RS27695* | EpsG family protein | 2.96 | 3.96E-08 |
| 8913439 | *proV* | glycine betaine/L-proline ABC transporter ATP-binding protein ProV | -3.59 | 4.09E-08 |
| 8914874 | *EAMY_RS25055* | fructosamine kinase family protein | -2.00 | 4.75E-08 |
| 8912683 | *truA* | tRNA pseudouridine(38-40) synthase TruA | 1.57 | 5.13E-08 |
| 8914743 | *EAMY_RS33965* | organic hydroperoxide resistance protein | 1.71 | 5.32E-08 |
| 8911808 | *rcsD* | phosphotransferase RcsD | 1.83 | 5.61E-08 |
| 8914714 | *crp* | cAMP-activated global transcriptional regulator CRP | 1.61 | 5.61E-08 |
| 8914320 | *seqA* | replication initiation negative regulator SeqA | 1.54 | 5.84E-08 |
| 8914614 | *cas6e* | type I-E CRISPR-associated protein Cas6/Cse3/CasE | 2.31 | 6.58E-08 |
| 8912974 | *EAMY_RS17890* | MIP/aquaporin family protein | -2.58 | 6.67E-08 |
| 8912837 | *cheZ* | protein phosphatase CheZ | 1.95 | 6.67E-08 |
| 8912464 | *EAMY_RS19750* | cytosine permease | 1.52 | 7.98E-08 |
| 8914955 | *EAMY_RS32440* | Na+/H+ antiporter | 1.58 | 8.21E-08 |
| 8914077 | *ybbA* | putative ABC transporter ATP-binding protein YbbA | 2.06 | 8.40E-08 |
| 8914708 | *fkpA* | FKBP-type peptidyl-prolyl cis-trans isomerase | 1.98 | 8.40E-08 |
| 8914015 | *EAMY_RS31475* | PTS glucitol/sorbitol transporter subunit IIB | 2.65 | 8.44E-08 |
| 8914540 | *EAMY_RS28020* | YejG family protein | -2.04 | 9.14E-08 |
| 8912482 | *atpD* | F0F1 ATP synthase subunit beta | 1.80 | 9.40E-08 |
| 8913927 | *secF* | protein translocase subunit SecF | 1.86 | 9.55E-08 |
| 8912523 | *proY* | proline-specific permease ProY | 1.56 | 1.15E-07 |
| 8911193 | *EAMY_RS17320* | hypothetical protein | 1.88 | 1.25E-07 |
| 8912365 | *fabF* | beta-ketoacyl-ACP synthase II | 1.95 | 1.27E-07 |
| 8912563 | *EAMY_RS26950* | chemotaxis response regulator protein-glutamate methylesterase | 1.54 | 1.36E-07 |
| 8914121 | *rplS* | 50S ribosomal protein L19 | 2.81 | 1.41E-07 |
| 8912990 | *EAMY_RS34040* | NAD(P)-dependent alcohol dehydrogenase | 1.84 | 1.64E-07 |
| 8914146 | *EAMY_RS25480* | DUF1460 domain-containing protein | 2.24 | 1.68E-07 |
| 8913139 | *hutH* | histidine ammonia-lyase | -2.74 | 1.69E-07 |
| 8913934 | *pgm* | phosphoglucomutase (alpha-D-glucose-1,6-bisphosphate-dependent) | 1.59 | 1.70E-07 |
| 41697605 | *EAMY_RS35880* | pseudo | -1.70 | 1.76E-07 |
| 8913687 | *ychH* | stress-induced protein YchH | -1.95 | 1.88E-07 |
| 8913943 | *EAMY_RS21120* | hypothetical protein | 1.62 | 2.03E-07 |
| 8914058 | *EAMY_RS25635* | carbonic anhydrase | -1.74 | 2.18E-07 |
| 8914408 | *kdsA* | 3-deoxy-8-phosphooctulonate synthase | 1.75 | 2.63E-07 |
| 8914008 | *EAMY_RS20230* | sugar porter family MFS transporter | 1.63 | 2.72E-07 |
| 8911614 | *EAMY_RS25570* | TonB-dependent receptor | -1.81 | 2.77E-07 |
| 8913162 | *EAMY_RS33810* | serralysin family metalloprotease | -1.56 | 3.01E-07 |
| 8914016 | *EAMY_RS31470* | glucitol/sorbitol-specific PTS transporter subunit IIA | 2.61 | 3.19E-07 |
| 8913836 | *rpsC* | 30S ribosomal protein S3 | 1.79 | 3.27E-07 |
| 8911266 | *EAMY_RS19510* | nucleoside 2-deoxyribosyltransferase | 1.78 | 3.61E-07 |
| 8913630 | *EAMY_RS31515* | dipeptidase | 1.71 | 4.19E-07 |
| 8914347 | *EAMY_RS26745* | 2OG-Fe dioxygenase family protein | -2.22 | 5.08E-07 |
| 8912909 | *ftsH* | ATP-dependent zinc metalloprotease FtsH | 2.15 | 5.72E-07 |
| 8912765 | *ppnN* | nucleotide 5'-monophosphate nucleosidase PpnN | 1.83 | 5.84E-07 |
| 8914574 | *ndk* | nucleoside-diphosphate kinase | 1.50 | 6.60E-07 |
| 8914931 | *mlaE* | lipid asymmetry maintenance ABC transporter permease subunit MlaE | 1.55 | 6.88E-07 |
| 8912245 | *EAMY_RS34340* | F0F1 ATP synthase subunit epsilon | 1.78 | 6.89E-07 |
|  | MSTRG.2581.1 | Unannotated transcript | 1.76 | 7.64E-07 |
| 8914405 | *wbaP* | undecaprenyl-phosphate galactose phosphotransferase WbaP | 1.66 | 7.73E-07 |
| 8914591 | *trxC* | thioredoxin TrxC | -1.90 | 7.99E-07 |
| 8914109 | *EAMY_RS26295* | YciI family protein | -1.85 | 8.10E-07 |
| 8914242 | *hpt* | hypoxanthine phosphoribosyltransferase | -2.40 | 8.14E-07 |
| 30316876 | *EAMY_RS34805* | hypothetical protein | -2.52 | 8.29E-07 |
| 8913311 | *mglB* | galactose/glucose ABC transporter substrate-binding protein MglB | 1.67 | 8.40E-07 |
| 43837317 | *EAMY_RS36240* | hypothetical protein | 3.31 | 9.39E-07 |
| 8912370 | *EAMY_RS32410* | DUF485 domain-containing protein | -1.59 | 9.57E-07 |
| 8912749 | *emrB* | multidrug efflux MFS transporter permease subunit EmrB | 1.99 | 9.85E-07 |
| 8911625 | *EAMY_RS25630* | methyl-accepting chemotaxis protein | -2.10 | 1.02E-06 |
| 8911885 | *EAMY_RS29190* | pseudo | -3.11 | 1.05E-06 |
| 8911466 | *EAMY_RS22550* | hypothetical protein | -1.76 | 1.05E-06 |
| 8911830 | *EAMY_RS28455* | DedA family protein | 1.92 | 1.05E-06 |
| 8912483 | *atpG* | F0F1 ATP synthase subunit gamma | 1.84 | 1.05E-06 |
| 41697620 | *EAMY_RS35955* | pseudo | -1.91 | 1.17E-06 |
| 8914869 | *EAMY_RS25515* | DUF1283 family protein | 2.11 | 1.19E-06 |
| 8914598 | *trmD* | tRNA (guanosine(37)-N1)-methyltransferase TrmD | 2.02 | 1.25E-06 |
| 8912630 | *EAMY_RS25640* | DUF4385 domain-containing protein | -1.85 | 1.29E-06 |
| 8913671 | *lpxO* | lipid A hydroxylase LpxO | -1.87 | 1.32E-06 |
| 8914758 | *EAMY_RS17540* | response regulator transcription factor | -2.51 | 1.37E-06 |
| 8912695 | *EAMY_RS32250* | lysine N(6)-hydroxylase/L-ornithine N(5)-oxygenase family protein | -2.05 | 1.42E-06 |
| 8914250 | *degP* | serine endoprotease DegP | 1.73 | 1.51E-06 |
| 8914416 | *EAMY_RS26280* | septation protein A | 2.13 | 1.60E-06 |
| 8911964 | *dnaJ* | molecular chaperone DnaJ | 2.56 | 2.08E-06 |
| 23673323 | *EAMY_RS20225* | SprT family zinc-dependent metalloprotease | -2.13 | 2.20E-06 |
| 8913163 | *EAMY_RS22475* | hypothetical protein | -2.66 | 2.29E-06 |
| 8911909 | *EAMY_RS29705* | winged helix-turn-helix domain-containing protein | -1.85 | 2.36E-06 |
| 8911707 | *htpX* | protease HtpX | 1.72 | 2.51E-06 |
| 8911401 | *EAMY_RS21635* | hypothetical protein | -2.37 | 2.65E-06 |
| 8911225 | *EAMY_RS18545* | fimbrial protein | 1.79 | 2.78E-06 |
| 8913883 | *rpmC* | 50S ribosomal protein L29 | 1.97 | 2.88E-06 |
| 8912525 | *EAMY_RS25120* | NlpC/P60 family protein | -2.02 | 3.21E-06 |
| 8912480 | *atpH* | F0F1 ATP synthase subunit delta | 1.78 | 3.26E-06 |
| 8914945 | *rplI* | 50S ribosomal protein L9 | 1.52 | 3.55E-06 |
| 8912069 | *EAMY_RS32010* | asparagine synthase-related protein | 1.88 | 3.67E-06 |
| 30316924 | *EAMY_RS35045* | YmiA family putative membrane protein | -1.92 | 4.03E-06 |
| 8913824 | *rplW* | 50S ribosomal protein L23 | 1.86 | 4.06E-06 |
| 8912601 | *EAMY_RS21630* | hypothetical protein | -1.91 | 4.16E-06 |
| 8914504 | *EAMY_RS27250* | hypothetical protein | -1.50 | 4.21E-06 |
| 8914528 | *EAMY_RS19805* | YbdD/YjiX family protein | 1.78 | 4.22E-06 |
| 8912565 | *cheY* | chemotaxis response regulator CheY | 2.62 | 4.25E-06 |
| 8913842 | *rpsS* | 30S ribosomal protein S19 | 1.67 | 4.64E-06 |
| 8913040 | *EAMY_RS24890* | SirB2 family protein | 1.65 | 5.03E-06 |
| 8913529 | *ppiA* | peptidylprolyl isomerase A | 1.80 | 5.17E-06 |
| 77383444 | *EAMY_RS36770* | hypothetical protein | -2.13 | 5.27E-06 |
| 8911770 | *EAMY_RS27385* | LysE family translocator | -2.24 | 5.43E-06 |
| 8911706 | *EAMY_RS26685* | YobH family protein | 1.79 | 6.44E-06 |
| 8913826 | *rplC* | 50S ribosomal protein L3 | 2.02 | 6.81E-06 |
| 8913639 | *EAMY_RS21045* | helix-turn-helix domain-containing protein | 2.04 | 8.67E-06 |
| 8913753 | *EAMY_RS20730* | DUF1440 domain-containing protein | 1.60 | 8.83E-06 |
| 8914442 | *EAMY_RS19660* | YlaC family protein | 1.69 | 8.98E-06 |
| 8911400 | *crcB* | fluoride efflux transporter CrcB | -1.61 | 9.80E-06 |
| 8912218 | *EAMY_RS33980* | hypothetical protein | 3.01 | 1.00E-05 |
| 88183296 | *EAMY_RS36865* | pseudo | -2.41 | 1.05E-05 |
| 8912771 | *mnmC* | bifunctional tRNA (5-methylaminomethyl-2-thiouridine)(34)-methyltransferase MnmD/FAD-dependent 5-carboxymethylaminomethyl-2-thiouridine(34) oxidoreductase MnmC | -1.76 | 1.11E-05 |
| 8914583 | *tadA* | tRNA adenosine(34) deaminase TadA | 1.72 | 1.14E-05 |
| 8913210 | *EAMY_RS24905* | META domain-containing protein | 1.53 | 1.14E-05 |
|  | MSTRG.2609.1 | Unannotated transcript | -3.38 | 1.18E-05 |
| 8911332 | *EAMY_RS20490* | trypsin-like peptidase domain-containing protein | 1.70 | 1.36E-05 |
| 8893791 | *EAMY_RS34520* | recombinase family protein | -1.81 | 1.40E-05 |
| 23673438 | *EAMY_RS33805* | protease inhibitor Inh/omp19 family protein | -1.73 | 1.43E-05 |
| 8893782 | *EAMY_RS34430* | H-NS family nucleoid-associated regulatory protein | 1.90 | 1.46E-05 |
| 8914689 | *yhcN* | peroxide/acid stress response protein YhcN | 3.41 | 1.50E-05 |
| 55585574 | *EAMY_RS36430* | hypothetical protein | -2.51 | 1.54E-05 |
| 8914822 | *groL* | chaperonin GroEL | 2.90 | 1.57E-05 |
| 8912570 | *EAMY_RS19625* | SDR family oxidoreductase | -2.26 | 1.57E-05 |
| 8911595 | *EAMY_RS25320* | DUF1289 domain-containing protein | -1.67 | 1.70E-05 |
| 8911459 | *EAMY_RS22470* | leucine-rich repeat domain-containing protein | 1.81 | 1.84E-05 |
| 8911600 | *araA* | L-arabinose isomerase | -2.80 | 1.88E-05 |
| 8913047 | *hflX* | ribosome rescue GTPase HflX | 1.52 | 1.97E-05 |
| 23673368 | *EAMY_RS27210* | DUF6388 family protein | -1.54 | 2.07E-05 |
| 8914432 | *EAMY_RS25810* | DUF2946 domain-containing protein | -2.23 | 2.14E-05 |
| 8912107 | *EAMY_RS32245* | aspartate aminotransferase family protein | -2.39 | 2.16E-05 |
| 8913783 | *cbl* | HTH-type transcriptional regulator Cbl | -3.24 | 2.30E-05 |
| 8913157 | *ilvC* | ketol-acid reductoisomerase | 1.95 | 2.36E-05 |
| 8914697 | *zntR* | Zn(2+)-responsive transcriptional regulator | -1.54 | 2.47E-05 |
| 8914635 | *EAMY_RS24700* | hypothetical protein | -1.86 | 2.50E-05 |
| 8913885 | *rplB* | 50S ribosomal protein L2 | 1.53 | 2.50E-05 |
| 8914043 | *dkgA* | 2,5-didehydrogluconate reductase DkgA | -2.04 | 2.56E-05 |
| 8913832 | *rpoB* | DNA-directed RNA polymerase subunit beta | 1.51 | 2.63E-05 |
| 8912587 | *EAMY_RS24520* | benzoate/H(+) symporter BenE family transporter | -2.05 | 2.63E-05 |
| 8913952 | *EAMY_RS27295* | tRNA-Asn | -3.95 | 2.63E-05 |
| 8914843 | *trpR* | trp operon repressor | 1.66 | 2.76E-05 |
| 8912522 | *tig* | trigger factor | 3.22 | 3.00E-05 |
| 23673330 | *EAMY_RS20630* | hypothetical protein | -2.75 | 3.01E-05 |
| 8911328 | *EAMY_RS20430* | hemolysin III family protein | -1.65 | 3.07E-05 |
| 8911658 | *asr* | acid resistance repetitive basic protein Asr | 1.91 | 3.60E-05 |
| 8914890 | *EAMY_RS34170* | hypothetical protein | -1.51 | 4.08E-05 |
| 8913829 | *rpsR* | 30S ribosomal protein S18 | 1.53 | 4.17E-05 |
| 8911904 | *ung* | uracil-DNA glycosylase | 2.74 | 4.22E-05 |
| 8914105 | *pal* | peptidoglycan-associated lipoprotein Pal | 1.66 | 4.49E-05 |
| 8914084 | *EAMY_RS18575* | Rsd/AlgQ family anti-sigma factor | -1.97 | 4.49E-05 |
| 8911558 | *EAMY_RS24600* | hypothetical protein | -2.25 | 4.55E-05 |
| 8911851 | *EAMY_RS28695* | universal stress protein | -1.56 | 4.93E-05 |
| 8913471 | *EAMY_RS28135* | hypothetical protein | 1.56 | 5.00E-05 |
| 8911852 | *EAMY_RS28705* | type III effector | 1.94 | 5.07E-05 |
| 8912486 | *atpA* | F0F1 ATP synthase subunit alpha | 1.56 | 5.60E-05 |
| 8914216 | *wzxE* | lipid III flippase WzxE | 1.59 | 5.62E-05 |
| 8912923 | *EAMY_RS23970* | YccJ family protein | -3.15 | 6.20E-05 |
| 8913869 | *rplV* | 50S ribosomal protein L22 | 1.66 | 6.61E-05 |
| 8912207 | *mntP* | manganese efflux pump MntP | 2.08 | 6.78E-05 |
| 8911569 | *ycgZ* | regulatory protein YcgZ | -2.15 | 6.85E-05 |
| 8914621 | *casA* | type I-E CRISPR-associated protein Cse1/CasA | 1.60 | 6.92E-05 |
| 8911692 | *tdk* | thymidine kinase | -1.99 | 7.33E-05 |
| 8914211 | *EAMY_RS24380* | mannosyl-3-phosphoglycerate phosphatase-related protein | -1.83 | 7.33E-05 |
| 8912438 | *EAMY_RS33115* | aminodeoxychorismate synthase component II | 2.22 | 8.13E-05 |
| 8914629 | *EAMY_RS24695* | YgdI/YgdR family lipoprotein | -1.80 | 8.85E-05 |
| 8913487 | *EAMY_RS27345* | hypothetical protein | -1.67 | 9.00E-05 |
| 8914178 | *rsmJ* | 16S rRNA (guanine(1516)-N(2))-methyltransferase RsmJ | 1.63 | 9.22E-05 |
| 8912820 | *EAMY_RS24215* | flagellar basal body L-ring protein FlgH | 2.36 | 1.01E-04 |
| 8913068 | *EAMY_RS32040* | class I SAM-dependent methyltransferase | 2.24 | 1.02E-04 |
| 8914818 | *EAMY_RS24690* | YgdI/YgdR family lipoprotein | -1.86 | 1.02E-04 |
| 8914606 | *cas1e* | type I-E CRISPR-associated endonuclease Cas1e | 2.66 | 1.02E-04 |
| 8914698 | *rplQ* | 50S ribosomal protein L17 | 1.82 | 1.02E-04 |
|  | MSTRG.1959.1 | Unannotated transcript | -1.66 | 1.02E-04 |
| 8913632 | *pqqC* | pyrroloquinoline-quinone synthase PqqC | 1.88 | 1.04E-04 |
| 8911418 | *EAMY_RS21805* | cell envelope integrity TolA C-terminal domain-containing protein | -1.84 | 1.04E-04 |
| 77383446 | *EAMY_RS36780* | hypothetical protein | -1.64 | 1.05E-04 |
| 8913534 | *cpoB* | cell division protein CpoB | 1.77 | 1.10E-04 |
| 30316846 | *EAMY_RS34655* | GIY-YIG nuclease family protein | -2.01 | 1.17E-04 |
| 8911358 | *cysG* | siroheme synthase CysG | -2.33 | 1.21E-04 |
| 8914660 | *EAMY_RS19455* | DUF1190 family protein | 1.92 | 1.25E-04 |
| 8914513 | *EAMY_RS27260* | tRNA-Asn | -2.11 | 1.31E-04 |
| 8911824 | *EAMY_RS28370* | 4-aminobutyrate--2-oxoglutarate transaminase | 1.53 | 1.41E-04 |
| 8911888 | *sseA* | 3-mercaptopyruvate sulfurtransferase | 1.78 | 1.41E-04 |
| 8912463 | *artJ* | arginine ABC transporter substrate-binding protein | 1.56 | 1.46E-04 |
| 8912391 | *budA* | acetolactate decarboxylase | 1.85 | 1.51E-04 |
| 8914792 | *fpr* | ferredoxin--NADP(+) reductase | 1.99 | 1.53E-04 |
| 8913092 | *EAMY_RS30310* | cystathionine gamma-synthase family protein | -1.89 | 1.56E-04 |
| 8913760 | *htpG* | molecular chaperone HtpG | 2.51 | 1.75E-04 |
| 8912652 | *EAMY_RS28880* | sulfate ABC transporter substrate-binding protein | -2.05 | 1.90E-04 |
| 8913834 | *rpsQ* | 30S ribosomal protein S17 | 1.82 | 1.95E-04 |
| 8914447 | *rseC* | SoxR-reducing system protein RseC | 1.50 | 2.02E-04 |
| 8913341 | *EAMY_RS17410* | pseudo | -1.84 | 2.03E-04 |
| 8911441 | *EAMY_RS22335* | colicin-like pore-forming protein | 1.95 | 2.05E-04 |
| 8912761 | *rnc* | ribonuclease III | -2.15 | 2.13E-04 |
| 8913429 | *EAMY_RS19345* | helix-turn-helix transcriptional regulator | -2.53 | 2.15E-04 |
| 8913888 | *hslU* | HslU--HslV peptidase ATPase subunit | 2.41 | 2.24E-04 |
| 8913010 | *EAMY_RS31960* | co-chaperone GroES | 2.37 | 2.28E-04 |
| 8914722 | *EAMY_RS33475* | zinc/cadmium/mercury/lead-transporting ATPase | -1.52 | 2.43E-04 |
| 8914764 | *yidC* | membrane protein insertase YidC | 2.01 | 2.52E-04 |
| 8911749 | *EAMY_RS27145* | acyltransferase family protein | 1.86 | 2.74E-04 |
| 8913576 | *dxs* | 1-deoxy-D-xylulose-5-phosphate synthase | 1.87 | 2.74E-04 |
| 8913659 | *EAMY_RS30005* | hypothetical protein | -2.18 | 2.75E-04 |
| 8912850 | *EAMY_RS29700* | flagellin | -1.59 | 3.00E-04 |
| 8914068 | *EAMY_RS33075* | LysE family translocator | -1.55 | 3.27E-04 |
| 8912667 | *EAMY_RS30145* | DUF3461 family protein | -2.04 | 3.30E-04 |
| 8913854 | *rnpA* | ribonuclease P protein component | 1.53 | 3.37E-04 |
| 8914643 | *EAMY_RS20320* | UDP-3-O-(3-hydroxymyristoyl)glucosamine N-acyltransferase | -2.00 | 3.51E-04 |
| 8913299 | *exbB* | tol-pal system-associated acyl-CoA thioesterase | 1.94 | 4.01E-04 |
| 8912555 | *motB* | flagellar motor protein MotB | -1.63 | 4.01E-04 |
| 23673361 | *EAMY_RS25875* | DapH/DapD/GlmU-related protein | -3.02 | 4.06E-04 |
| 69102788 | *EAMY_RS36625* | pseudo | -1.57 | 4.15E-04 |
| 8914426 | *EAMY_RS26105* | LysR family transcriptional regulator | -1.80 | 4.49E-04 |
| 8913835 | *rpmD* | 50S ribosomal protein L30 | 3.55 | 4.53E-04 |
| 23673414 | *EAMY_RS32005* | cupin domain-containing protein | 1.74 | 4.90E-04 |
| 8913805 | *lepB* | signal peptidase I | 1.97 | 5.37E-04 |
| 8914120 | *murI* | glutamate racemase | 2.40 | 5.39E-04 |
| 8913093 | *hrpT* | HrpT family type III secretion system protein | 2.53 | 5.39E-04 |
| 8912237 | *EAMY_RS34165* | protein-tyrosine phosphatase family protein | -1.57 | 5.39E-04 |
| 8911546 | *rcsA* | transcriptional regulator RcsA | 1.51 | 5.50E-04 |
| 8912478 | *astB* | N-succinylarginine dihydrolase | -2.88 | 5.58E-04 |
| 8913801 | *fabA* | bifunctional 3-hydroxydecanoyl-ACP dehydratase/trans-2-decenoyl-ACP isomerase | 1.56 | 6.00E-04 |
| 8912616 | *EAMY_RS19650* | spore coat protein U domain-containing protein | -2.41 | 6.16E-04 |
| 8914674 | *nusA* | transcription termination factor NusA | 1.82 | 6.30E-04 |
| 8914093 | *EAMY_RS30800* | DedA family protein | -1.54 | 6.44E-04 |
| 8913734 | *dtd* | D-aminoacyl-tRNA deacylase | 4.36 | 6.59E-04 |
| 8911549 | *EAMY_RS24480* | antiterminator Q family protein | -1.90 | 6.76E-04 |
| 8911476 | *EAMY_RS23010* | RHS repeat-associated core domain-containing protein | 2.42 | 6.95E-04 |
| 8913660 | *proW* | glycine betaine/L-proline ABC transporter permease ProW | -3.27 | 7.64E-04 |
| 43837323 | *EAMY_RS36270* | pseudo | -1.62 | 9.07E-04 |
| 8912870 | *fliM* | flagellar motor switch protein FliM | -2.55 | 9.93E-04 |
| 8912192 | *EAMY_RS33690* | ankyrin repeat domain-containing protein | 2.21 | 1.06E-03 |
| 8912361 | *EAMY_RS26190* | hypothetical protein | -1.51 | 1.12E-03 |
| 8911933 | *cas2e* | type I-E CRISPR-associated endoribonuclease Cas2e | 4.93 | 1.20E-03 |
| 30316899 | *rmf* | ribosome modulation factor | -1.57 | 1.36E-03 |
| 69102796 | *EAMY_RS36665* | pseudo | -2.41 | 1.43E-03 |
| 8914172 | *grpE* | nucleotide exchange factor GrpE | 2.75 | 1.50E-03 |
| 8913802 | *rffC* | dTDP-4-amino-4,6-dideoxy-D-galactose acyltransferase | 2.72 | 1.54E-03 |
| 8913450 | *nuoE* | NADH-quinone oxidoreductase subunit NuoE | -4.43 | 1.63E-03 |
| 30316911 | *EAMY_RS34980* | hypothetical protein | -1.61 | 1.79E-03 |
| 8914065 | *tatC* | Sec-independent protein translocase subunit TatC | 1.64 | 1.83E-03 |
| 8911403 | *EAMY_RS21645* | hypothetical protein | -2.43 | 1.98E-03 |
| 8913269 | *EAMY_RS33720* | siderophore-interacting protein | -2.12 | 2.07E-03 |
| 8913387 | *birA* | bifunctional biotin--[acetyl-CoA-carboxylase] ligase/biotin operon repressor BirA | 4.07 | 2.18E-03 |
| 8913067 | *EAMY_RS32030* | 2-dehydropantoate 2-reductase N-terminal domain-containing protein | 1.52 | 2.19E-03 |
| 8914830 | *EAMY_RS33555* | LuxR C-terminal-related transcriptional regulator | -2.03 | 2.19E-03 |
| 8912858 | *fliG* | flagellar motor switch protein FliG | -3.38 | 2.35E-03 |
| 55585579 | *EAMY_RS36455* | transcriptional regulator | -1.53 | 2.42E-03 |
| 43500274 | *EAMY_RS36175* | hypothetical protein | -2.06 | 2.61E-03 |
| 8911556 | *EAMY_RS24590* | putative holin | -1.56 | 2.70E-03 |
| 8913810 | *rsxB* | electron transport complex subunit RsxB | -2.94 | 2.90E-03 |
|  | MSTRG.28.1 | Unannotated transcript | -1.69 | 2.93E-03 |
| 8911634 | *EAMY_RS25705* | helix-turn-helix domain-containing protein | -1.59 | 3.11E-03 |
| 8913133 | *ppiD* | peptidylprolyl isomerase | 2.24 | 3.42E-03 |
| 8914091 | *EAMY_RS27825* | hypothetical protein | -1.77 | 3.49E-03 |
| 8912580 | *lon* | endopeptidase La | 1.58 | 3.51E-03 |
| 8914133 | *rho* | transcription termination factor Rho | 2.08 | 3.57E-03 |
| 23673313 | *dndE* | DNA sulfur modification protein DndE | 1.59 | 3.59E-03 |
| 8912307 | *asnB* | asparagine synthase B | 1.92 | 4.22E-03 |
| 8911655 | *EAMY_RS25880* | hypothetical protein | -2.29 | 4.24E-03 |
| 8911525 | *hspQ* | heat shock protein HspQ | 1.93 | 4.29E-03 |
| 8912768 | *EAMY_RS24005* | GlpM family protein | -1.76 | 4.32E-03 |
| 8914070 | *tauB* | taurine ABC transporter ATP-binding subunit | -3.07 | 4.58E-03 |
| 30316988 | *pqqA* | pyrroloquinoline quinone precursor peptide PqqA | 2.48 | 4.90E-03 |
| 8914553 | *EAMY_RS28330* | ADP-ribosyltransferase | -1.55 | 4.99E-03 |
| 8913144 | *EAMY_RS24575* | LPS 1,2-N-acetylglucosaminetransferase | -1.82 | 5.02E-03 |
| 8911880 | *EAMY_RS29155* | RluA family pseudouridine synthase | -1.79 | 5.07E-03 |
| 8912016 | *EAMY_RS31370* | PepSY-associated TM helix domain-containing protein | -1.70 | 5.21E-03 |
| 8912009 | *tssK* | type VI secretion system baseplate subunit TssK | -2.98 | 5.26E-03 |
| 8913570 | *EAMY_RS17820* | sulfate ABC transporter substrate-binding protein | -2.06 | 5.31E-03 |
|  | MSTRG.1813.1 | Unannotated transcript | -2.10 | 5.54E-03 |
| 8913908 | *EAMY_RS26095* | hypothetical protein | -2.21 | 5.81E-03 |
| 8911543 | *EAMY_RS24350* | PrpF domain-containing protein | 1.53 | 6.37E-03 |
| 8911351 | *xni* | flap endonuclease Xni | 1.66 | 6.52E-03 |
| 8913849 | *rpsE* | 30S ribosomal protein S5 | 3.85 | 6.52E-03 |
| 8912007 | *tssM* | type VI secretion system membrane subunit TssM | -2.68 | 6.73E-03 |
| 8913746 | *EAMY_RS28655* | tRNA-Arg | -3.13 | 7.51E-03 |
| 8914215 | *rffA* | dTDP-4-amino-4,6-dideoxygalactose transaminase | 1.57 | 7.93E-03 |
| 8913464 | *EAMY_RS33585* | helix-turn-helix transcriptional regulator | -1.88 | 9.11E-03 |
| 8914618 | *cas5e* | type I-E CRISPR-associated protein Cas5/CasD | 3.50 | 9.27E-03 |
| 8914061 | *bsmA* | biofilm peroxide resistance protein BsmA | -1.91 | 9.38E-03 |
| 8914592 | *clpB* | ATP-dependent chaperone ClpB | 1.81 | 9.41E-03 |
| 8911263 | *EAMY_RS19495* | DUF1062 domain-containing protein | 2.02 | 1.00E-02 |
| 8911500 | *EAMY_RS23355* | hypothetical protein | -2.01 | 1.00E-02 |
| 8912063 | *EAMY_RS31885* | tRNA-Gly | -2.49 | 1.08E-02 |
| 8911912 | *fliR* | flagellar biosynthetic protein FliR | -1.79 | 1.13E-02 |
| 8913104 | *thpR* | RNA 2',3'-cyclic phosphodiesterase | -2.23 | 1.16E-02 |
| 77383449 | *EAMY_RS36795* | hypothetical protein | 1.69 | 1.23E-02 |
| 8913907 | *sapF* | putrescine export ABC transporter ATP-binding protein SapF | -2.34 | 1.28E-02 |
| 8913905 | *sapC* | putrescine export ABC transporter permease SapC | -2.02 | 1.40E-02 |
| 8913838 | *rplF* | 50S ribosomal protein L6 | 1.94 | 1.41E-02 |
| 55585575 | *EAMY_RS36435* | hypothetical protein | -1.56 | 1.46E-02 |
| 8912710 | *dnaK* | molecular chaperone DnaK | 1.56 | 1.55E-02 |
| 23673359 | *EAMY_RS25595* | phosphatidylinositol-specific phospholipase C | 1.50 | 1.56E-02 |
| 8911573 | *EAMY_RS24825* | acyl carrier protein | -3.14 | 1.58E-02 |
| 8912514 | *EAMY_RS23115* | ATP-binding cassette domain-containing protein | -1.97 | 1.58E-02 |
| 8914161 | *EAMY_RS27745* | phosphatase PAP2 family protein | 1.94 | 1.62E-02 |
| 8913891 | *rseB* | sigma-E factor regulatory protein RseB | -2.83 | 1.63E-02 |
| 8912811 | *flgA* | flagellar basal body P-ring formation chaperone FlgA | -1.64 | 1.67E-02 |
| 8914529 | *btsR* | two-component system response regulator BtsR | -3.30 | 1.79E-02 |
| 8912944 | *EAMY_RS21465* | hypothetical protein | 1.61 | 1.90E-02 |
| 8914435 | *EAMY_RS25980* | alpha-glucosidase | 1.67 | 1.91E-02 |
| 8912783 | *fadI* | acetyl-CoA C-acyltransferase FadI | -1.65 | 2.03E-02 |
| 8914217 | *wzyE* | ECA oligosaccharide polymerase | 1.88 | 2.06E-02 |
| 8911276 | *EAMY_RS19630* | spore coat U domain-containing protein | -1.51 | 2.16E-02 |
| 8914960 | *EAMY_RS32835* | DUF1992 domain-containing protein | -3.56 | 2.22E-02 |
| 8912653 | *cysT* | sulfate/thiosulfate ABC transporter permease CysT | -1.68 | 2.32E-02 |
| 55585544 | *EAMY_RS36280* | hypothetical protein | -1.52 | 2.57E-02 |
| 8913091 | *EAMY_RS21005* | hypothetical protein | -2.35 | 2.58E-02 |
| 30316913 | *rprA* | ncRNA | 4.47 | 2.63E-02 |
| 8913932 | *rplO* | 50S ribosomal protein L15 | 2.28 | 2.65E-02 |
| 8913444 | *aspS* | aspartate--tRNA ligase | -1.64 | 2.66E-02 |
| 8912872 | *fliN* | flagellar motor switch protein FliN | -2.38 | 2.68E-02 |
| 30317022 | *EAMY_RS35535* | hypothetical protein | 1.66 | 2.71E-02 |
| 8912854 | *fliE* | flagellar hook-basal body complex protein FliE | -2.71 | 2.86E-02 |
| 8913968 | *EAMY_RS29560* | Tar ligand binding domain-containing protein | -1.83 | 2.87E-02 |
| 8911774 | *EAMY_RS27430* | hypothetical protein | 1.60 | 2.88E-02 |
| 8911444 | *EAMY_RS22400* | tRNA-Arg | -1.64 | 2.90E-02 |
| 8914919 | *EAMY_RS31610* | DoxX family protein | -1.56 | 3.01E-02 |
| 30316919 | *EAMY_RS35020* | hypothetical protein | -2.15 | 3.01E-02 |
| 8914500 | *cmoB* | tRNA 5-methoxyuridine(34)/uridine 5-oxyacetic acid(34) synthase CmoB | 3.54 | 3.03E-02 |
| 8911480 | *uvrB* | excinuclease ABC subunit UvrB | -1.85 | 3.33E-02 |
| 23673427 | *EAMY_RS32590* | pseudo | -2.35 | 3.51E-02 |
| 8913742 | *fliR* | flagellar biosynthetic protein FliR | -1.56 | 3.58E-02 |
| 23673300 | *EAMY_RS17570* | hypothetical protein | -2.43 | 3.83E-02 |
| 69102808 | *kdpF* | K(+)-transporting ATPase subunit F | -2.79 | 3.96E-02 |
| 8912229 | *EAMY_RS34085* | winged helix-turn-helix domain-containing protein | -3.47 | 3.98E-02 |
| 8914625 | *rlmM* | 23S rRNA (cytidine(2498)-2'-O)-methyltransferase RlmM | 2.04 | 3.99E-02 |
| 8914203 | *rfaQ* | putative lipopolysaccharide heptosyltransferase III | -3.94 | 4.02E-02 |
| 8913085 | *lptE* | LPS assembly lipoprotein LptE | 1.53 | 4.02E-02 |
| 8913138 | *hutG* | N-formylglutamate deformylase | -3.45 | 4.23E-02 |
| 8912686 | *zapG* | Z-ring associated protein ZapG | 1.58 | 4.74E-02 |
| 8913833 | *rpsI* | 30S ribosomal protein S9 | 2.80 | 4.75E-02 |
| 8912256 | *rrf* | 5S ribosomal RNA | -3.49 | 4.86E-02 |
| 77383448 | *EAMY_RS36790* | hypothetical protein | -1.77 | 4.97E-02 |

^a^ Gene IDs, locus tags, symbols and functions obtained from the DAVID database for *E. amylovora* CFBP 1430. MSTRG identifiers represent assembled and annotated by StringTie, which were not previously annotated in CFBP 1430 strain. They could be potentially new transcripts, alternative splice variants, or transcripts specific to the experimental conditions.

**Supplementary figures**

**Figure S1. Effect of copper pre-exposure in symptom development on ‘Bartlett’ pear fruitlets.** *E. amylovora* cells grown overnight in LB and LB plus 0.5 mM CuSO_4_ were used for virulence assays on detached pears cv. Bartlett. (A) Necrosed areas in *E. amylovora*-inoculated fruitlets with and without copper pre-exposure. (B) representative images of the results 4 days post-inoculation. Columns and error bars show average values of 6 – 7 fruit and standard deviation. *P* values indicate the significance of copper exposure during growth and the strain in the obtained results, based on a two-way ANOVA. Different letters indicate statistically significant differences between the compared groups of data, determined by Šídák's multiple comparisons tests (α = 0.05).

**
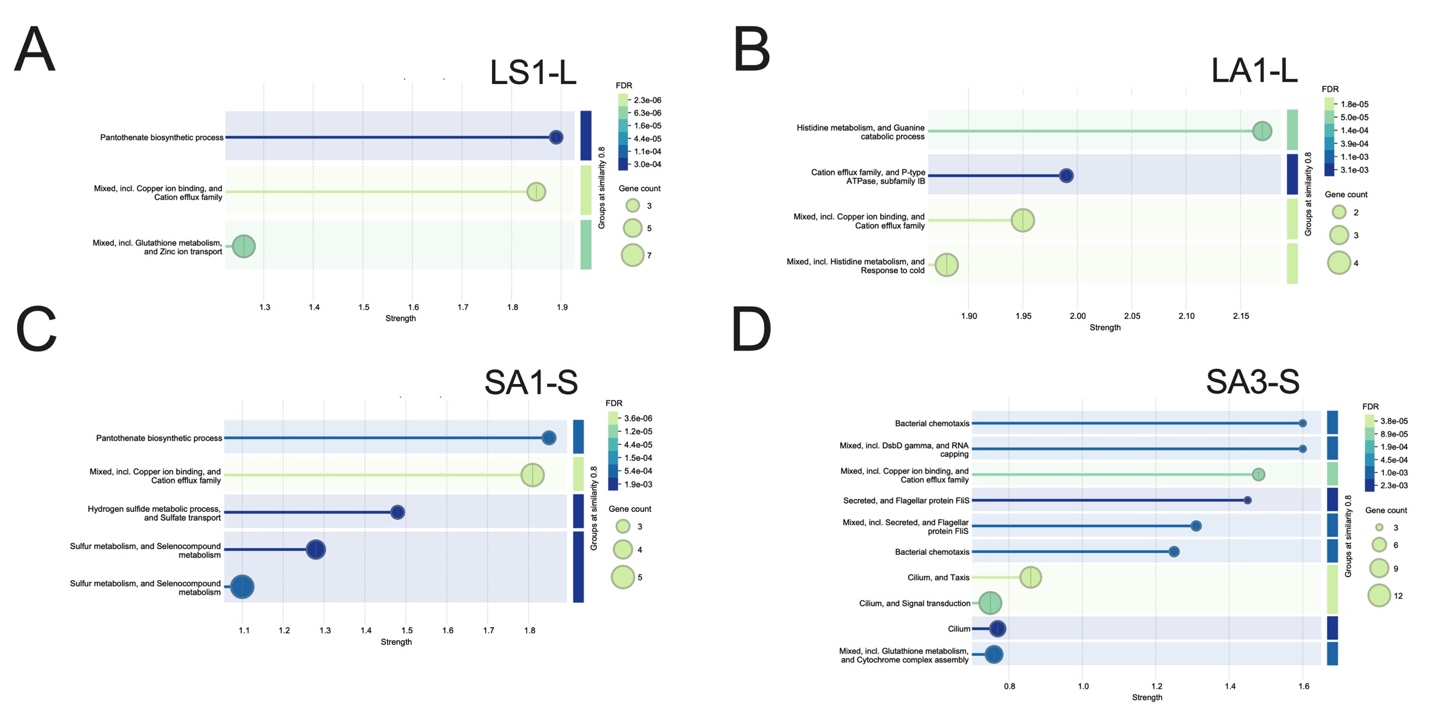
**

**Figure S2. STRING functional association analysis of differentially expressed genes (DEGs) under four experimental conditions (A – D) in *E. amylovora* Ea273.** Each panel represents enriched biological processes or functional pathways, with the x-axis indicating the strength of association (STRING enrichment score) and the y-axis listing the enriched terms. The size of the circles corresponds to the number of genes involved in each process, while the color gradient reflects the false discovery rate (FDR), with darker shades indicating higher significance. The analysis was performed with up- and downregulated DEGs resulting from comparisons between treatments and controls (e.g., LS1-L). Treatment abbreviations: LS1, copper-shock treatment with 1 mM CuSO_4_ for 5 min applied on log-phase cells; LA1, growth with 1 mM CuSO_4_ up to mid-log phase; SA1, growth with 1 mM CuSO_4_ up to stationary phase; SA3, growth with 3 mM CuSO_4_ up to stationary phase. Control comparisons: -L, mid-log phase cells; -S, stationary phase cells.

**
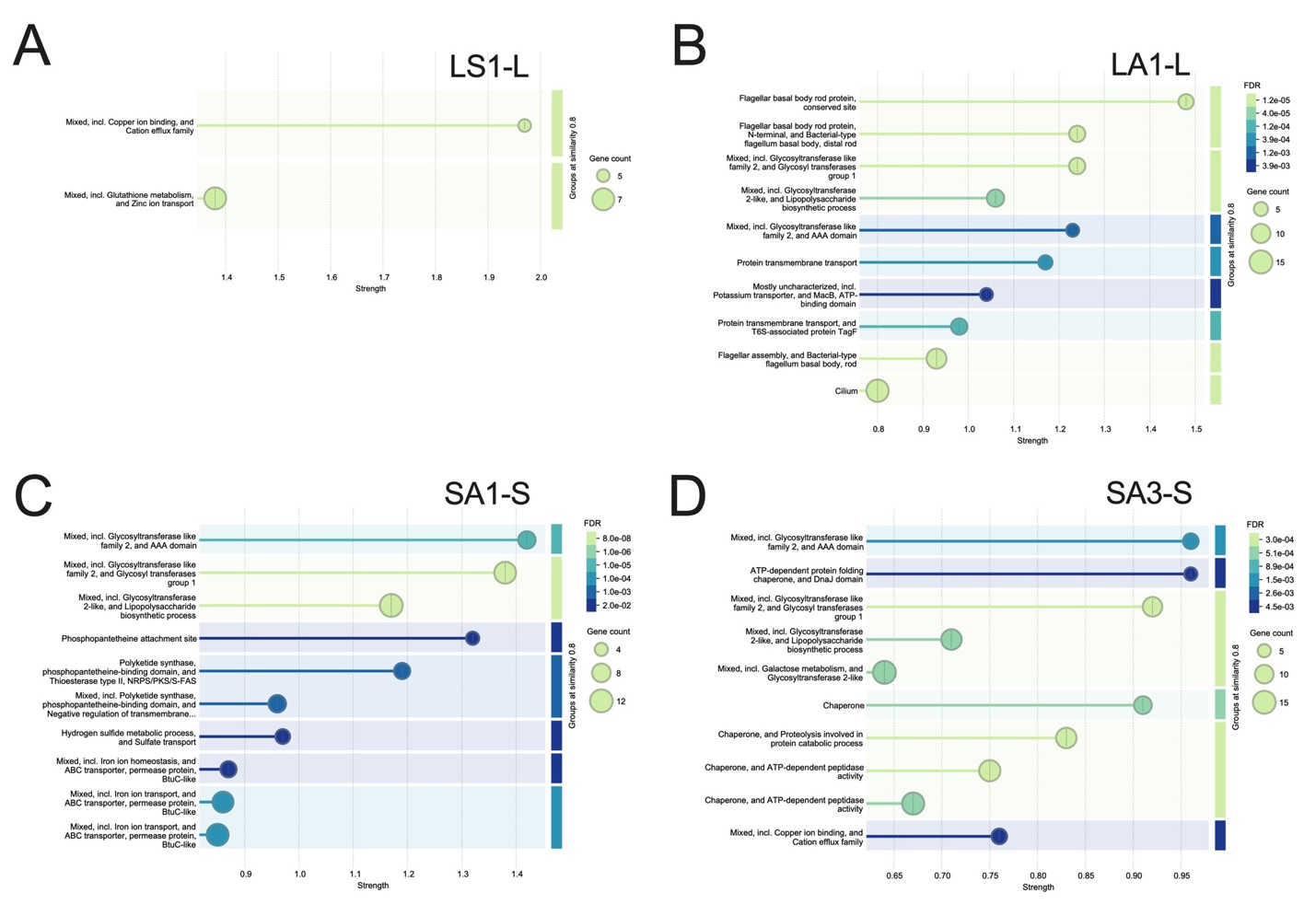
**

**Figure S3. STRING functional association analysis of differentially expressed genes (DEGs) under four experimental conditions (A – D) in *E. amylovora* EaR2.** Each panel represents enriched biological processes or functional pathways, with the x-axis indicating the strength of association (STRING enrichment score) and the y-axis listing the enriched terms. The size of the circles corresponds to the number of genes involved in each process, while the color gradient reflects the false discovery rate (FDR), with darker shades indicating higher significance. The analysis was performed with up- and downregulated DEGs resulting from comparisons between treatments and controls (e.g., LS1-L). Treatment abbreviations: LS1, copper-shock treatment with 1 mM CuSO_4_ for 5 min applied on log-phase cells; LA1, growth with 1 mM CuSO_4_ up to mid-log phase; SA1, growth with 1 mM CuSO_4_ up to stationary phase; SA3, growth with 3 mM CuSO_4_ up to stationary phase. Control comparisons: -L, mid-log phase cells; -S, stationary phase cells.

**

**

**Figure S4. RNA-Seq experimental design comparing copper shock and adaptation responses in *E. amylovora***. To differentiate immediate copper detoxification responses from adaptive strategies during prolonged copper exposure, we compared transcriptomic profiles of copper-tolerant (Ea273) and copper-hypersensitive (EaR2) strains under both copper shock and extended copper exposure conditions. A, Control conditions: cells grown in LB at 28ºC (180 rpm), with samples collected at mid-log phase and stationary phase (20-h). B, Copper-shock conditions: Mid-log phase cultures exposed to 1 mM CuSO_4_ for 5 minutes. C-E, Copper adaptation conditions: C, Cells grown in LB with 1 mM CuSO_4_ to mid-log phase; D, cells grown to stationary phase (20h) in LB with 1 mM CuSO_4_; E, cells grown to stationary phase (20h) in LB with 3 mM CuSO_4_. All treatments included three biological replicates per strain.
